# A global analysis of the impact of COVID-19 stay-at-home restrictions on crime

**DOI:** 10.1038/s41562-021-01139-z

**Published:** 2021-06-02

**Authors:** Amy E. Nivette, Renee Zahnow, Raul Aguilar, Andri Ahven, Shai Amram, Barak Ariel, María José Arosemena Burbano, Roberta Astolfi, Dirk Baier, Hyung-Min Bark, Joris E. H. Beijers, Marcelo Bergman, Gregory Breetzke, I. Alberto Concha-Eastman, Sophie Curtis-Ham, Ryan Davenport, Carlos Díaz, Diego Fleitas, Manne Gerell, Kwang-Ho Jang, Juha Kääriäinen, Tapio Lappi-Seppälä, Woon-Sik Lim, Rosa Loureiro Revilla, Lorraine Mazerolle, Gorazd Meško, Noemí Pereda, Maria F. T. Peres, Rubén Poblete-Cazenave, Simon Rose, Robert Svensson, Nico Trajtenberg, Tanja van der Lippe, Joran Veldkamp, Carlos J. Vilalta Perdomo, Manuel P. Eisner

**Affiliations:** 1 Department of Sociology, Utrecht University, Utrecht, the Netherlands; 2 Netherlands Institute for the Study of Crime and Law Enforcement (NSCR), Amsterdam, the Netherlands; 3 School of Social Science, University of Queensland, Brisbane, Queensland Australia; 4 Mossos d’Esquadra, Catalan Police, Barcelona, Spain; 5 Ministry of Justice, Tallin, Estonia; 6 Institute of Criminology, Faculty of Law, Hebrew University, Jerusalem, Israel; 7 Institute of Criminology, University of Cambridge, Cambridge, UK; 8 Departamento de Medicina Preventiva, Faculdade de Medicina, Universidade de São Paulo, São Paulo, Brazil; 9 Institute of Delinquency and Crime Prevention, Zürcher Hochschule für Angewandte Wissenschaften (ZHAW) School of Social Work, Zürich, Switzerland; 10 Korean Institute of Criminology, Seoul, Republic of Korea; 11 Centro de Estudios Latinoamericano sobre Inseguridad y Violencia (CELIV), Universidad Nacional de Tres de Febrero, Buenos Aires, Argentina; 12 Department of Geography, Geoinformatics and Meteorology, University of Pretoria, Pretoria, South Africa; 13 Secretariat of Health, Cali, Colombia; 14 Evidence Based Policing Centre, New Zealand Police, Wellington, New Zealand; 15 Jill Dando Institute of Security & Crime Science, University College London, London, UK; 16 London Metropolitan Police, London, UK; 17 Department of Social Sciences, Catholic University of Uruguay, Montevideo, Uruguay; 18 Department of Criminology, Malmö University, Malmö, Sweden; 19 Smart Policing Intelligence Center, Police Science Institute, Seoul, Republic of Korea; 20 Institute of Criminology and Legal Policy, University of Helsinki, Helsinki, Finland; 21 Faculty of Criminal Justice and Security, University of Maribor, Maribor, Slovenia; 22 Department of Clinical Psychology and Psychobiology, Universitat de Barcelona, Barcelona, Spain; 23 Erasmus School of Economics, Erasmus University Rotterdam, Rotterdam, the Netherlands; 24 School of Social Sciences, Cardiff University, Cardiff, UK; 25 Center for Research in Geospatial Information Sciences (CentroGeo), Mexico City, Mexico; 26 Jacobs Center for Productive Youth Development, University of Zürich, Zürich, Switzerland

**Keywords:** Criminology, Sociology, Social policy

## Abstract

The stay-at-home restrictions to control the spread of COVID-19 led to unparalleled sudden change in daily life, but it is unclear how they affected urban crime globally. We collected data on daily counts of crime in 27 cities across 23 countries in the Americas, Europe, the Middle East and Asia. We conducted interrupted time series analyses to assess the impact of stay-at-home restrictions on different types of crime in each city. Our findings show that the stay-at-home policies were associated with a considerable drop in urban crime, but with substantial variation across cities and types of crime. Meta-regression results showed that more stringent restrictions over movement in public space were predictive of larger declines in crime.

## Abstract (web summary)

The implementation of COVID-19 stay-at-home policies was associated with a considerable drop in urban crime in 27 cities across 23 countries. More stringent restrictions over movement in public space were predictive of larger declines in crime.

## Main

On 11 March 2020, the World Health Organization declared COVID-19 to be a public health emergency of global concern. Following the WHO declaration, national and local authorities moved to impose a range of measures to slow the spread of the virus (‘flatten the curve’) and alleviate strain on health care systems. Collectively referred to as ‘lockdown’ measures in most countries, regulations have included some combination of stay-at-home orders, travel bans, closures of schools and places of entertainment and restrictions on public and private gatherings. Strategies aimed at limiting the mobility of the entire population through measures that require or recommend that residents do not leave the house except for ‘essential’ activities arguably were among the most intrusive policies, with wide-ranging collateral effects on society, the economy and human rights^1^. Spatial mobility data suggest that, at the peak of the so-called lockdown—in late March and April 2020—daily movements related to retail and recreation had declined by over 80% in many countries in Europe and Latin America^2^.

In this study, we examine the extent to which stay-at-home restrictions in 27 cities in the Americas, Europe, the Middle East and Asia were associated with a change in levels of six types of police-recorded crime. The cities represent a large variation of measures relating to stay-at-home restrictions. They range from mostly voluntary recommendations to avoid public space (for example, Malmö and Stockholm in Sweden) to a complete halt of all but the most essential activities, based on emergency legislation and enforced by substantial penalties for breaching the rules (for example, Lima in Peru). This allows us to move significantly beyond previous studies conducted in single cities to evaluate the generalizability of criminogenic processes triggered by stay-at-home restrictions.

Various theories of crime examine how sudden and persisting restraints on population movements caused by, for example, natural disasters, blackouts or epidemics affect crime levels^3^. Theories of individual and structural strain suggest that such restraints increase levels of stress and negative emotions such as anxiety, frustration and anger, thereby leading to an increase in criminal motivations^4^. In this vein, social isolation and reduced freedom of movement associated with COVID-19 containment policies are anticipated to heighten levels of strain and reduce access to support with implications for child maltreatment^5^, domestic violence^6^ and substance use^7^. Opportunity theory and routine activity theory, in contrast, suggest that stay-at-home policies interrupted the daily movements in time and space of suitable targets, capable guardians and motivated offenders on which most crime, especially crime in public space, feeds^8^. They hence predict that crime levels fall as the mobility of entire urban populations is restricted^9^.

While some early studies suggested that violent and non-violent crime dropped as regulations were imposed, there is also evidence that the effects of COVID-19 on crime are not universal across countries nor across different categories of crime^10–12^. Rather, opportunity structures are specific to different types of crimes, and a change in opportunities for theft may not correlate with a change in opportunities for assault^13^. For example, opportunities for certain property crimes, such as theft and robbery, depend on the daily flow of people into commercial areas and nearby transportation nodes that offer a high volume of suitable targets and access/exit paths for motivated offenders, and may hence have declined particularly strongly as a result of the lockdown measures^14,15^. Similarly, as most people stayed at home throughout the day, fewer houses were left unsupervised and residential burglary may have become much more difficult, while commercial buildings likely became less supervised and hence an easier target^9^. Also, while the shutdown of night-time leisure activities and alcohol consumption in urban centres greatly reduced the potential for violent conflict among young men in public spaces, the potential for domestic violence increased as victims found it harder to find help and support^16^. Finally, police services have also been required to adjust priorities and redistribute resources to carry out quarantine checks, enforce social distancing and enact border control^17^.

## Results

### The impact of stay-at-home restrictions on crime

The COVID-19 pandemic and subsequent restrictions represent a series of ‘natural experiments’ in which population-wide changes affected routines, social interactions and the use of public space. An interrupted time series (ITS) design can then be used to assess the impact of the treatment while accounting for pre-COVID-19 crime trends^18^. ITS analyses provide an estimate of changes in levels of crime following an ‘interruption’ in the time series, while accounting for potential confounders such as long-term trends, autocorrelation and other time-varying confounders^19^. In an ITS analysis, the assumption is that, without the intervention (that is, COVID-19 restrictions), there would be no change in the pre-intervention trend^18^.

The dependent variable in our analyses is police-recorded daily reported crime incidents for six major crime categories: assault, theft, burglary, robbery, vehicle theft and homicide. To ensure that the crime categories were as comparable as possible across contexts, we utilized definitions from the International Classification of Crime for Statistical Purposes^20^ for reference when collecting and aggregating crime data from each site (Supplementary Table 3). Not all crime categories were available for each city, and in some contexts certain crimes are not treated as separate categories. For example, in Seoul, burglary is not considered separately from robbery, and motor vehicle theft is not distinguished from theft. To ensure that the crime categories are as comparable as possible, we excluded these combined outcomes from the analyses (Supplementary Tables 4–10).

The treatment variable for the current analyses is a dummy variable defined by the date on which stay-at-home restrictions or recommendations were first implemented in each city, state/province or country (Supplementary Table 11). The effects of stay-at-home restrictions are modelled as a step function, whereby 0 represents the time period before and (if applicable) after the implementation of stay-at-home restrictions, and 1 represents the time period during stay-at-home restrictions. In this way, the step function estimates the extent to which the restrictions had an immediate effect on crime during the intervention period.

Given the count nature of our data, and the variation in frequency of daily crime incidents across cities (ranging from 0 to >500 daily incidents), in the present analyses we estimated Poisson generalized linear models using a logit-link function. This flexible approach provides an estimate of the level change in crime incidents after the implementation of stay-at-home restrictions. All tests are two-tailed, and models adjust for seasonality, autocorrelation and potential outliers. In addition, we included average daily temperature in Celsius as a covariate to account for potential fluctuations in crime due to higher temperatures^21^.

As an initial step, we conducted a series of descriptive analyses to evaluate the changes in crime before and after the implementation of COVID-19 stay-at-home restrictions. First, we calculated the average number of crimes in each city and category before and after the implementation of restrictions (Supplementary Table 15). Second, we plotted the 7-day moving average of daily crime counts for each city and crime. The moving average trend was indexed to equal 100 at the date on which the first stay-at-home restrictions were implemented. In this way, we can compare the direction of the trend immediately before and following restrictions across cities with different levels of crime. The mean trend for each type of crime was plotted alongside each city’s trend (Fig. 1). Supplementary Figs. 1–6 present the moving average trends broken down by city and type of crime. The full (non-smoothed) time series plots for each city and type of crime can be found in Supplementary Figs. 7–25.

**Fig. 1:**
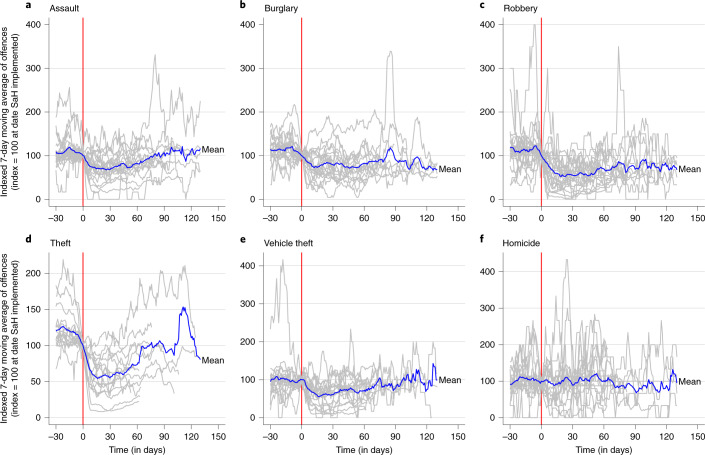
Seven-day moving average time series plots of daily numbers of crimes. **a,** Assault (*n* = 23). **b**, Burglary (*n* = 20). **c**, Robbery (*n* = 24). **d**, Theft (*n* = 16). **e**, Vehicle theft (*n* = 20). **f**, Homicide (*n* = 25). Each time series is indexed to equal 100 on the day the first stay-at-home restrictions were implemented. The blue line indicates the average trend across all cities with available data. Zero time is the date on which stay-at-home restrictions were implemented.

The descriptive results suggest that stay-at-home restrictions are associated with declines in all types of crime, with the exception of homicide. In Barcelona, for example, police-recorded thefts declined from an average of 385.2 to 38.1 per day (Supplementary Table 15). However, there still appears to be substantial variation across crime categories and cities in the size and direction of crime trends following the implementation of restrictions. Over time, the mean trend begins to return to pre-treatment levels of crime.

**Fig. 2:**
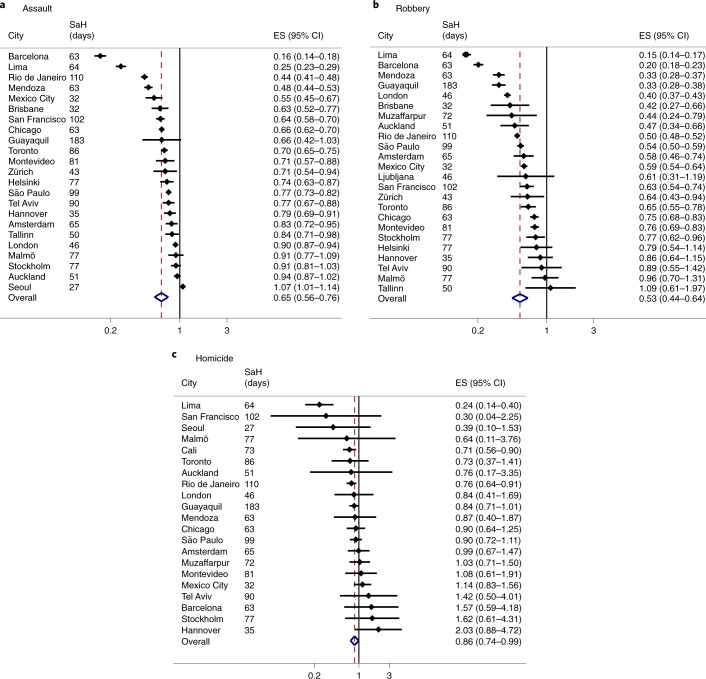
IRR and 95% CI of stay-at-home restrictions on daily number of violent crimes. **a**, Assault (*n* = 23). **b**, Robbery (*n* = 24). **c**, Homicide (*n* = 21). Overall summary effects estimated using random-effects meta-analytic techniques. ES, effect size. SaH (days), number of days under stay-at-home restrictions from the beginning of 2020 until the end of the respective time series from May to September 2020. Full results by city and crime can be found in Supplementary Table 17.

**Fig. 3:**
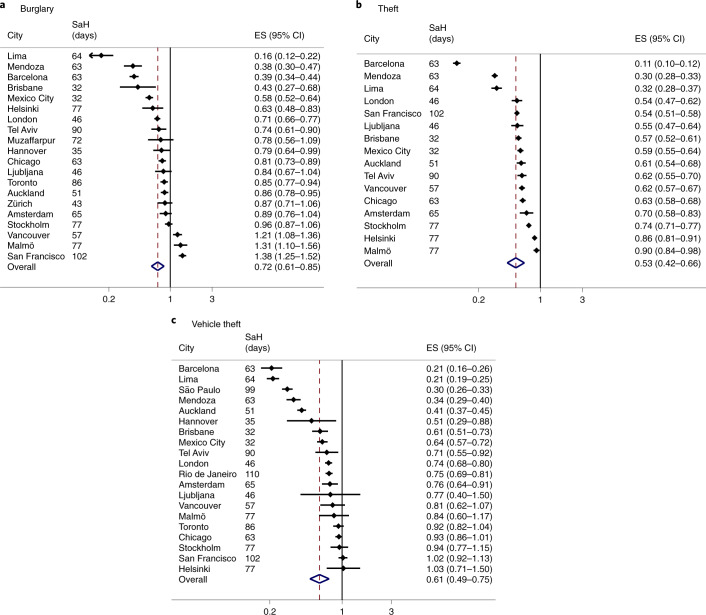
IRR and 95% CI of stay-at-home restrictions on daily number of property crimes. **a**, Burglary (*n* = 20). **b**, Theft (*n* = 16). **c**, Vehicle theft (*n* = 20). Overall summary effects estimated using random-effects meta-analytic techniques. ES, effect size. SaH (days), number of days under stay-at-home restrictions from the beginning of 2020 until the end of the respective time series from May to September 2020. Full results by city and crime can be found in Supplementary Table 17.

Next, we estimated the size of the level change in daily crimes that can be attributed to stay-at-home restrictions using ITS analysis. The analyses of trends for six categories of police-recorded daily reported crime incidents across 27 cities result in over 100 estimates of effect. To summarize this information, we used meta-analytical techniques to estimate the grand mean effect of stay-at-home restrictions for each type of crime (Table 1). The estimates of effect, expressed as the incidence rate ratio (IRR) with 95% confidence interval, are presented in Figs. 2 and 3 for violent and property crimes, respectively. The high number of hypotheses tested increases the possibility that we may detect a significant result due to chance. We therefore urge caution in interpreting the results for individual cities. The breakdown of effect sizes and summary effects by city are available in Supplementary Table 16. Across our sample, crime declined overall by 37% following the implementation of stay-at-home restrictions.

**Table 1:** Summary effect sizes from meta-analyses using cities with any available crime categories

	Overall ES	95% CI lower	95% CI upper	Heterogeneity statistic	*P*-value	*τ*^2^	*I*^2^	*N*
Assault	0.65	0.56	0.76	1,402.34	<0.001	0.14	98.40%	23
Burglary	0.72	0.61	0.85	561.64	<0.001	0.13	96.60%	20
Robbery	0.54	0.45	0.64	1,103.28	<0.001	0.19	97.90%	24
Theft	0.53	0.42	0.66	1,864.56	<0.001	0.21	99.20%	16
Vehicle theft	0.61	0.49	0.75	916.79	<0.001	0.22	97.90%	20
Homicide	0.86	0.74	0.99	44.03	0.001	0.05	54.60%	21

The summary results here are the same as presented in Figs. 2 and 3. ES, effect size; CI, confidence interval. *P* values refer to the heterogeneity statistic. Overall summary effects estimated using random-effects meta-analytic techniques.

For assault, the summary effect suggests that the implementation of stay-at-home restrictions was associated with a 35% reduction in daily assaults (Fig. 2a). The *I*^2^ value of 98.4% suggests substantial heterogeneity in the effect sizes across cities and crime outcomes (Table 1). Similarly, effect sizes for robbery vary across cities, however no cities experienced a statistically significant increase in the number of daily robbery incidents following restrictions. The average size of the level change following restrictions was 46%.

The results for homicide suggest that overall there was a marginal decline in the number of daily homicides following the implementation of stay-at-home restrictions (14%, Fig. 2c). However, only three cities (Lima, Cali and Rio de Janeiro) saw a statistically significant decline in homicides. The *I*^2^ statistic (54.6%, Table 1) indicates relatively less heterogeneity in effects compared with other crime outcomes.

The distribution of effect sizes for burglary ranges from an 84% decline (Lima) to a 38% increase (San Francisco) in the number of daily incidents. The summary effect is relatively smaller compared with assault, where on average burglaries fell by 28% following the implementation of restrictions.

All cities with available data on theft experienced a significant drop in the number of daily incidents, however the *I*^2^ statistic (99.2%) still indicates high levels of heterogeneity between cities. Even cities with less restrictive, more voluntary stay-at-home recommendations (for example, Malmö and Stockholm) experienced marginal declines in the number of daily thefts. The results for vehicle theft also suggest heterogeneity in effects across cities, with 8 out of 18 cities experiencing no statistically significant change in the number of incidents following restrictions. The mean drop in vehicle thefts across cities was 39%.

### Stringency of restrictions and size of decline

The next step is to evaluate why we find such substantial heterogeneity in effect sizes across cities. Heterogeneity in effect sizes can be attributed to, for example, variations in the characteristics of local or national policies. We estimate the extent to which variations in effect sizes are associated with the stringency of stay-at-home restrictions and wider COVID-19-related containment policies. To measure stringency, we drew from the Oxford Government Response Tracker documentation and coding of COVID-19 policy responses^22^. The stringency of stay-at-home restrictions is measured on a scale from 0 (no measures) to 3 (do not leave the house with minimal exceptions).

For the current analyses, we took the average of the stay-at-home scores between the first day of implementation to either the lifting of restrictions or the end of the time series, whichever came first (Supplementary Table 11). Using mixed-effects meta-regression techniques, we are able to assess whether more severe restrictions on routine activities are associated with greater declines in daily crimes (that is, larger negative effect sizes).

The results in Table 2 suggest that more stringent stay-at-home restrictions were associated with significantly more negative effect sizes for burglary, robbery, theft and vehicle theft. In essence, this suggests that more severe restrictions on ‘non-essential’ movement and activities were associated with significantly larger declines in crime. While the coefficients are negative for assault, the association was not significant at the conventional threshold of 0.05. However, inspection of the scatterplots suggests that Barcelona may be an outlier (Supplementary Fig. 26). When Barcelona is excluded from meta-regression analyses, more stringent stay-at-home restrictions are negatively associated with effect sizes for assault (Supplementary Table 19). The adjusted *R*^2^ values for burglary and robbery show that the stringency of stay-at-home restrictions accounts for about 35% of the variation in effect sizes across cities.

**Table 2:** Meta-regression results for the stringency of stay-at-home restrictions and overall stringency index on the size of the effect of stay-at-home orders on police-recorded crime

		Assault	Burglary	Robbery	Theft	Vehicle theft	Homicide
Stringency of stay-at-home restrictions	*b*	−0.25	−0.37	−0.40	−0.33	−0.39	−0.26
	s.e.	(0.13)	(0.12)	(0.12)	(0.14)	(0.14)	(0.16)
	*P* value	0.06	0.01	0.002	0.03	0.01	0.12
	95% CI	−0.52 to 0.01	−0.61 to −0.12	−0.64 to −0.16	−0.63 to −0.04	−0.68 to −0.11	−0.59 to 0.08
	exp(*b*)	0.78	0.69	0.67	0.72	0.67	0.77
	*τ*^2^	0.16	0.15	0.14	0.19	0.18	0.07
	Adj. *R*^2^	12.49%	34.42%	35.92%	24.33%	28.67%	18.59%
	*N*	23	20	24	16	20	21

Exp(*b*) reflects the standardized exponentiated coefficient. Adjusted (adj.) *R*^2^ reflects the proportion of variance in the effect sizes explained by the given covariate.

### Additional analyses

As an additional set of analyses, we evaluated the possibility that other COVID-19-related policy responses may account for variations in the size of the effect instead of, or in addition to, stay-at-home restrictions. For example, based on strain perspectives, we may expect smaller declines in cities and contexts where there is less economic support for those affected by unemployment or financial strain due to the pandemic. This would be because individuals experiencing strain are motivated to cope by seeking out alternative, possibly illegal income opportunities. In addition, since stay-at-home restrictions were often implemented alongside a wide range of policies that regulated leisure activities, routines and opportunities, we also examined the relationship between the overall stringency index and effect sizes for each type of crime.

The results show that more severe restrictions on school opening, working from home, public events, private gatherings and internal movement are not significantly related to the size of effects (Supplementary Table 21), with one exception: More stringent reductions or closures of public transportation are associated with more negative effect sizes for robbery and vehicle theft only. More economic support was not associated with the variation in effect sizes for any type of crime. The results for the overall stringency index were generally in line with the main findings for stay-at-home restrictions, whereby more stringent combinations of containment policies were associated with greater declines in burglary, robbery, vehicle theft and theft. However, comparing the model fit (adjusted *R*^2^ values) suggests that accounting for the overall combined policy response does not substantially improve the model fit.

Further, while the stringency indices and sub-indices provide systematic and comparable measures of COVID-19 containment policies across countries, they do not provide a measure of actual behavioural changes. We therefore conducted additional analyses to assess the relationship between changes in mobility indices as measured by the Google COVID Community Mobility Reports^23,24^, and effect sizes for each type of crime. Bivariate correlations between mobility measures and stringency measures suggest that more stringent stay-at-home restrictions are associated with greater declines in visits to commercial locations and parks, as well as increases in users remaining in their residences (Supplementary Table 14). The results using mobility indices are generally in line with the results using the stringency index measures, whereby cities that saw greater declines in the use of public space saw larger declines in crime, with the exception of homicide (Supplementary Table 22).

## Discussion

In this study, we examined trends in police-recorded crime in the period after the introduction of stay-at-home policies in 27 cities worldwide. Our findings show that the stay-at-home policies were associated with a substantial drop in urban crime. On average, the overall reduction in crime levels across all included cities was −37%. They suggest that the sudden decline in urban mobility triggered by the stay-at-home policies reduced opportunities and increased guardianship relating to many high-volume crimes. In other words, as expected by economic and criminological opportunity theories, we found strong evidence that crime levels respond quickly to changing opportunity structures and constraints, and that change in crime levels does not necessarily require large-scale changes in offender motivation^15^. At least in the short run, the change in routine activities rather than the increase in psychological and social strains was the dominating mechanism that affected change in overall crime levels. We did not find evidence for or against displacement effects in the sense of a shift from one type of crime to another within the categories of crime covered in this paper. However, the lack of high-quality comparable data means that we could not examine the possibility that a substantial amount of coercive and property crime moved online, parallel to the sudden shift in daily routine activities.

Visual inspection of crime trends anchored by the beginning of the stay-at-home orders suggests that the declines were short-lived, with a maximum drop around two to five weeks after the implementation of the measures and a gradual return to previous levels in the subsequent weeks (Fig. 1). This aligns with previous research conducted in Australia^25^ and China^26^ that found that immediate declines in public-space crimes such as theft, burglary and traffic offences experienced during lockdown periods quickly reversed as restrictions eased. Future research should examine whether these longer trends in crime levels reflect the gradual relaxation of the constraints during June, July and August of 2020, or whether they rather signal the slower build-up of strains due to the social and economic disruption experienced especially by disadvantaged young people.

Across cities, the rapid deceleration of urban activity had comparable effects on similar crime categories, despite variation in size, geographic location and social structure. The average reduction was smallest for homicide with −14%. It was largest for robbery and theft with −46% and −47% respectively, with the reductions for burglary (−28%), vehicle theft (−39%) and assault (−35%) in between. We observe the largest effects for crimes that involve the convergence of motivated offenders and suitable victims/targets in public space, likely because far fewer potential victims spent time in crime hotspots such as inner-city areas with concentrations of businesses and entertainment venues^2^. Also, efforts to monitor compliance with stay-at-home regulations probably increased levels of formal social control in urban space^9^.

In contrast, reductions were much more limited for homicide. The smaller decrease in homicide cases may be due to a number of factors. First, in many societies, a substantial proportion of homicides are committed in domestic contexts and are hence not affected by the reduction in the density of daily encounters in cities. Second, a varying proportion of homicide is associated with organized crime, conflicts between gangs or conflicts related to drug trafficking. The behaviour of these groups may be less elastic to changes in the daily routines of those not involved in organized crime. In this vein, conventional crimes in Mexico City declined while crimes associated with organized crime (homicide, extortion and kidnappings) did not^12^. However, this argument does not always hold. More specifically, in three of the studied cities (Cali, Lima and Rio de Janeiro), a large proportion of homicides are committed by gang members. However, homicide levels dropped substantially in each city after the stay-at-home orders. One possible explanation is that criminal groups used the crisis to strengthen their power by imposing their own curfews and restricting movement in the territories they control^27^.

The reduction in burglaries is likely related to increased informal social control in that more dwellings were occupied around the clock, hence offering fewer opportunities for burglaries with a low risk of being disturbed. However, we note that, for many cities, it was not possible to distinguish residential and commercial burglaries. Additional analyses for cities where such a separation is possible suggest that, in line with these arguments, commercial burglaries were largely unaffected by the stay-at-home orders while burglaries against private premises were more likely to decline (Supplementary Fig. 28).

**Fig. 4:**
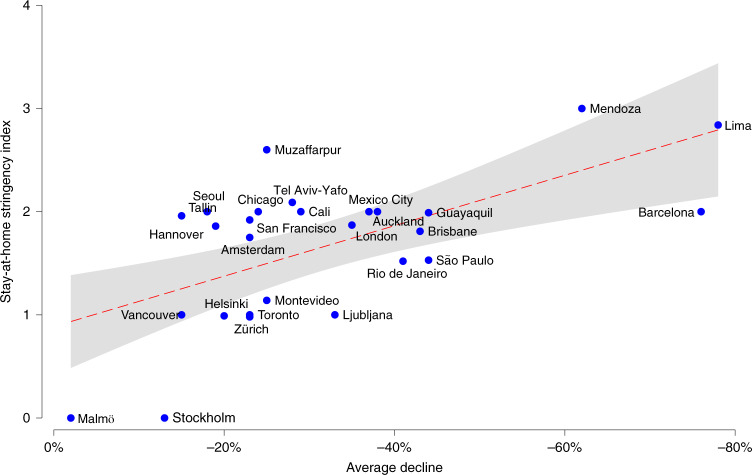
Scatterplot depicting bivariate correlation between overall average decline in crime by city and stringency of stay-at-home restrictions (*n* = 27). The overall average decline was computed using the summary effect sizes for each city reported in Supplementary Table 17, with predicted linear relationship between average decline and stay-at-home stringency index (dotted line and shaded area) and 95% confidence intervals (shaded area).

Finally, we examined whether variation in the stringency of the lockdown was predictive of the amount of change in crime. Results show that more stringent limitations regarding requirements/recommendations to stay at home were associated with stronger declines in crime levels (Fig. 4). The additional analyses suggest that it is mostly the stay-at-home requirements that were associated with larger declines, in that other containment policies were generally not significantly associated with declines, and the use of the overall stringency index generally did not substantially improve the models (Supplementary Table 21). We found few systematic differences in the ‘elasticity’ of different crime categories, that is, in the extent to which variation in the stringency of COVID-19-related restrictions was associated with change in crime levels. This suggests, surprisingly perhaps, that all crime categories included in this analysis responded similarly to variation in the extent of constraints on daily movement.

One must bear in mind that the comparative focus of the present analyses made it impossible, for example, to conduct more fine-grained analyses by contextual characteristics. We would expect, for example, that a distinction of assault cases by place would reveal that assault in the hotspots of weekend night-time activities declined more where the lockdown was more stringent, while violence in domestic contexts may not have declined or may even have increased. Our results might be hiding a more complex picture characterized by neighbourhood heterogeneity in terms of both the independent and the dependent variables. Research in Chicago shows that there is heterogeneity in the impact of containment policies across communities and only a small percentage of communities experienced significant reductions in crime. Variation depended on the type of crime (for example, burglaries, assaults, narcotic-related offences and robberies), community crime characteristics (for example, previous levels of offences, perception of safety and presence of police station) and socioeconomic characteristics (vacant housing, income diversity, poverty, age structure of neighbours and self-perceived health of neighbours)^28,29^. What is more, research in India has shown that higher stringency of lockdown restriction across city districts is associated with lower levels of economically motivated crimes and higher levels of violence against women^30^. Further research on variations within cities and at micro-places is needed to provide further insights into the moderating effect of local contexts on the effects of COVID-19 restrictions on crime.

While the results presented here extend knowledge on the impact of COVID-19 restrictions on crime across international contexts, the study is not without limitations. We acknowledge that the sample of cities included in the analyses is non-random and dominated by cities situated within Europe and the Americas. Further, relying on officially recorded crime data is associated with issues of under-reporting and variations in crime definitions and operational priorities. Police records have well-known problems of reporting/recording, which depends on the type of crime, willingness of victims to report, how criminal justice and health agencies work and their institutional practices, which might be heterogeneous and particularly more problematic in low- and middle-income societies^31^. These measurement problems might be more accentuated during the pandemic given that it might affect victims’ willingness to report crimes^32^. Also, police responses to crime might also change because of staff absences due to COVID-19, increased fear of contracting the virus or even diversion of police resources to alternative tasks such as enforcing the lockdown^25,30,33^. However, studies that use alternative sources have partially validated our results. A recent study in Wales used emergency department visits for violence-related injuries to show that lockdown measures had an impact on the decrease of violence outside the home while no significant differences were observed in violent events at home^34^. We also acknowledge that identifying the specific policy components that affected crime levels remains a challenge in macro-level comparative analyses. Across countries, a range of measures that affect the daily movement of citizens were implemented broadly at the same time. Our analyses suggest that stay-at-home policies played a crucial role. However, more fine-tuned analyses would be needed to understand the extent to which other measures (for example, closing bars, limiting public transport and closing schools) and variation in their enforcement were associated with variation in crime trends across places within a city.

An important area for future comparative research is to investigate the potential displacement of public-space crimes to non-contact offences such as fraud and cybercrime, which we were unable to measure here. Studies conducted within the context of individual countries provide some evidence of displacement from public-space crimes to domestic violence^32,35^. There is some initial evidence of a significant increase of cybercrime during the strictest period of lockdown in the United Kingdom, which is interpreted as a displacement of crime opportunities from the offline to the online environment^36^. Restrictions on public space may have also led to displacement of crime to private space. A recent meta-analysis by Piquero and colleagues suggests that there is strong evidence showing an increase of domestic violence during the pandemic using studies with multiple sources other than police reports (for example, emergency hotline registries, health records and other administrative documents)^32^. This suggests that future research should consider the impact of restriction stringency across cities and countries on the extent of shifts in crime from the public to the domestic sphere.

Finally, it is important to emphasize that the impact of COVID-19-related containment policies on crime trends must be considered within the broader context of global declines in some types of crime, including homicide^37–41^ and vehicular theft^42^, allied with increases in technology-facilitated offences and the potential accelerating effect of the pandemic on this trend.

The measures taken by governments across the world to control COVID-19 continue to have a profound impact on all aspects of social life. They are an opportunity to add to our understanding of social processes, including those involved in the causation of city-wide crime levels. As the crisis progresses, cities and countries continuously adapt their public health strategies. A crucial next step will be to examine longer-term dynamics in more cities globally. Also, we need to complement the comparative macro-level analyses presented here with analyses of how the control measures have affected crime trends in micro-contexts such as crime hotspots.

## Methods

### Crime data

Daily crime data were collected from 27 cities representing 23 countries around the world. The cities were selected in an attempt to maximize geographical coverage and capture a range of policy responses aimed to reduce the transmission and spread of COVID-19. We sought daily crime data on assault, burglary, robbery, theft, vehicle theft and homicide for the current analyses.

Attempts were made to gather daily data from Guatemala, Jamaica, Romania, Norway, Italy, Jordan, South Africa, Ghana, India, the Philippines, Taiwan, China and Japan. However, data were not accessible due to non-response, unavailability of data at a daily interval or refusal. Data for San Francisco, Chicago, Vancouver, Toronto, Muzaffarpur, Brisbane, Auckland, Mendoza and Mexico City were publicly available on police department or city websites^43–51^.

Data for Mendoza refer to Mendoza Province, in which the majority of the population reside in the metropolitan area of Mendoza. Daily data in Mendoza Province are collected primarily from the metropolitan area for technical and procedural reasons, meaning that the data largely reflect the urban population in the province. Data for Muzaffarpur refer to Muzaffarpur District, in which roughly 9% (473,000) live in urban areas within the district with the remaining population residing in rural areas^52^. The data for Zürich refer to the cantonal territory, which is predominantly urban. Additional information regarding sources and definitions can be found in Supplementary Tables 2–9.

The ‘date’ of the time series refers to the date the offence presumably occurred, as recorded by the police. In cases where this information was not available (that is, Amsterdam and São Paulo), the date of reporting was used. In Mexico City, observations refer to the number of criminal investigations initiated. Since not all reported crimes are investigated, in this case the number may under-represent the volume of crime reported to the police. For most cities, the time series starts on 1 January 2018 or 2019 and ends on the most recent date available. Time series information and available crime categories for each city are presented in Supplementary Table 10.

### The ‘treatment’ variable

#### COVID-19 stay-at-home restrictions

The treatment effect of a city’s stay-at-home restrictions on crime incidents is measured using a dummy variable, whereby 1 represents the period in which restrictions were in place and 0 represents the period prior to (or following) the implementation of restrictions.

The date on which restrictions or recommendations were implemented is not always clear-cut across cities. In some cases, restrictions were implemented piecemeal on a local level over time, and in other cases policies were implemented nationwide at once. In these less clear-cut cases, we relied on information from our local collaborators, complemented by information from the Oxford COVID-19 Government Response Tracker^22^ as well as local media resources. Supplementary Table 11 provides summary information including the start and (where relevant) end date of the COVID-19 responses for each city, with a focus on stay-at-home restrictions.

### Covariates in ITS models

Climate data for cities were drawn from the National Centers for Environmental Information^53^. Where information was missing for certain cities and dates, we manually extracted data from Weather Underground (www.wunderground.com). Climate data for Lima, Peru were not available from 1 January 2018 to May 2018.

In addition, we include yearly population as an offset in all models. Population data for each city were drawn from the United Nations’ World Population Prospects^54^. Population data for Ljubljana, Tel Aviv-Yafo and Guayaquil were drawn, respectively, from the Republic of Slovenia’s Statistical Office^55^, the Israel Central Bureau of Statistics^56^ and the National Institute of Statistics and Censuses of Ecuador^57^, respectively. Projected population data for Muzaffarpur were drawn from the IndiaGrowing website^52^.

### Interrupted time series analyses

The ITS analyses were estimated using Poisson generalized linear models with a logit-link function. An important potential confounder in ITS stems from seasonal or long-term trends^18,19,58–61^. Seasonality can typically be visually identified by cyclical patterns^58^. For daily crime data, there are several potential seasonal patterns that must be addressed. Crime patterns have been found to increase periodically during summer months^59^, and certain crimes, such as assaults, are more likely to occur during weekends compared with weekdays^60^. Based on visual inspection of the time series plots, we controlled for seasonal trends using a series of dummy variables representing month of the year, week of the year and/or day of the week^58–61^.

Another methodological issue to address in ITS analyses is autocorrelation. Autocorrelation refers to the similarity between two observations, which violates the assumption of independence^58^. It is possible to identify systematic patterns of autocorrelation between residuals, which can then be accounted for within the regression model for more accurate estimation of effects^62^. Two common models refer to autoregressive processes and moving average processes. An autoregressive process identifies correlations between lagged observations and is modelled by including past values of the outcome in the regression model^58,62^. A moving average process refers to systematic patterns in the residuals, which can be modelled by including terms for relevant lagged residuals into the regression model^58^.

Patterns of residual autocorrelation were evaluated by inspecting the partial autocorrelation function and autocorrelation function plots. Once any autoregressive and moving average processes were identified and accounted for in the model, these plots, as well as multivariate portmanteau (Q) statistics, were used to determine the extent to which the residuals were ‘white noise’, meaning all processes have been accounted for and there is no significant, systematic autocorrelation between the residuals^63,64^. When two models fitted similarly well, we chose for the more parsimonious model with the lowest Akaike information criterion value.

In addition to the above methodological issues, we took several steps to improve the estimation model and fit for each time series. Prior to modelling, a Dickey–Fuller test was used to test for non-stationarity in the time series. Any outliers, defined by significant spikes or dips, were modelled using dummy variables. This includes any holidays where crime incidents are likely to be higher (for example, carnival) or lower (for example, Christmas holidays and New Year’s Day) than usual. Following recommendations, we also included a scaling adjustment to each model to correct for over-dispersion and more accurately estimate standard errors^19^. All models included an offset for population by year.

In some cities, the frequency of crimes per day was almost zero, drawing into question the approriateness of conducting daily time series analyses. This occurred most often for homicide (in Brisbane, Helsinki, Ljubljana, Tallinn and Vancouver), where the daily average number of incidents ranged from 0 to 0.038. The number of assault incidents in Ljubljana during the time period was also near 0. When incidents are sparse, the model may become unstable and unreliable^65^. As such, we opted to exclude these cases from time series analyses.

Additional information on the ITS models can be found in the Supplementary Materials.

### Meta-analyses

Due to the heterogeneous nature of lockdowns and crime definitions across countries, we used random-effects models to estimate summary effects. The random-effects approach assumes that effects vary in part due to characteristics of the treatment effect^66^. In this case, the stay-at-home restrictions varied considerably by the content and implementation of policies, as well as enforcement, which may impact the size of the effect. Random-effects models allow for a distribution of ‘true’ effects whereby the summary effect reflects the mean of this distribution^66,67^. The *I*^2^ statistic displays the percentage of variation that can be attributed to heterogeneity, whereby values above 75% indicate high heterogeneity between results^67,68^.

### Meta-regression

#### COVID-19 policy variables

To examine the factors associated with the size of the effect of stay-at-home restrictions on crime trends, we utilized the Oxford Government Response Tracker’s coding of containment and economic policies^69^. Our focal analyses used the stringency of stay-at-home restrictions, measured as 0 for no measures, 1 for recommendations to not leave the house, 2 for requirements to not leave the house except for ‘essential’ activities and 3 for requirements to not leave the house with minimal exceptions.

In a meta-regression, the dependent variable is the estimated effect size for each city and crime category. In a mixed-effects meta-regression, two error terms are included in the estimation equation: the first is attributable to sampling error, and the second error is associated with deviation from the distribution of ‘true’ effect sizes^66,70^. Due to the small number of effect sizes included in each model, we estimated the effects of each policy variable separately.

### Sensitivity analyses

A series of analyses were conducted to assess the sensitivity of meta-regression results to issues related to the operationalization of COVID-19 policy restrictions, outliers and differing definitions or categorizations of crimes across countries. Specifically, we assessed the extent to which results are sensitive to the use of cities with all available data (Supplementary Table 18), the exclusion of potential outliers (Supplementary Table 19) and the exclusion of cities where domestic or family assault is not distinguished from non-domestic assault incidents in police data (Supplementary Table 20).

### Additional analyses

#### Policy variables and mobility indices

As additional analyses, we examined six separate containment policies, the overall stringency index, economic support policies and Google COVID-19 mobility indices in the meta-regressions. Descriptive statistics for each policy and index for all available cities are available in Supplementary Tables 12 and 13. This allows us to evaluate the extent to which the variations in the size of the effect can be attributed to the stringency of stay-at-home policies compared with other forms of containment or the combination of containment policies, including the stringency of school closures, workplace closures, restrictions on public events, restrictions on private gatherings, restrictions on public transportation and restrictions on internal travel (Supplementary Table 21). The COVID-19 mobility indices include measures of change corresponding to a number of public and private places, including retail and recreation, grocery and pharmacy, parks, workplaces, transit stations and residential places (Supplementary Table 22).

### Reporting summary

Further information on research design is available in the Nature Research Reporting Summary linked to this article.

## Supplementary information

Supplementary information Supplementary Materials and Methods, Supplementary Text, Supplementary Figs. 1–28, Supplementary Tables 1–22 and Supplementary Refs. 1–51.

Reporting summary

Peer review information

## Data Availability

All data used in the analyses have been deposited in the Data Archiving and Networked Services data repository (10.17026/dans-xuf-a75p) for purposes of reproducing or extending the analysis.

## Code availability

All statistical code used in the analyses has been deposited in the Data Archiving and Networked Services data repository (10.17026/dans-xuf-a75p) for purposes of reproducing or extending the analysis.
