# Supplementary material for: A global analysis of the impact of COVID-19 stay-at-home restrictions on crime
Source: Nat Hum Behav. 2021 Jun 2;5(7):868–77. doi: 10.1038/s41562-021-01139-z (PMC8298205; doi:10.1038/s41562-021-01139-z)
Supplement: Supplementary file 1 — Supplementary Materials and Methods, Supplementary Text, Supplementary Figs. 1–28, Supplementary Tables 1–22 and Supplementary Refs. 1–51. [file 41562_2021_1139_MOESM1_ESM.pdf]

nature portfolio

https://doi.org/10.1038/s41562-021-01139-z

Supplementary information

A global analysis of the impact of COVID-19 stay-at-home restrictions on crime

In the format provided by the authors and unedited

Supplementary Materials for

A global analysis of the impact of COVID-19 stay at home restrictions on crime

A.E. Nivette, R. Zahnow, R. Aguilar, A. Ahven, S. Amram, B. Ariel, M.J. Arosemena Burbano, R. Astolfi, D. Baier, H-M. Bark, J.E.H. Beijers, M. Bergman, G. Breetzke, I.A. Concha-Eastman, S. Curtis-Ham, R. Davenport, C. Diaz, D. Fleitas, M. Gerell, K-H. Jang, J. Kääriäinen, T. Lappi-Seppälä, W-S. Lim, R. Loureiro Revilla, L. Mazerolle, G. Meško, N. Pereda, M.F.T. Peres, R. Poblete-Cazenave, S. Rose, R. Svensson, N. Trajtenberg, T. van der Lippe, J. Veldkamp, C.J. Vilalta Perdomo, M.P. Eisner.

Correspondence to: a.e.nivette@uu.nl

This PDF file includes:

1.1 Supplementary materials and methods
- **1.1 Outcome data and definitions: police-recorded daily crimes**
- **1.2 'Treatment' variable: COVID-19 stay at home restrictions**
- **1.3 Additional COVID-19 policy variables**
  - **1.3.1 Google COVID-19 Community Mobility Reports**

2. Supplementary Text
- **2.1 Descriptive statistics**
- **2.2 Moving average time series plots**
- **2.3 Daily time series plots**
- **2.4 Additional information on ITS analyses**
  - **2.4.1 Lima with and without temperature data**
- **2.5 Additional meta-analytic and meta-regression analyses**
- **2.6 Residential and commercial burglaries**

Supplementary Figures 1 to 28
Supplementary Tables 1 to 22
Supplementary References 1-51

1. Supplementary Materials and Methods

1.1 Outcome data and definitions: police-recorded daily crimes

Daily crime data were collected from 27 cities representing 23 countries from around the globe (Supplementary Table 1).

North America	South America	Europe	Asia	Oceania
Chicago, USA	Cali, Colombia	Amsterdam, Netherlands	Muzaffarpur, India	Auckland, New Zealand
Mexico City, Mexico	Guayaquil, Ecuador	Barcelona, Spain	Seoul, South Korea	Brisbane, Australia
San Francisco, USA	Lima, Peru	Hannover, Germany		
Toronto, Canada	Mendoza, Argentina	Helsinki, Finland		
Vancouver, Canada	Montevideo, Uruguay	Ljubljana, Slovenia		
	Rio de Janeiro, Brazil	London, UK		
	São Paulo, Brazil	Malmö, Sweden		
		Stockholm, Sweden		
		Tallinn, Estonia		
		Tel Aviv, Israel		
		Zurich, Switzerland		

Supplementary Table 1. List of cities included in the analyses by region.

For each city, we sought the number of police-recorded offences for assault, burglary, robbery, theft, motor vehicle theft, and homicide. A description of each data source is provided in Supplementary Table 2.

City	Description of source
Amsterdam, the Netherlands	Crime data up until the most recent date were requested from the Dutch National Police
Auckland, New Zealand	Daily crime counts for offences occurring between January 2018 and August 2020 are publicly available and downloaded from the New Zealand Police (1)
Barcelona, Spain	Crime data up until the most recent date were requested from the Mossos d'Esquadra, Catalonia's police force
Brisbane, Australia	Crime data are publicly available, and were downloaded from Queensland Police Service (2)
Cali, Colombia	Data was gathered from the Municipal Observatory of Security (3).
Chicago, USA	Crime data are publicly available, and were downloaded from the Chicago Data Portal (4)
Guayaquil, Ecuador	Crime data up until the most recent date were requested from the Ecuadorian Attorney-General's office
Hannover, Germany	Crime data were requested from State Criminal Police Office of Lower Saxony, Criminological Research and Statistics unit
Helsinki, Finland	Crime data up until the most recent date were requested from the National Police Board of Finland
Lima, Peru	Crime data from January 2018 to May 2020 was requested from the Peruvian National Police's Observatory of Crime.
Ljubljana, Slovenia	Data requested from and provided by the Slovenian Police - PD Ljubljana.
London, UK	Crime data up until the most recent date were drawn from the Metropolitan Police Service (MPS) Total Notifiable Offence (TNO) data.
Malmo, Sweden	Crime data up to June 2020 was requested from the Swedish National Police
Mendoza, Argentina	Crime data was originally produced by the Province's Public Prosecutors and by the Police. The report was made by the Ministry of Security (5)
Mexico City, Mexico	Crime data are publicly available, and were downloaded from DataCDMX (6)
Montevideo, Uruguay	Crime data up until the most recent date were requested from the Ministry of the Interior of Uruguay
Muzaffarpur, India	Crime data are publicly available, and were downloaded from DataBIHAR (7)

Rio de Janeiro, Brazil	Data was provided under request by Instituto de Segurança Pública (ISP), an agency of the government of the state of Rio de Janeiro.
San Francisco, USA	Crime data are publicly available, and were downloaded from DataSF (8)
Sao Paulo, Brazil	Data was provided by Coordenadoria de Análise e Planejamento, a division of Secretaria de Segurança Pública of the government of the state of São Paulo.
Seoul, South Korea	Crime data based on emergency call reports up until the most recent date were requested from Smart Policing Intelligence Center (Police Science Institute) of Korean National Police Agency.
Stockholm, Sweden	Crime data up to June 2020 was requested from the Swedish National Police
Tallinn, Estonia	Crime data up until the most recent date, obtained from the database (e-File) of the Estonian Ministry of Justice
Tel Aviv-Yafo, Israel	Crime data were requested for the Israel National Police (INP)
Toronto, Canada	Crime data are publicly available, and were downloaded from the Toronto Police Service (9)
Vancouver, Canada	Crime data are publicly available, and were downloaded from the Vancouver Police Department (10)
Zurich, Switzerland	Crime data up until the most recent date were requested from the Zurich Cantonal Police, Criminal police data management, Crime analysis unit

Supplementary Table 2. Description of data sources for each city and country.

Since crime categories and definitions were likely to deviate across countries and cities, we utilized the International Classification of Crime for Statistical Purposes (*11*) to ensure that crime categories were as comparable as possible. ICCS definitions for each crime category are available in Supplementary Table 3.

Importantly, we also sought, where available, police-recorded data on domestic-type assault or family-based crimes. However, due to the significant issues related to the categorization and definition of domestic crimes, variability in recording and data collection practices, and reliability of data across countries, we did not include these in the current analyses.

Crime	ICCS code(s)	Definition: International Classification of Crime for Statistical Purposes*	Page number(s)
Intentional homicide	0101	Unlawful death inflicted upon a person with the intent to cause death or serious injury	33-34
Assault	2011	Intentional or reckless application of physical force inflicted upon the body of a person	37
Robbery	401	Unlawfully taking or obtaining property with the use of force or threat of force against a person with intent to permanently or temporarily withhold it from a person or organization	54
Burglary	501	Gaining unauthorized access to a part of a building/dwelling or other premises with or without the use of force against the building/dwelling, with intent to commit theft or when actually committing theft	57
Theft	502	Unlawfully taking or obtaining of property with the intent to permanently withhold it from a person or organization without consent and without the use of force, threat of force or violence, coercion or deception	58
Theft - motor vehicle	5021	Theft of a motorized vehicle or parts of a motorized of vehicle	58

Supplementary Table 3. International Classification of Crime for Statistical Purposes [ICCS] definitions for key crime categories. Page numbers refer to the English version.

While we strived for the most comparable crime categories for each city, there were nevertheless some deviations across countries. Each local collaborator was asked to provide the local definition for all crime categories, as well as any notable deviations from the definition or remarks about categorization important for comparison. All local definitions and deviations are

presented in Supplementary Tables 4 through 9. In some cases, certain crime categories, such as vehicle theft or burglary, were not distinguished as separate categories by police. In cities where this occurs, the definition is either omitted ('N/A') or the combination is mentioned in the remarks. In addition, where categories were not able to be distinguished, we excluded these cases from further analyses. Information on the time series and cities and respective crime categories included in the analysis is available in Supplementary Table 10.

City	Definition	Remark
Amsterdam (NL)	Intentionally taking another man's life, also includes encouraging one to take his own life.	Incl. encouraging suicide, manslaughter
Auckland (NZL)	Unlawfully killing another person.	
Barcelona (ESP)	Killing another person.	
Brisbane (AUS)	A person who unlawfully kills another or intents to do so.	
Cali (COL)	Unlawful death inflicted upon a person with the intent to cause death or serious injury (ICCS).	
Chicago (USA)	The willful (nonnegligent) killing of one human being by another.	
Guayaquil (ECU)	Killing another person.	
Hannover (GER)	Killing another person.	Incl. negligent killing
Helsinki (FIN)	Intentionally killing someone.	
Lima (PER)	Anyone who kills another intentionally.	Incl. encouraging suicide
Ljubljana (SLO)	Whoever murders another human being by taking his life.	Incl. only murder
London (ENG)	Unlawfully killing another human being.	
Malmö (SWE)	The wilful act of taking someone's life.	
Mendoza (ARG)	Illegal act of a person who intentionally takes the life of another.	
Mexico City (MEX)	Unlawful death inflicted upon a person with the intent to cause death or serious injury (ICCS).	
Montevideo (URU)	Unlawful death inflicted upon a person with the intent to cause death or serious injury (ICCS).	
Muzaffarpur (IND)	Whoever causes death by doing an act with the intention of causing death.	
Rio de Janeiro (BRA)	Unlawful death inflicted upon a person with the intent to cause death or serious injury.	Incl. killings by the police
San Francisco (USA)	The willful (nonnegligent) killing of one human being by another.	
São Paulo (BRA)	Unlawful death inflicted upon a person with the intent to cause death or serious injury.	Incl. killings by the police
Seoul (KOR)	Intentionally killing someone.	
Stockholm (SWE)	The willful act of taking someone's life.	
Tallinn (EST)	Killing someone. Includes both manslaughter and murder.	Incl. attempts
Tel Aviv (ISR)	Unlawful death inflicted upon a person with the intent to cause death or serious injury (ICCS).	
Toronto (CAN)	When a person directly or indirectly, by any means, causes the death of another human being.	
Vancouver (CAN)	A person, directly or indirectly, by any means, causes the death of another person.	
Zurich (SWI)	Deliberately killing another person.	

Supplementary Table 4. Local crime definitions for homicide.

City	Definition	Remark
Amsterdam (NL)	Deliberately injuring someone.	
Auckland (NZL)	The direct (and immediate/confrontational) infliction of force, injury or violence upon a person.	
Barcelona (ESP)	Threatening or assaulting another person (say: I'm going to kill you, punch you, etc.).	
Brisbane (AUS)	Any person who unlawfully assaults another or caused bodily harm.	
Cali (COL)	N/A.	
Chicago (USA)	An unlawful attack by one person upon another that includes severe or bodily injury.	Incl. assault and battery; excl. assaults tagged as 'domestic'
Guayaquil (ECU)	Serious assaults that cause injury, illness, or disability that lasts 4 days or more (lesiones).	
Hannover (GER)	Causing damage to health or bodily harm to another person, including by negligence.	
Helsinki (FIN)	Injures to the health of another, causing pain to another or rendering another unconscious or into a comparable condition.	
Lima (PER)	Anyone who causes serious damage to the body or health of others.	
Ljubljana (SLO)	Whoever inflicts bodily harm on another person or damages his health that the life is in danger.	
London (ENG)	Using force against another person, usually causing physical damage.	
Malmö (SWE)	Anyone who has caused another person bodily injury, illness, pain or powerlessness.	
Mendoza (ARG)	Intentional physical act of offensive contact of one person towards another creating damage.	
Mexico City (MEX)	Intentional application of physical force inflicted upon the body of a person.	
Montevideo (URU)	Someone who, without the intention to kill, causes a person an physical injury.	
Muzaffarpur (IND)	N/A.	
Rio de Janeiro (BRA)	Offend the bodily integrity or health of others.	
San Francisco (USA)	An unlawful attack by one person upon another that includes severe or bodily injury.	Excl. battery of spouse/partner
São Paulo (BRA)	Offend the bodily integrity or health of others.	
Seoul (KOR)	A crime inflicting tangible force on the human body.	
Stockholm (SWE)	Anyone who has caused another person bodily injury, illness, pain or powerlessness.	
Tallinn (EST)	Causing serious health damage by physical abuse.	Incl. attempts, family assault
Tel Aviv (ISR)	Intentional or reckless application of physical force inflicted upon the body of a person (ICCS).	
Toronto (CAN)	The direct or indirect application of force to another person.	
Vancouver (CAN)	"Offense against a person" = Broad category of assault, sexual assault, domestic and robbery.	
Zurich (SWI)	Someone who intentionally damages a person's body or health in any way other than homicide.	Incl. attempts

Supplementary Table 5. Local crime definitions for assault.

City	Definition	Remark
Amsterdam (NL)	Removal or extortion by force or under threat of money or goods.	
Auckland (NZL)	The unlawful taking of property accompanied by the use, and/or threatened use, of force.	
Barcelona (ESP)	Stealing an object using physical violence or intimidation against people.	
Brisbane (AUS)	Any person who steals anything accompanied by violence or the threat of using violence.	
Cali (COL)	N/A.	
Chicago (USA)	The taking of anything of value from the care, custody, or control of a person by force.	
Guayaquil (ECU)	Stealing someone else’s property using violence or threats before the act to facilitate it or during or after the act to seek impunity.	
Hannover (GER)	Whoever, by force against a person or threats of present danger to life or limb, takes movable property belonging to another from another.	
Helsinki (FIN)	Taking property from the possession another person through the use or direct threat of violence.	
Lima (PER)	Anyone who illegitimately seizes a personal property using violence.	
Ljubljana (SLO)	Taking another's movable property with intention of unlawfully appropriating it by force.	
London (ENG)	Theft with the use of force or a threat of force.	Excl. snatch theft
Malmö (SWE)	Stealing through the use of violence or threats of violence.	
Mendoza (ARG)	Dispossession of private property of one person by another using force, violence or threats.	
Mexico City (MEX)	Unlawfully taking or obtaining property with the use of force or threat of force (ICCS).	
Montevideo (URU)	The use of violence or threat of using violence in order to steal.	
Muzaffarpur (IND)	The taking of any movable property out of the possession of any person without consent by using fear of death or hurt, or wrongful restraint.	
Rio de Janeiro (BRA)	Taking someone else's things by means of a serious threat or violence to the person.	
San Francisco (USA)	The taking of anything of value from the care, custody, or control of a person by force.	
São Paulo (BRA)	Taking someone else's things by means of a serious threat or violence to the person.	
Seoul (KOR)	Taking the other’s property by assault or threat.	Incl. robbery & burglary
Stockholm (SWE)	Stealing through the use of violence or threats of violence.	
Tallinn (EST)	Taking away of property of another by use of violence.	Incl. attempts
Tel Aviv (ISR)	Unlawfully taking or obtaining property with the use of force or threat of force (ICCS).	
Toronto (CAN)	The act of taking property from another person or business by the use of force.	
Vancouver (CAN)	N/A.	
Zurich (SWI)	Committing a theft by force against a person or under threat of present danger to life or limb	Incl. attempts

Supplementary Table 6. Local crime definitions for robbery.

City	Definition	Remark
Amsterdam (NL)	Theft by means of breaking and entering in/from a room where one lives or another building.	
Auckland (NZL)	The unlawful entry of a structure with the intent to commit an offence.	
Barcelona (ESP)	Stealing an object by force from an establishment or someone's house.	
Brisbane (AUS)	Any person who enters the dwelling of another with intent to commit an indictable offence.	
Cali (COL)	N/A.	
Chicago (USA)	The unlawful entry of a building to commit a felony/theft.	
Guayaquil (ECU)	N/A.	
Hannover (GER)	Who commits theft for the commission of which they break into or enter private premises.	
Helsinki (FIN)	Breaking into someone's house, shop etc. with the purpose to steal something.	
Lima (PER)	Illegal entry into a building/house with the intent to commit a crime, especially theft.	
Ljubljana (SLO)	Entering any enclosure by way of burgling in order to steal something.	
London (ENG)	Burglary is the theft, or attempted theft, from a premises where access is not authorized.	
Malmö (SWE)	Break into someone's house, shop etc. with the purpose to steal something.	
Mendoza (ARG)	Forcefully entering a private or restricted property/premise and removing elements (i.e. theft).	Incl. cattle theft
Mexico City (MEX)	Gaining unauthorized access to a dwelling with intent to or actually committing theft (ICCS).	
Montevideo (URU)	N/A.	
Muzaffarpur (IND)	Entering into or upon property with intent to commit an offence or to intimidate, insult or annoy any person.	
Rio de Janeiro (BRA)	N/A.	
San Francisco (USA)	The unlawful entry of a building to commit a felony/theft.	
São Paulo (BRA)	N/A.	
Seoul (KOR)	Taking the other's property by assault or threat.	Incl. robbery & burglary
Stockholm (SWE)	Break into someone's house, shop etc. with the purpose to steal something.	
Tallinn (EST)	N/A.	
Tel Aviv (ISR)	Gaining unauthorized access to a dwelling with intent to or actually committing theft (ICCS).	Incl. only residential
Toronto (CAN)	The act of entering a place with the intent to commit an indictable offence therein.	
Vancouver (CAN)	Breaking and entering into a property with the intent to commit an offence.	
Zurich (SWI)	Who takes someone else's things in order to enrich himself or another person unlawfully by forcibly gaining access to a residence.	Incl. attempts

Supplementary Table 7. Local crime definitions for burglary.

City	Definition	Remark
Amsterdam (NL)	Unlawfully taking or obtaining of property.	
Auckland (NZL)	The unlawful taking or obtaining of money or goods without the use of force/violence.	
Barcelona (ESP)	Stealing an object from establishment or person without using violence or force.	
Brisbane (AUS)	A person who fraudulently takes anything capable of being stolen.	
Cali (COL)	N/A.	
Chicago (USA)	The unlawful taking, carrying, or riding away of property from the possession of another.	
Guayaquil (ECU)	Unlawfully taking someone else's property without using violence, threat or intimidation.	Incl. burglary & motor vehicle theft
Hannover (GER)	N/A.	
Helsinki (FIN)	Taking movable property from someone else, with the purpose of acquiring the object.	
Lima (PER)	Whoever, to obtain profit, illegitimately seizes a movable property.	
Ljubljana (SLO)	Taking another's movable property with the intention of unlawfully appropriating it.	
London (ENG)	Theft (i.e. stealing) from a person, motor vehicle, bikes, residential/non-residential property.	
Malmö (SWE)	Takes something from someone else, with the purpose of acquiring the thing.	
Mendoza (ARG)	The permanent unauthorized deprivation of private property.	
Mexico City (MEX)	Unlawfully taking property with intent to permanently withhold it from a person/organization.	
Montevideo (URU)	Stealing when there is no violence included.	Incl. burglary & motor vehicle theft
Muzaffarpur (IND)	Taking dishonestly any movable property out of the possession of any person without that person's consent.	Incl. motor vehicle theft
Rio de Janeiro (BRA)	Take property in benefit of oneself or third parties.	
San Francisco (USA)	The unlawful taking, carrying, or riding away of property from the possession of another.	
São Paulo (BRA)	Take property in benefit of oneself or third parties.	
Seoul (KOR)	Stealing property through acts such as invading a residence.	
Stockholm (SWE)	Takes something from someone else, with the purpose of acquiring the thing.	
Tallinn (EST)	Taking away of movable property of another with the intention of illegal appropriation.	Incl. attempts, burglary, motor vehicle theft
Tel Aviv (ISR)	Unlawfully taking property with intent to permanently withhold it from a person/organization.	
Toronto (CAN)	N/A.	
Vancouver (CAN)	Theft of property that includes personal items (purse, laptop, bicycle, etc.).	
Zurich (SWI)	N/A.	

Supplementary Table 8. Local crime definitions for theft.

City	Definition	Remark
Amsterdam (NL)	Theft of a car/motor vehicle on a public road or a place that is freely accessible to the public.	
Auckland (NZL)	The taking of another person's motor vehicle illegally.	Incl. parts of vehicle theft
Barcelona (ESP)	Stealing a motor vehicle (a car, a motorcycle, a truck, etc.)	
Brisbane (AUS)	A person who unlawfully uses any motor vehicle, aircraft or vessel without consent.	
Cali (COL)	N/A.	
Chicago (USA)	The theft or attempted theft of a motor vehicle.	
Guayaquil (ECU)	N/A.	
Hannover (GER)	Whoever takes movable property belonging to another away from another with the intention of unlawfully appropriating it for themselves or a third party.	
Helsinki (FIN)	Unauthorised use of a motor vehicle of another.	
Lima (PER)	Illegally taking a motor vehicle or its parts or accessories.	
Ljubljana (SLO)	Taking another's property regarding auto/motor vehicles, intention of unlawfully appropriating.	Incl. all vehicles (e.g. bus)
London (ENG)	Theft (i.e. stealing) of motor vehicles.	Incl. only motor thefts
Malmö (SWE)	Unlawfully taking and using a motor vehicle or other motor driven means of transport.	
Mendoza (ARG)	illegitimate deprivation of a motor/auto vehicle from one person by another.	
Mexico City (MEX)	Unlawful seizure of a vehicle.	
Montevideo (URU)	N/A.	
Muzaffarpur (IND)	N/A.	
Rio de Janeiro (BRA)	Taking a motor vehicle in benefit of oneself or third parties.	Incl. only motor thefts
San Francisco (USA)	The theft or attempted theft of a motor vehicle.	
São Paulo (BRA)	Taking a motor vehicle in benefit of oneself or third parties.	Incl. only motor thefts
Seoul (KOR)	N/A.	
Stockholm (SWE)	Unlawfully taking and using a motor vehicle or other motor driven means of transport.	
Tallinn (EST)	N/A.	
Tel Aviv (ISR)	Theft of a motorized vehicle.	Excl. parts of vehicle theft
Toronto (CAN)	The act of taking another person's vehicle.	Incl. only auto thefts
Vancouver (CAN)	Theft of a vehicle, motorcycle, boat or any motor vehicle.	
Zurich (SWI)	N/A.	

Supplementary Table 9. Local crime definitions for vehicle theft.

City	TS start	TS end	Assault	Theft	Burglary	Robbery	Vehicle theft	Homicide
Amsterdam	01/Jan/18	15/May/20	✓	✓	✓	✓	✓	✓
Auckland	01/Jan/18	31/Jul/20	✓	✓	✓	✓	✓	✓
Barcelona	01/Jan/18	15/May/20	✓	✓	✓	✓	✓	✓
Brisbane	01/Jan/19	27/Apr/20	✓	✓	✓	✓	✓	✓
Cali	01/Jan/18	06/Jun/20						✓
Chicago	01/Jan/18	19/May/20	✓	✓	✓	✓	✓	✓
Hannover	01/Jan/19	31/Jul/20	✓		✓	✓	✓	✓
Helsinki	01/Jan/18	30/Jun/20	✓	✓	✓	✓	✓	✓
Lima	01/Jan/18	17/May/20	✓	✓	✓	✓	✓	✓
Ljubljana	01/Jan/19	31/Aug/20	✓	✓	✓	✓	✓	✓
London	01/Jan/20	30/Apr/20	✓	✓	✓	✓	✓	✓
Malmö	01/Jan/18	31/May/20	✓	✓	✓	✓	✓	✓
Mendoza	01/Jan/19	17/May/20	✓	✓	✓	✓	✓	✓
Mexico City	01/Jan/19	31/May/20	✓	✓	✓	✓	✓	✓
Montevideo	01/Jul/19	31/May/20	✓			✓		✓
Muzaffarpur	01/Mar/18	31/Jul/20			✓	✓		✓
Rio de Janeiro	01/Jan/18	30/Jun/20	✓	✓		✓	✓	✓
San Francisco	01/Jan/18	20/Jun/20	✓	✓	✓	✓	✓	✓
São Paulo	01/Jan/18	30/Jun/20	✓	✓		✓	✓	✓
Seoul	01/Jan/18	07/Sep/20	✓					✓
Stockholm	01/Jan/18	31/May/20	✓	✓	✓	✓	✓	✓
Tallinn	01/Jan/18	07/Jul/20	✓			✓		✓
Tel Aviv-Yafo	01/Jan/18	14/Jun/20	✓	✓	✓	✓	✓	✓
Toronto	01/Jan/18	07/Jun/20	✓		✓	✓	✓	✓
Vancouver	01/Jan/18	09/May/20		✓	✓		✓	✓
Zurich	01/Jan/19	31/Jul/20	✓		✓	✓		✓

Supplementary Table 10. Time series [TS] information and crime categories included by city.

1.2 ‘Treatment’ variable: COVID-19 stay at home restrictions

Supplementary Table 11 provides summary information on the COVID-19 responses for each city, with a focus on stay at home restrictions until the second and third quarter of 2020. Supplementary Table 11 also provides information on the start and (where relevant) end date of the stay at home restrictions in each city. In cases where the end of the time series occurs before the end of the restrictions, this column is left blank.

City	SAH start	SAH lifted	First national case	Stay at home and lockdown characteristics	Source
Amsterdam	12/Mar		28/Feb	Advice to stay at home with symptoms; work from home where possible; avoid social gatherings; (eventually) closing restaurants, bars, clubs	(12)
Auckland	23/Mar	13/May	28/Feb	Alert level 3: restrict movement, avoid non-essential travel and activities; work from home; social gatherings restricted; public venues closed	(13)
Barcelona	14/Mar	21/Jun	01/Feb	Declaration of a state of alarm. It allowed the central government to suspend a region's devolved powers. All civil authorities, regional and local police forces and other civil servants are placed under the orders of the central Government: Limit the circulation or presence of people or vehicles at determined times or in determined places, or oblige them to comply with certain requirements. Temporarily requisition all kinds of assets and impose mandatory services. Temporarily take over and occupy industries, factories, workshops, operations or commercial premises of any kind, with the exception of private households, informing the relevant ministry of such actions. Closure of schools, universities, and educational centers. Limit or ration the use of services or the consumption of essential items. Issue the necessary orders to ensure supply for the markets. Closure of churches and prohibition of religious ceremonies.	(14)
Brisbane	27/Mar	29/May	25/Jan	Implementation of restrictions for private residences. Households must have no more than 10 people in them at any time. These restrictions were followed on April 2nd 2020 by home confinement, movement and gathering restrictions in which residents must stay at home except for essential activities (including e.g., exercise, work, obtaining food, attend a wedding/funeral, attend school).	(15)
Cali	25/Mar		07/Mar	Nationwide lockdown. Residents must stay at home except for 'essential' services and care. Transportation is limited.	(16)
Chicago	18/Mar		21/Jan	Prohibition of non-essential travel and activities; people with COVID-19 symptoms should self-isolate; public gatherings restricted; work from home	(17)
Guayaquil	16/Mar	14/Sep	29/Feb	A state of alarm was declared on March 16th 2020, classes at schools and universities were suspended, only businesses that offered medicine, medical supplies or food were allowed to stay open, mandatory lockdown initially until the April 5th but it kept being extended. Only one member per household was allowed to leave to buy medicine or groceries. Mandatory use of masks and social distancing, vehicular circulation was restricted according to the last number of the license plate. Curfew rules kept changing, beginning from 21:00-5:00, on March 18th it was changed to 16:00-5:00, and on March 25th from 14:00-5:00. Those who did not follow the curfew regulations were sanctioned with 1 to 3 years in prison. Flights and massive events were cancelled. Starting April 12th, a new system was created, classifying provinces into "red", "yellow" or "green" according to the number of cases and deaths, with each color having different restrictions. Guayaquil was classified as red until May 20th, and then as yellow. The state of alarm ended on September 13th and the campaign *"Yo me cuido"* ("I take care of myself") began.	(18-20)

Hannover	17/Mar	20/Apr	28/Jan	All events and meetings prohibited, shops, sports facilities, gyms, discos, clubs, school etc. closed.	(21)
Helsinki	16/Mar	01/Jun	30/Jan	State of emergency declared on March 16th, 2020. Teaching in schools and universities only from a distance, work from home, restrictions on public gatherings (max 10 persons), closing down bars, restaurants, museums, theatres, libraries, sport-events etc, visits to elderly care units banned, people over the age of 70 obliged to avoid contacts with other people, restrictions to travel and movement in southern part of Finland (border between the province of Uusimaa and the rest of the country closed for two weeks in March/April), flights to and from the country closed or severely restricted. Restaurants, cafes, and public spaces reopened on June 1st, 2020. State of Emergency lifted on June 15th, 2020.	(22-23)
Lima	15/Mar		07/Mar	Mandatory social isolation in the country for 15 days, but the government kept extending it. Closing of all country borders; restrictions on social gatherings and large gatherings (>300 people); work from home, the prohibition of travelling; closing of bars, restaurants, etc. Since March 18th, 2020, the government declared curfew (20hrs to 5hrs) because people weren't complying with the law. Prohibition of use of private vehicles, except those needed for essential services. General lockdown stopped on June 26th, 2020 in Lima, but curfew is still on (22hrs to 4hrs). Also, the quarantine was localized to some regions, who have kept the general lockdown for longer periods of time.	(24)
Ljubljana	19/Mar	04/May	05/Mar	Stay at home orders were implemented on March 19th, 2020. This includes restrictions on movement and gatherings in public space, except in regards to carrying out essential activities.	(25-26)
London	16/Mar		01/Feb	Advice to stay at home, avoid unnecessary social contact and travel; avoid bars, restaurants, etc.; work from home where possible	(27)
Malmö	16/Mar		01/Feb	Voluntary lockdown; between March and April 2020: large gatherings banned, vulnerable persons and those with cold symptoms asked to stay at home, high schools and universities asked to teach from distance, recommended to refrain from unnecessary travels, restaurant, café, bar mandated to limit crowding, general recommendations to business and associations to limit social interaction. Employers recommended to encourage work from home, public transport to reduce crowding	(28-29)
Mendoza	20/Mar		03/Mar	There are federal and local regulations. They established a full lockdown on March 20th 2020, closing international borders and transit between provinces and municipalities, banned most economic activities except those considered essential, banned meetings and seriously limited the use of public transport and free movement (National Decree 270/20). However, a week prior there were already some limitations. The lockdown was gradually eased particularly in some jurisdictions. In the Mendoza case, on April 27th they allowed the use of public space for recreation but with certain limitations (Provincial Decree 563/20). In mid-May they moved forward easing some restrictions. Up until the end of the time series, travel from outside the province is yet restricted, and there are police and safety controls inside the Mendoza province.	
Mexico City	30/Mar		28/Feb	A number of compulsory measures were published. Measures include: no gatherings with 25+ people are allowed. Public parks will remain closed. Police patrol cars will constantly remind	(30)

				the public to remain home. Likewise, "No essential activities" in the public, private and social sectors are suspended until April 30th, 2020.	
Montevideo	13/Mar	10/Jul	15/Mar	Sanitary emergency was declared. All public events and potential centers of social gathering (e.g. bars, churches, shopping centers, etc.) were shut down, as well as private and public schools. Country's border with Argentina was closed completely and partially with Brazil (due to the land dry border) and flights were suspended. There was an advice to the population to stay in home, work from home whenever possible and avoid traveling. However, government did not enforce a countrywide lockdown or mandatory house confinement. Rather, it trusted citizens to adhere to voluntary social distancing and follow hygiene protocols.	(31-33)
Muzaffarpur	22/Mar	1/Jun	30/Jan	Complete lockdown with immediate effect. This includes severe restrictions on the movement of individuals and the closure of all establishments, with the exception of those providing essential goods and services. All centers, educational institutions, and commercial establishments such as cinema halls, parks, and shopping malls were closed. Restrictions were relaxed from the April 20th, 2020. This allowed the limited movement of people linked to essential services and businesses. Opening of liquor shops and other essential items contributing to state's revenue. Limited public transport and agriculture-based micro, small and medium enterprises were allowed.	(34)
Rio de Janeiro	13/Mar		29/Feb	There was a gradual application of SaH polices from the March 13th to 24th, 2020. On March 13th, the government of the state of Rio de Janeiro established, among other measures: closing of theaters, concert halls and stadiums, and suspends classes in educational institutions (public and private). Commercial activity within the city was restricted by the March 24th, 2020.	(35-36)
San Francisco	11/Mar		21/Jan	Prohibition on non-essential travel and activities; closing of bars, restaurants, gyms; work from home	(17)
São Paulo	16/Mar		29/Feb	There was a gradual application of SaH polices from March 16th to 23rd, 2020. Main events: on March 16th - Emergency situation decree: a) immediate closure of public buildings (libraries, museums, etc) b) home office regulation for municipal civil servants, c) regulation for public transport, d) gradual suspension of classes in public schools as well in private schools, e) suspension of public authorization for public events (alvarás). On March 23rd, decree limiting customer services and commerce, excepting essential services. Classes on public and private schools were suspended on March 23rd, 2020.	(37)
Seoul	22/Mar	17/Apr	20/Jan	Voluntary social distancing. There was no general lockdown of businesses in South Korea, with supermarkets and other retailers remaining open. Schools, universities, cinemas, and gyms were closed soon after the outbreak.	(38-39)
Stockholm	16/Mar		01/Feb	Voluntary lockdown; between March and April 2020: large gatherings banned, vulnerable persons and those with cold symptoms asked to stay at home, high schools and universities asked to teach from distance, recommended to refrain from unnecessary travels, restaurant, café, bar mandated to limit crowding, general recommendations to business and associations to limit social interaction. Employers recommended to encourage work from home, public transport to reduce crowding	(28-29)

Tallinn	29/Mar	18/May	28/Feb	Advice to stay at home, to avoid social gatherings and non-essential travel; public venues (incl. malls) closed, public gatherings restricted; distance learning; work from home where possible; people with COVID-19 diagnosis should self-isolate.	(40)
Tel Aviv	17/Mar	18/Jul	22/Feb	Advice to stay at home, restrict movement (from April 7th to April 10th, 2020, and from the April 27th to 29th), avoid non-essential travel and activities; work from home; social gatherings restricted; closing of restaurants, gyms, bars	(41)
Toronto	17/Mar		26/Jan	Advice to stay at home, avoid non-essential activities and travel; closing bars, restaurants; restrictions for social gatherings	(42)
Vancouver	17/Mar		26/Jan	Advice to stay at home, avoid non-essential activities and travel; closing bars, restaurants; restrictions for social gatherings	(43-*44*)
Zurich	16/Mar	27/Apr	25/Feb	All schools, shops, markets, restaurants, bars and entertainment and leisure facilities such as museums, libraries, cinemas, concert and theatre halls, sports centres, swimming pools and ski resorts will be closed. Similarly, businesses where keeping a distance cannot be maintained, such as hairdressing salons or beauty salons, will be closed. The Federal Council also calls on the population to avoid all unnecessary contact, to keep their distance and to follow the hygiene measures.	(45)

Supplementary Table 11. Overview of stay at home and containment policies for all cities in the sample during the first three quarters of 2020. The timeline includes the day in which stay at home restrictions were first implemented, the day in which restrictions were first lifted, and the day in which the first national COVID-19 case was recorded.

1.3 Additional COVID-19 policy variables

For additional meta-regression analyses, we examined six separate containment policies, an index of economic support, and the overall stringency index. More detailed information on containment and other policies is available at the OxGRT website (*46-47*).

School closures. The school closure variable is measured using an ordinal scale ranging from 0 to 3. Countries with no measures regarding school closures are coded as 0, recommendations to close are coded as 1, requirements to close only some types of schools (e.g. high schools) are coded as 2, and requirements to close all schools are coded as 3 (*46*).

Workplace restrictions. Workplace restrictions refer to rules that regulate working from home and the closure of workplaces. The variable is measured using an ordinal scale ranging from 0-3, where 0 reflects countries with no measures regarding workplace closures, 1 reflects recommendations to close or work from home, 2 represents requirements to close or work from home for some sectors, and 3 represents requirements to close or work from home for all sectors, with the exception of "essential" workers (*46*).

Restrictions on public events. Restrictions on public events were measured using an ordinal scale, ranging from 0 for no measures, 1 for recommendations to cancel, and 2 for requirements to cancel public events (*46*).

Restrictions on private gatherings. Restrictions on private gatherings include limitations on the number of people that are allowed to gather in a single place. The variable is measured from 0 to 4, where 0 represents no restrictions, 1 represents restrictions on gatherings above 1000 people, 2 reflects restrictions on gatherings of 101-1000 people, 3 reflects restrictions on gatherings of 11-100 people, and 4 reflects restrictions on gatherings of 10 or fewer people (*46*).

Restrictions on internal movement. This variable is measured using an ordinal scale ranging from 0-2, where 0 represents no measures, 1 represents recommendations not to travel between cities or regions, and 2 represents restrictions on internal movement (*46*).

Economic policy index. In response to the impact that COVID-19 policies had on employment and income, many countries implemented economic policies that aimed to relieve negative consequences of these actions. This includes direct income support for affected households, freezing financial obligations or debt relief, stimulus spending, and aid spending to other countries (*46*). The first two variables are measured on ordinal scales ranging from 0 to 2. For income support, 0 reflects no support, 1 reflects replacement of less than 50% of salaries, and 2 reflects replacement of 50% or more of salaries. For financial/debt relief, 0 reflects no relief, 1 reflects narrow or specific relief, and 2 reflects broad relief. Fiscal/stimulus and aid variables are measured in monetary (USD) value.

Overall stringency index. The overall stringency index can range from 0 to 100, whereby higher scores indicate more intense and restrictive policies. While the stringency of stay at home restrictions and the overall stringency index are highly correlated ($r=0.84$, $p<.001$, Supplementary Table 14), the overall stringency index captures both the stringency and breadth of broader policy responses.

Both economic and containment policies (including international travel restrictions) were standardized and combined into the economic and stringency index, respectively. The process for creating the indices is described in detail on the OxCGRT website (*47*).

For the meta-regression analyses, we took the average value of the overall stringency and economic indices, as well as each containment policy score for the period following the implementation of stay at home restrictions, or until the end of the time series (Supplementary

Table 12). Due to the small number of effect sizes included in each model and possible issues with multicollinearity, we estimated the effects of each policy variable separately.

Variable	N	Mean	SD	Min	Max
Stringency of stay at home restrictions	27	1.65	0.69	0.00	2.84
Overall stringency index	27	75.13	13.34	42.45	95.73
Closing schools	27	2.74	0.57	0.97	3.00
Workplace restrictions	27	2.42	0.62	0.88	3.00
Restrictions on public events	27	1.91	0.32	0.79	2.00
Restrictions on private gatherings	27	3.33	1.04	0.00	4.00
Closing public transport	27	0.85	0.76	0.00	2.00
Restrictions on internal movement	27	1.65	0.44	0.75	2.00
Economic support index	27	61.22	18.93	0.00	92.22

Supplementary Table 12. Descriptive statistics for containment policies and overall stringency and economic indices. SD=standard deviation.

1.3.1 Google COVID-19 Community Mobility Reports

The Google COVID-19 Community Mobility Reports provide measures of change in mobility relative to a baseline value for 5 weeks during the period January 5th-February 6th 2020 (*48*). The baseline is measured as the median value for a given day of the week during this period. Changes therefore reflect the percentage difference in visits and length of stays on a given day compared to the baseline value. Data are aggregated and anonymized, and inclusion depends on user settings and privacy thresholds (*49*). The Google mobility reports include measures of change corresponding to a number of public and private places, including retail and recreation (e.g. restaurants, cafes, shopping centers, movie theaters), grocery and pharmacy (e.g. grocery and food shops, drug stores, pharmacies), parks (e.g. local and national parks, dog parks, plazas), workplaces, transit stations (e.g. subway, bus, train stations), and residential places. For the meta-regression analyses, we took the average value of changes in mobility for the period following the implementation of stay at home restrictions, or until the end of the time series. Descriptive statistics for each mobility measure are available in Supplementary Table 13.

Variable	N	Mean	SD	Min	Max
Retail and recreation	27	-57.52	17.69	-87.05	-17.48
Grocery and pharmacy	27	-23.19	15.16	-57.39	3.93
Parks	27	-15.26	46.69	-82.44	68.61
Transit stations	27	-58.15	13.83	-81.04	-13.81
Workplace	27	-46.64	13.12	-67.90	-8.78
Residential	27	20.48	6.19	7.30	30.37

Supplementary Table 13. Descriptive statistics for Google COVID-19 Community Mobility Report measures. Mobility measures reflect percentage change in visits and length of stay in a given location relative to a baseline established in January-February 2020. SD=standard deviation.

It is important to note that the categorization of each place can differ across regions and countries, and Google advise against using the data for comparison across countries. We therefore treat these analyses with caution, and use them only as a robustness check for the stringency index results. However, there is some indication that the Google mobility data do tap into changes in public behavior following the implementation of stay at home restrictions. Bivariate correlations between mobility measures and stringency measures show moderate to strong associations

between the stringency of stay at home restrictions and the percentage change in visits to retail and recreation (*r*=-0.48, p=.01), groceries and pharmacies (*r*=-0.46, p=.02), parks (*r*=-0.52, p=.01), and residential locations (*r*=0.45, p=.02) (Supplementary Table 14). In short, more stringent stay at home restrictions are associated with larger declines in visits to commercial locations and parks, and increases in users remaining in their homes/residences relative to baseline values.

			1	2	3	4	5	6	7
1	Stringency of stay at home restrictions	r	1.00						
		p-value							
2	Overall stringency index	r	0.84	1.00					
		p-value	<0.001						
3	Closing schools	r	0.68	0.76	1.00				
		p-value	<0.001	<0.001					
4	Workplace restrictions	r	0.56	0.71	0.74	1.00			
		p-value	0.002	<0.001	<0.001				
5	Restrictions on public events	r	0.68	0.71	0.89	0.72	1.00		
		p-value	<0.001	<0.001	<0.001	<0.001			
6	Restrictions on private gatherings	r	0.37	0.50	0.38	0.57	0.14	1.00	
		p-value	0.06	0.01	0.05	0.002	0.49		
7	Closing public transport	r	0.49	0.65	0.40	0.29	0.32	0.21	1.00
		p-value	0.01	<0.001	0.04	0.15	0.10	0.29	
8	Restrictions on internal movement	r	0.66	0.63	0.61	0.52	0.58	0.46	0.35
		p-value	<0.001	<0.001	<0.001	0.01	0.002	0.02	0.07
9	Economic support index	r	0.05	0.11	-0.03	-0.18	-0.04	0.15	0.11
		p-value	0.80	0.58	0.88	0.36	0.84	0.44	0.60
10	Retail and recreation (% change)	r	-0.48	-0.65	-0.57	-0.53	-0.53	-0.28	-0.59
		p-value	0.01	<0.001	0.002	0.004	0.004	0.15	0.001
11	Grocery and pharmacy (% change)	r	-0.46	-0.61	-0.39	-0.3	-0.34	-0.18	-0.55
		p-value	0.02	<0.001	0.04	0.13	0.08	0.36	0.003
12	Parks (% change)	r	-0.52	-0.58	-0.47	-0.28	-0.41	-0.10	-0.7
		p-value	0.01	0.002	0.01	0.15	0.03	0.62	<0.001
13	Transit stations (% change)	r	-0.30	-0.42	-0.29	-0.39	-0.32	-0.22	-0.42
		p-value	0.13	0.03	0.14	0.05	0.10	0.27	0.03
14	Workplace (% change)	r	-0.21	-0.32	-0.31	-0.47	-0.32	-0.25	-0.24
		p-value	0.29	0.11	0.11	0.01	0.10	0.21	0.23
15	Residential (% change)	r	0.45	0.57	0.47	0.47	0.42	0.27	0.48
		p-value	0.02	0.002	0.01	0.01	0.03	0.17	0.01

Supplementary Table 14. Bivariate correlations between stringency index measures (variables 1 through 9) and Google COVID-19 mobility measures (variables 10 through 15). Mobility measures reflect percentage change in visits and length of stay in a given location relative to a baseline established in January-February 2020. SD=standard deviation.

			8	9	10	11	12	13	14	15
8	Restrictions on internal movement	r	1.00							
		p-value								
9	Economic support index	r	-0.05	1.00						
		p-value	0.81							
10	Retail and recreation (% change)	r	-0.17	-0.22	1.00					
		p-value	0.39	0.27						
11	Grocery and pharmacy (% change)	r	-0.18	-0.27	0.75	1.00				
		p-value	0.38	0.17	<0.001					
12	Parks (% change)	r	-0.36	-0.05	0.62	0.7	1.00			
		p-value	0.07	0.79	<0.001	<0.001				
13	Transit stations (% change)	r	-0.12	-0.33	0.78	0.71	0.56	1.00		
		p-value	0.56	0.09	<0.001	<0.001	0.002			
14	Workplace (% change)	r	-0.07	-0.27	0.78	0.64	0.46	0.9	1.00	
		p-value	0.72	0.18	<0.001	<0.001	0.02	<0.001		
15	Residential (% change)	r	0.26	0.28	-0.86	-0.76	-0.72	-0.86	-0.88	1.00
		p-value	0.19	0.16	<0.001	<0.001	<0.001	<0.001	<0.001	

Supplementary Table 14 - continued. Bivariate correlations between stringency index measures (variables 1 through 9) and Google COVID-19 mobility measures (variables 10 through 15). Mobility measures reflect percentage change in visits and length of stay in a given location relative to a baseline established in January-February 2020. SD=standard deviation.

2. Supplementary Text

2.1 Descriptive statistics

Descriptive statistics for each city, including population and average daily number of crimes before and after stay at home restrictions, are presented in Supplementary Table 15.

2.2 Moving average time series plots

In order to be able to compare the changes in crime trends following the implementation of stay at home restrictions, we calculated the 7-day moving average for each city and crime type. The trends were indexed to equal 100 at the day in which restrictions were implemented. Figure 1 in the main text presents all cities and crime types in a single graph, with the average trend highlighted in blue. Here we present figures that highlight each city's trend for all available crime types (Supplementary Figures 1-6).

2.3 Daily time series plots

The daily number of assaults, robberies, burglaries, vehicle thefts and homicides are presented in Supplementary Figures 7 through 25.

City	Population (2020)	Assault		Theft		Burglary		Robbery		Vehicle theft		Homicide	
		Mean (Pre)	Mean (Post)	Mean (Pre)	Mean (Post)	Mean (Pre)	Mean (Post)	Mean (Pre)	Mean (Post)	Mean (Pre)	Mean (Post)	Mean (Pre)	Mean (Post)
Amsterdam	1,149,000	11.7	10.9	40.0	25.8	13.6	8.7	4.3	2.6	5.5	4.8	0.8	0.8
Auckland	1,607,000	35.8	35.0	52.9	18.0	55.5	35.6	2.4	0.8	52.9	18.0	0.0	0.0
Barcelona	5,586,000	34.6	14.6	385.2	38.1	31.2	9.3	39.5	8.6	12.9	2.0	0.2	0.2
Brisbane	2,406,000	9.5	6.3	96.0	58.3	29.5	20.6	1.8	1.1	10.3	8.5	0.0	0.0
Cali	2,782,000	--	--	--	--	--	--	--	--	--	--	3.1	2.0
Chicago	8,865,000	189.8	136.0	171.7	84.0	28.5	17.3	23.9	15.1	25.6	21.3	1.5	1.6
Guayaquil	2,698,077	0.6	0.4	--	--	--	--	53.1	31.9	--	--	2.6	3.1
Hannover	1,157,115	28.5	20.8	--	--	7.5	4.4	2.3	2.0	1.4	0.6	0.1	0.2
Helsinki	1,305,000	14.4	9.5	68.1	56.6	12.5	12.2	1.1	0.9	1.1	0.9	0.0	0.0
Lima	10,719,000	30.2	8.6	94.4	15.5	5.3	0.9	141.4	19.0	26.4	6.7	0.8	0.5
Ljubljana	286,745	0.0	0.0	18.7	8.7	5.6	3.8	0.3	0.1	0.3	0.2	0.0	0.0
London	9,304,000	562.6	493.5	727.7	332.3	218.8	127.8	93.4	43.0	84.1	58.3	0.4	0.3
Malmö	322,000	9.4	7.7	54.6	45.7	7.7	7.5	1.5	1.5	1.7	1.1	0.1	0.0
Mendoza	1,173,000	48.8	22.9	92.1	28.2	5.6	2.4	21.4	6.8	13.9	4.9	0.3	0.2
Mexico City	8,738,914	18.7	10.4	185.2	94.2	97.6	41.6	149.5	85.2	33.9	21.1	5.1	5.1
Montevideo	1,752,000	12.7	9.5	--	--	--	--	69.1	57.6	--	--	0.7	0.7
Muzaffarpur	5,418,433	--	--	--	--	1.5	1.0	0.8	0.3	--	--	0.5	0.6
Rio de Janeiro	13,458,000	37.2	14.5	--	--	--	--	303.7	121.6	18.8	13.6	5.3	4.0
San Francisco	3,314,000	24.3	15.9	133.0	60.6	17.9	25.2	9.7	6.1	14.7	17.9	0.1	0.0
São Paulo	22,043,000	103.9	75.8			--	--	210.6	77.3	106.0	36.4	3.2	3.2
Seoul	9,963,000	325.8	289.2	--	--	--	--	--	--	--	--	0.4	0.1
Stockholm	974,073	26.3	22.1	166.0	128.7	22.7	22.1	4.6	3.5	5.8	6.6	0.1	0.2
Tallinn	445,000	6.4	4.9	--	--	--	--	0.2	0.2	- -	- -	0.0	0.0
Tel Aviv-Yafo	463,508	10.6	7.6	24.7	13.7	5.1	3.3	0.8	0.5	2.6	1.8	0.0	0.1
Toronto	6,197,000	52.1	31.8	--	--	21.7	17.7	9.5	5.0	13.4	12.4	0.2	0.2
Vancouver	2,581,000	--	--	64.1	38.6	12.8	17.2	--	--	3.4	2.2	0.0	0.0
Zurich	1,539,275	4.7	3.0	--	--	13.8	14.6	1.3	0.8	--	--	0.0	0.0

Supplementary Table 15. Mean number of crimes per day pre- and post-implementation of stay at home restrictions.

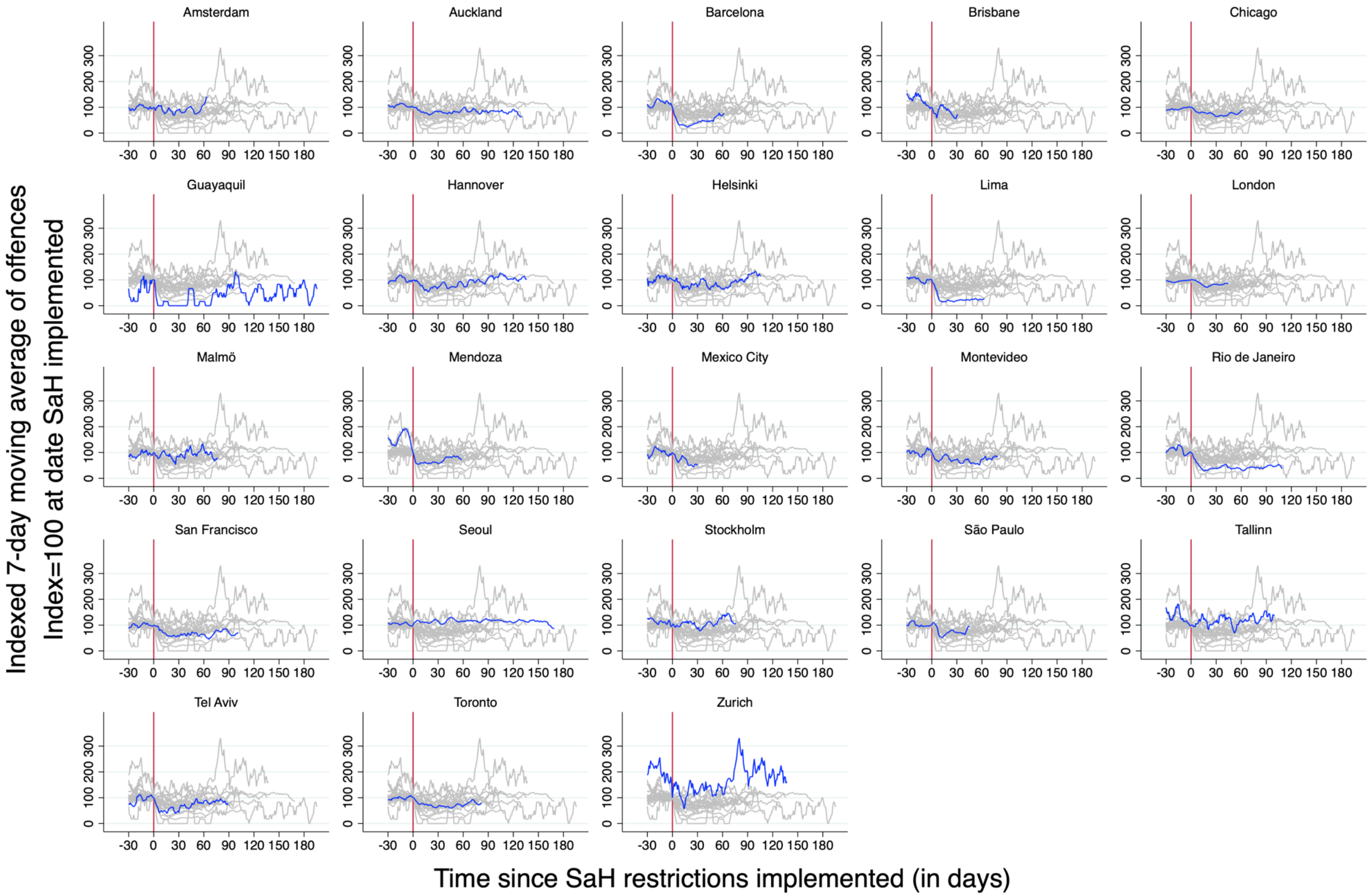

Supplementary Figure 1. Seven-day moving average time series plots of daily number of assaults. Each time series is indexed at 100 at the day the first stay at home restrictions were implemented. The blue line indicates the trend for the specified city.

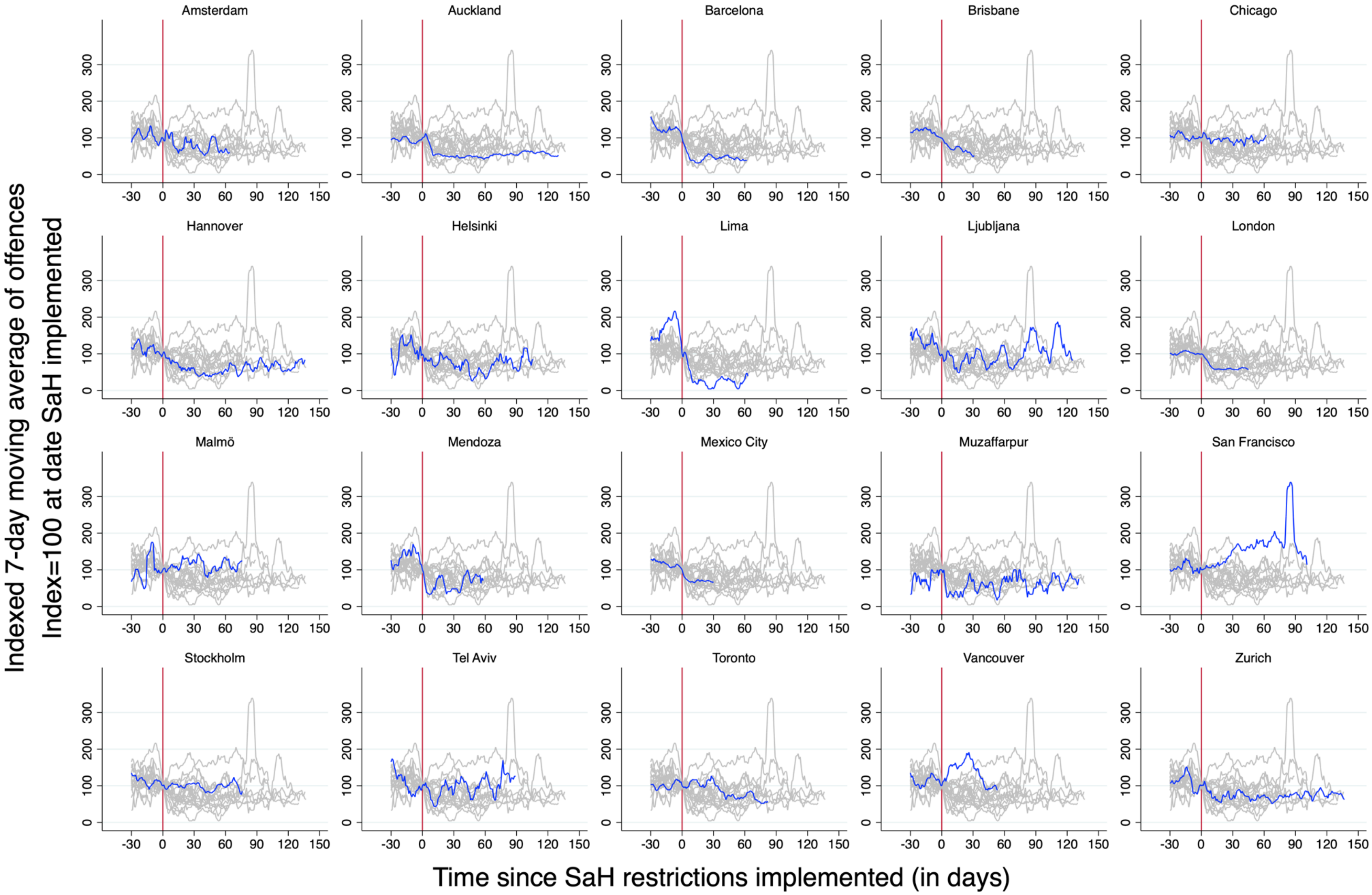

Supplementary Figure 2. Seven-day moving average time series plots of daily number of burglaries. Each time series is indexed at 100 at the day the first stay at home restrictions were implemented. The blue line indicates the trend for the specified city.

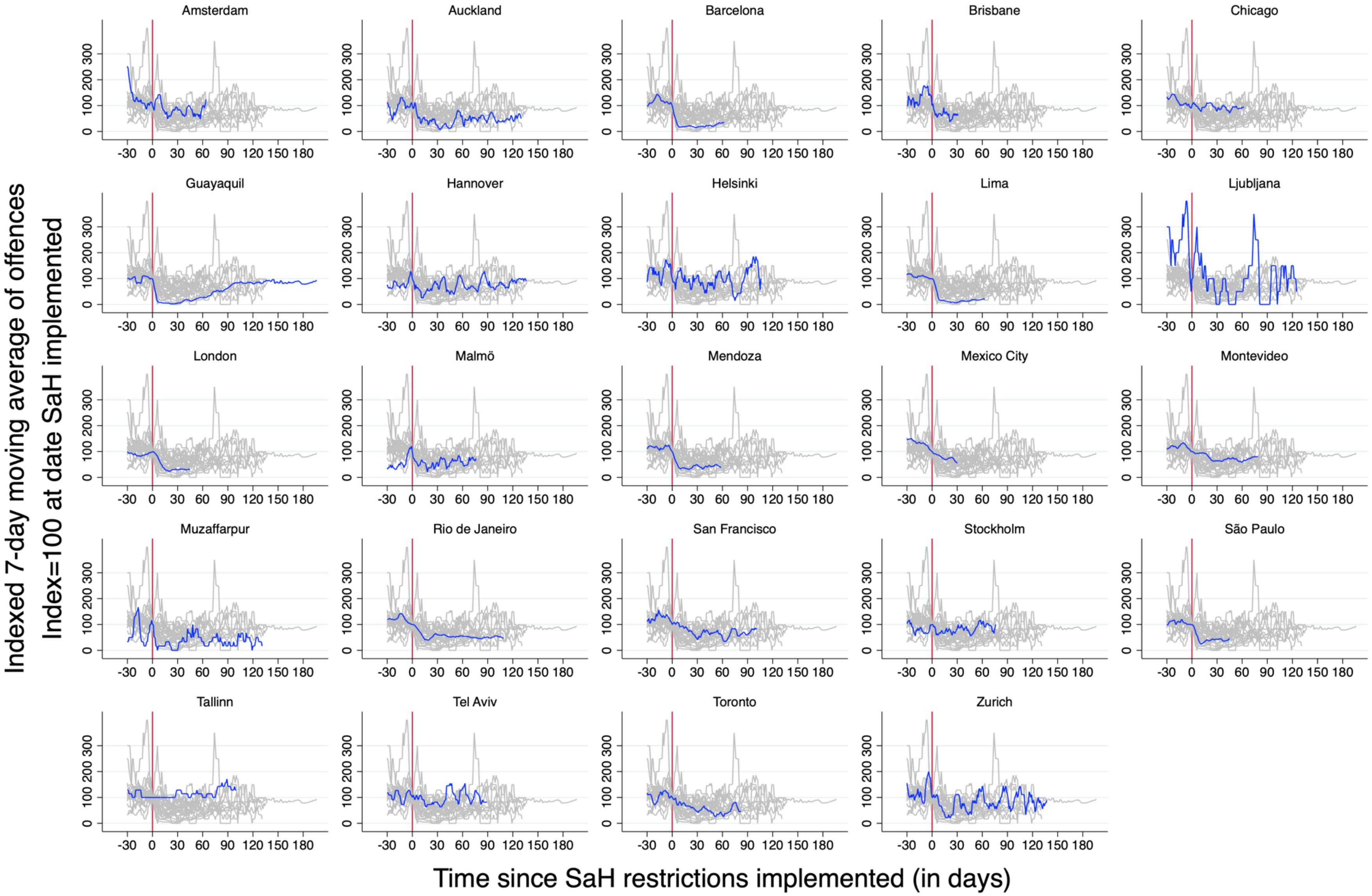

Supplementary Figure 3. Seven-day moving average time series plots of daily number of robberies. Each time series is indexed at 100 at the day the first stay at home restrictions were implemented. The blue line indicates the trend for the specified city.

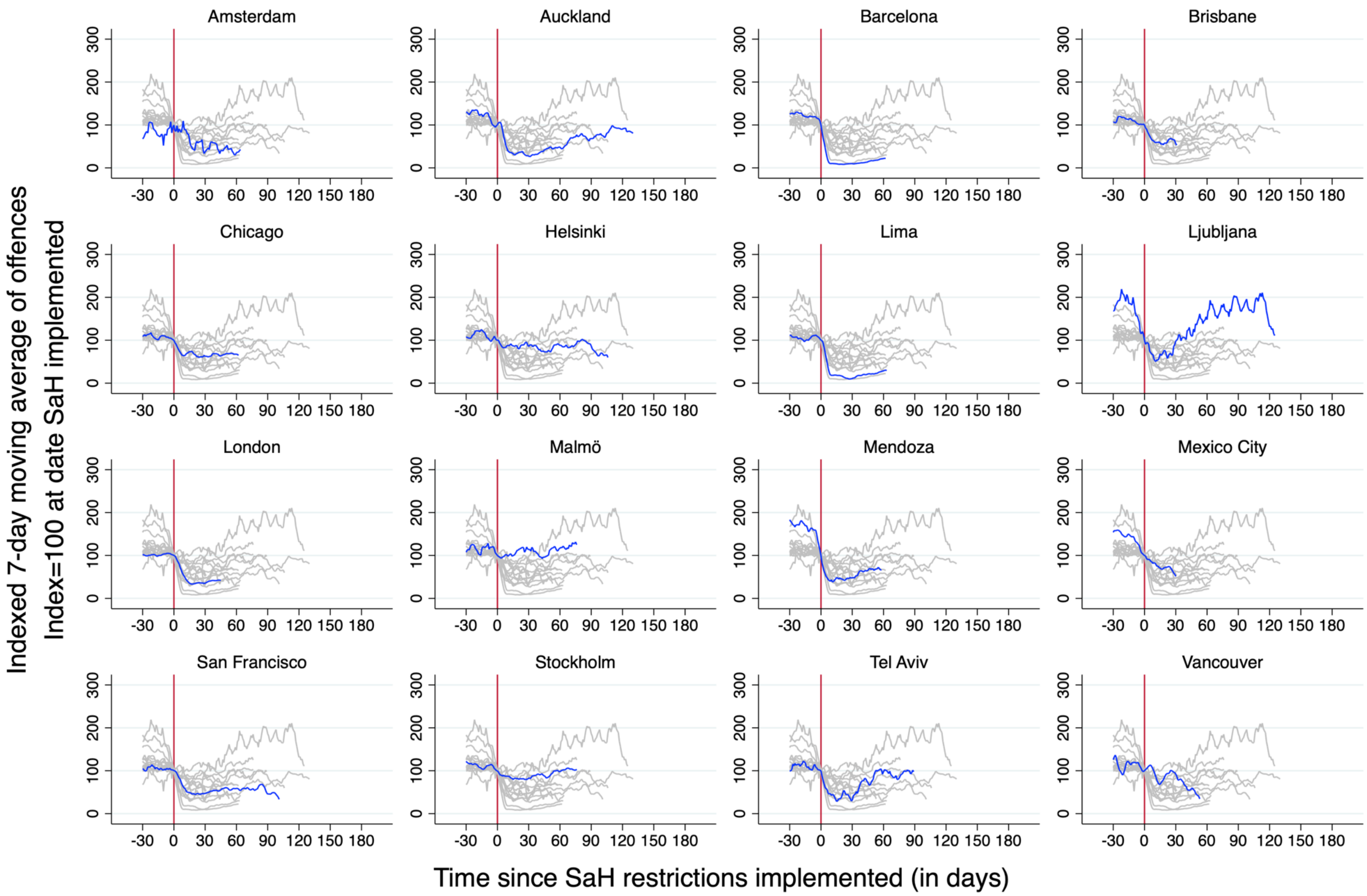

Supplementary Figure 4. Seven-day moving average time series plots of daily number of thefts. Each time series is indexed at 100 at the day the first stay at home restrictions were implemented. The blue line indicates the trend for the specified city.

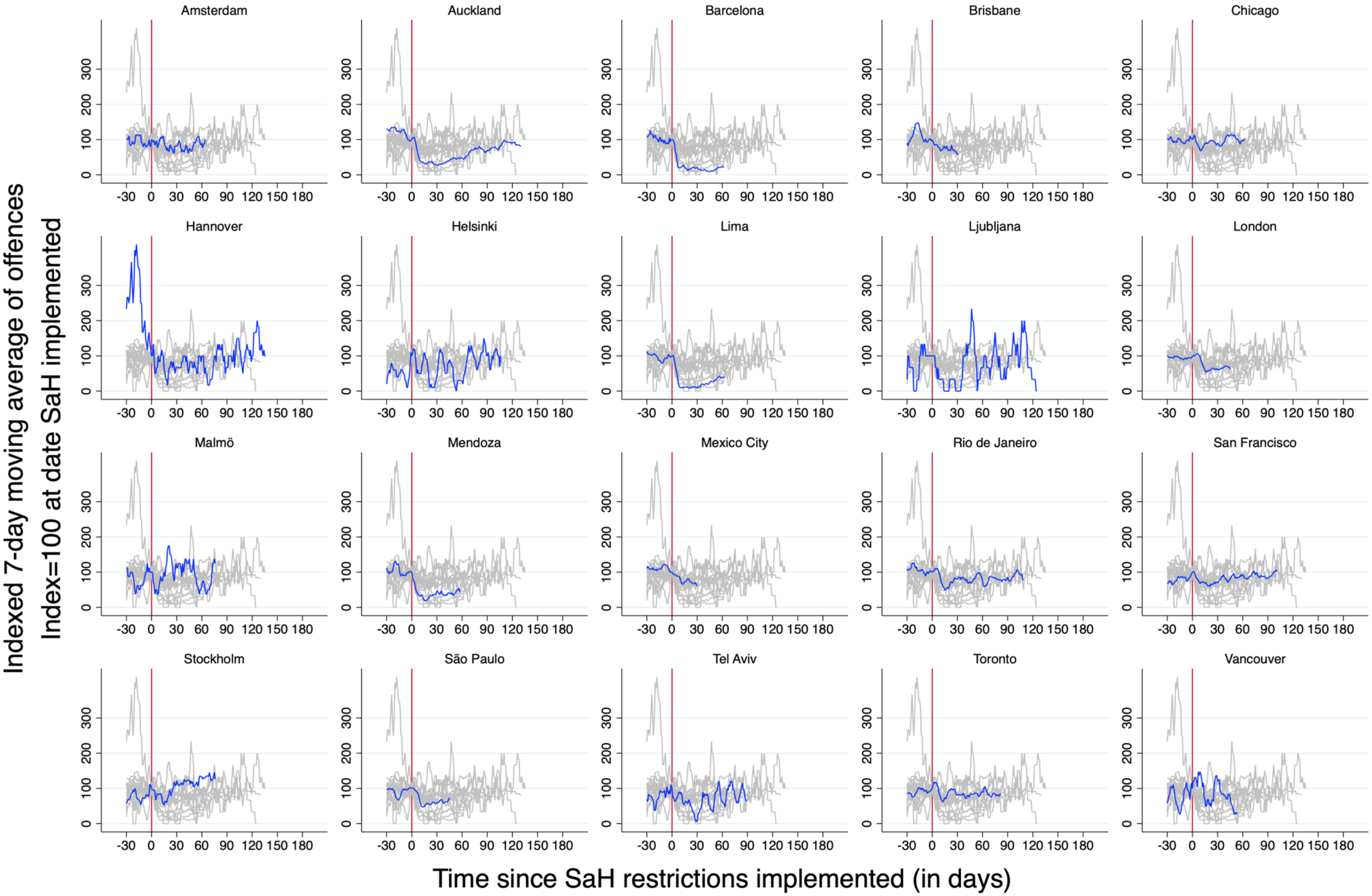

Supplementary Figure 5. Seven-day moving average time series plots of daily number of vehicle thefts. Each time series is indexed at 100 at the day the first stay at home restrictions were implemented. The blue line indicates the trend for the specified city.

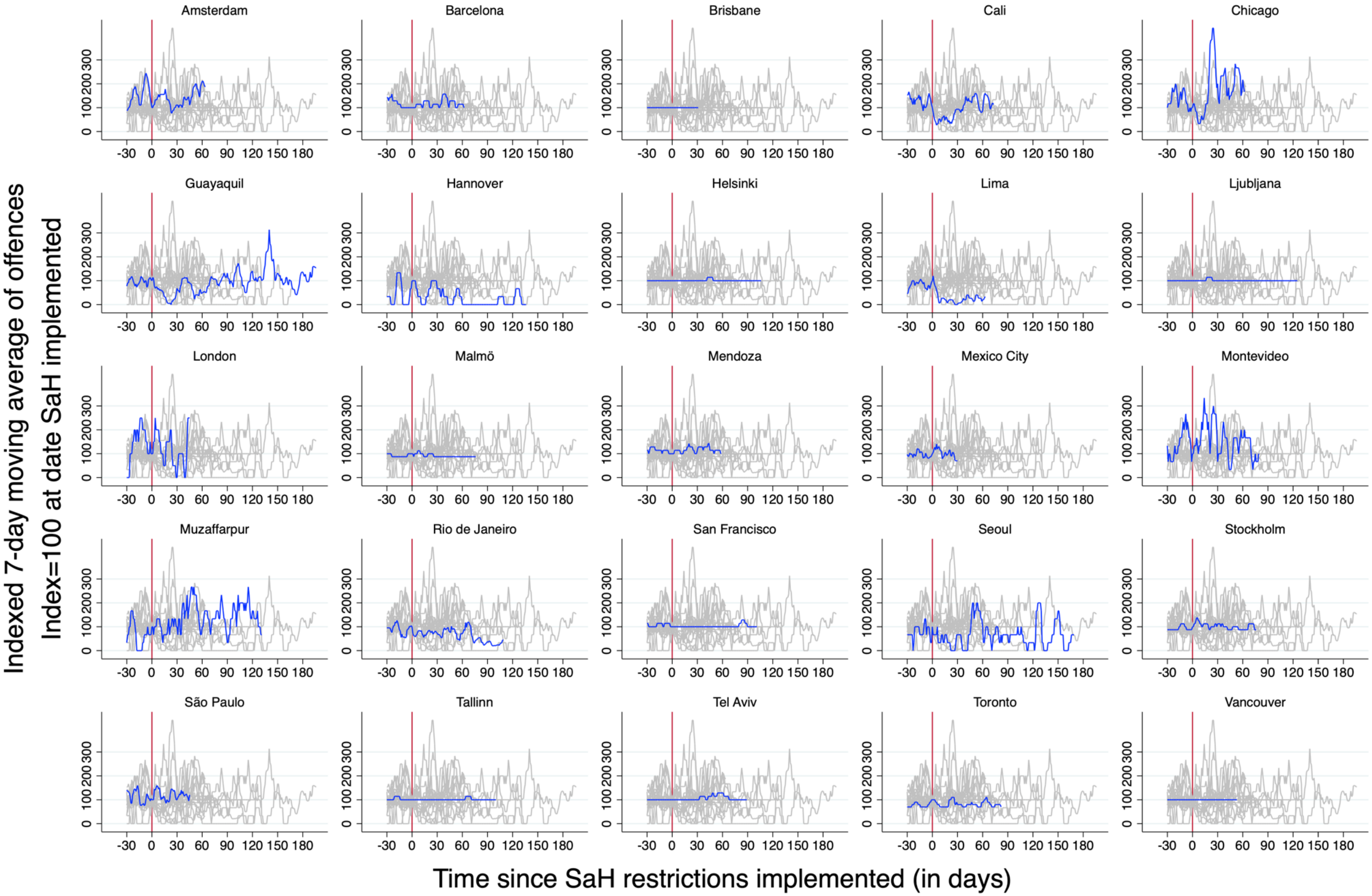

Supplementary Figure 6. Seven-day moving average time series plots of daily number of homicides. Each time series is indexed at 100 at the day the first stay at home restrictions were implemented. The blue line indicates the trend for the specified city.

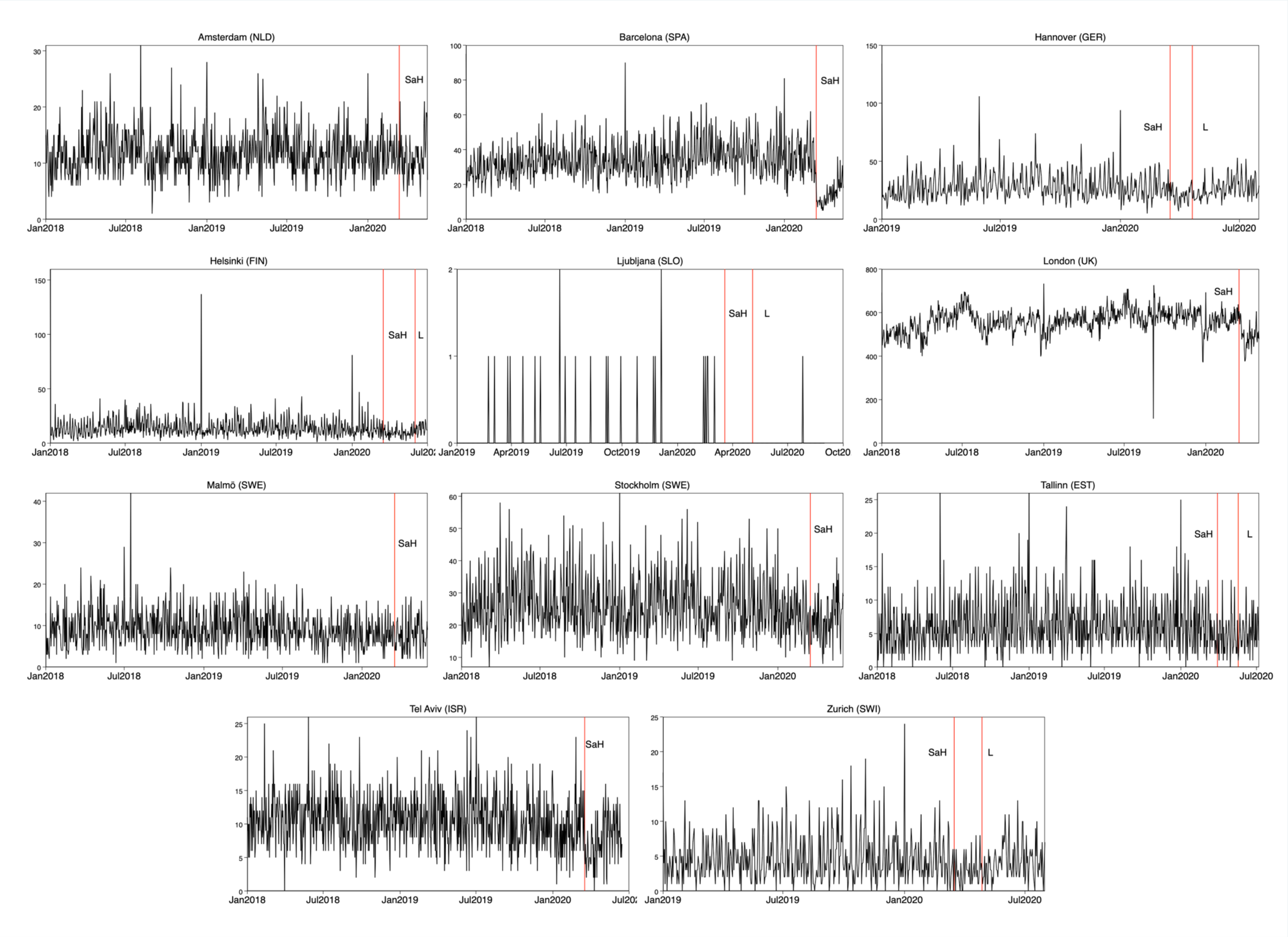

Supplementary Figure 7. Time series plots of daily number of assaults by cities in Europe. SaH = Stay at home restrictions implemented. L = Stay at home restrictions lifted.

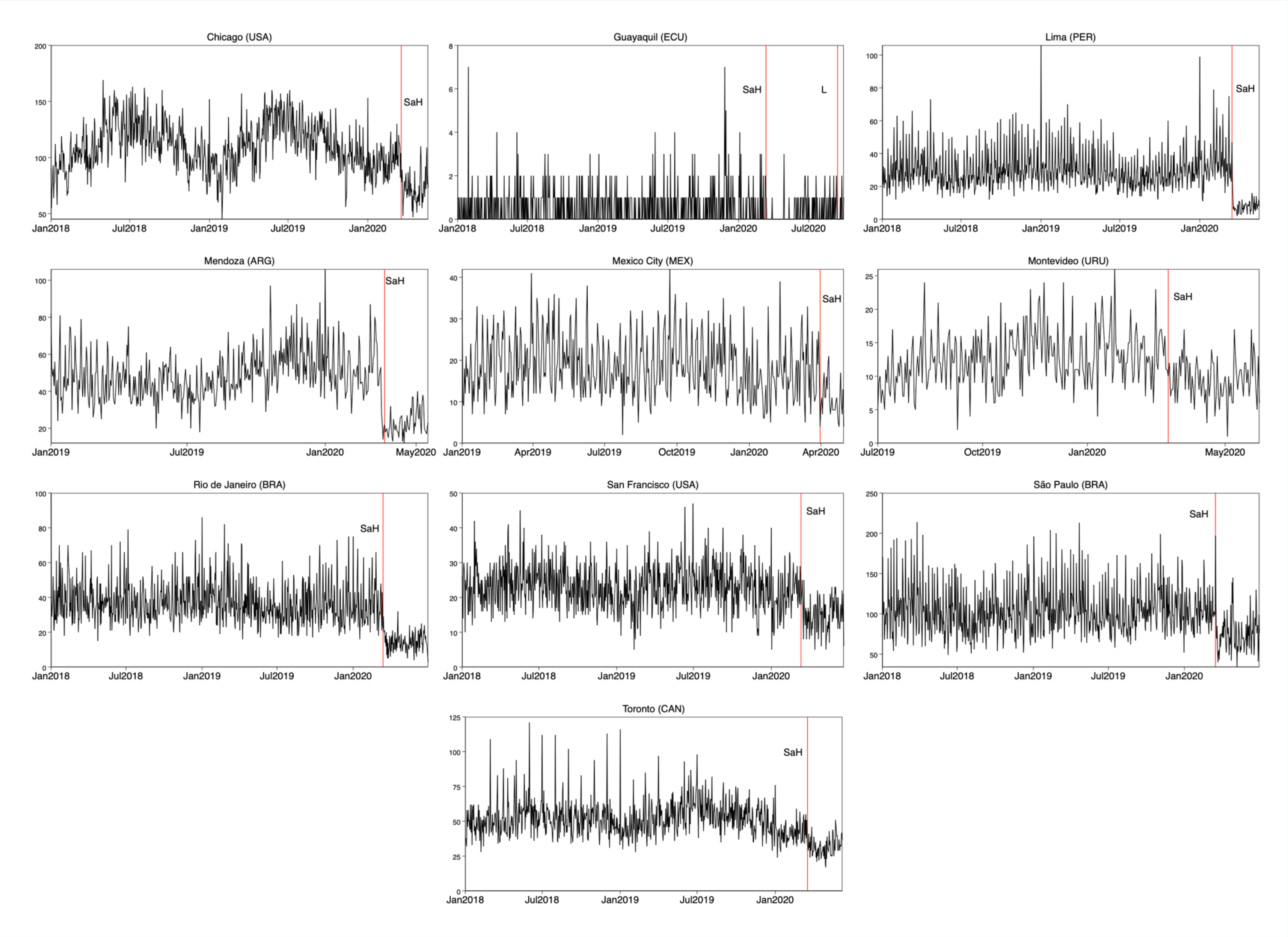

Supplementary Figure 8. Time series plots of daily number of assaults by cities in the Americas. SaH = Stay at home restrictions implemented. L = Stay at home restrictions lifted.

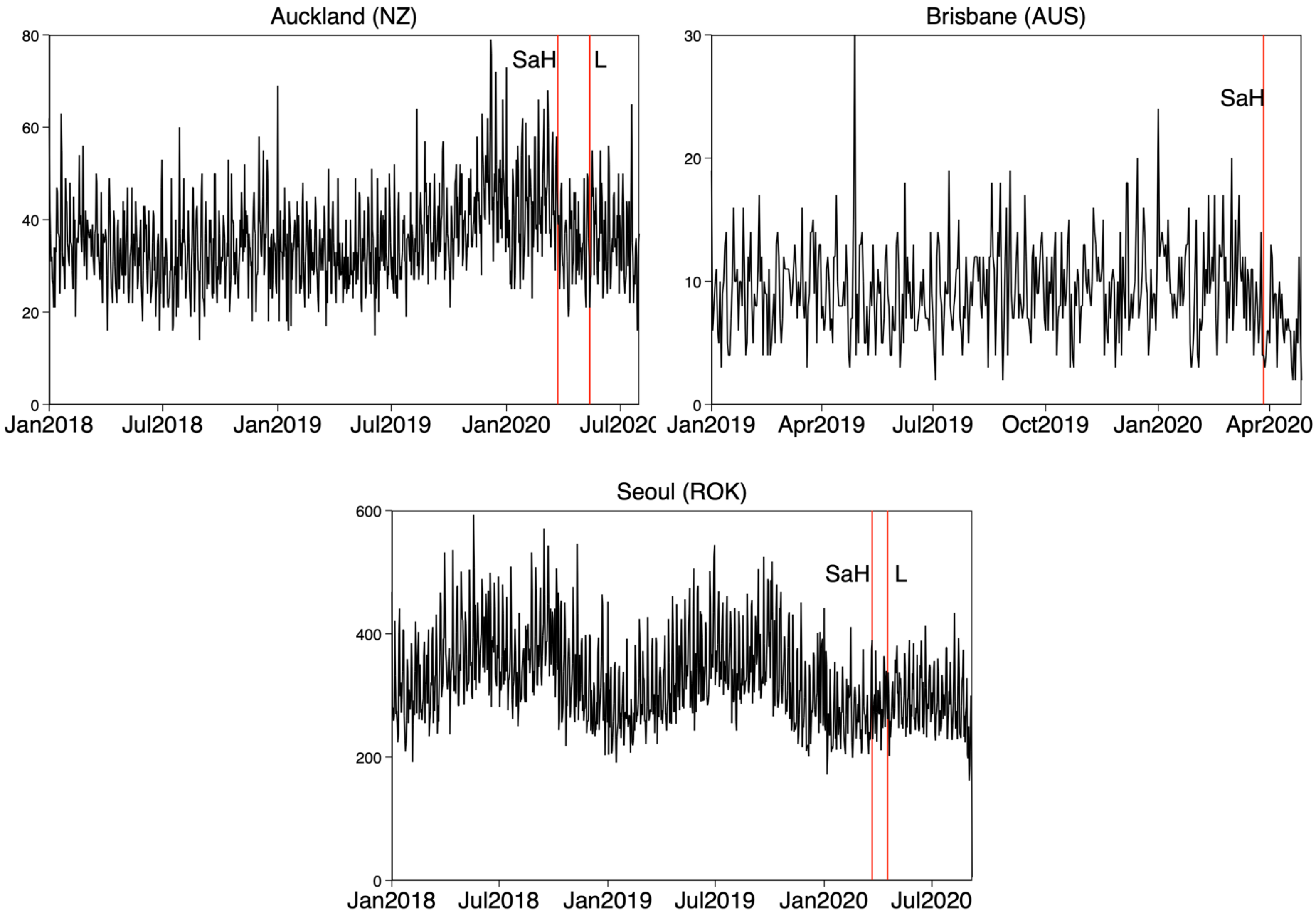

Supplementary Figure 9. Time series plots of daily number of assaults by cities in Asia and Oceania. SaH = Stay at home restrictions implemented. L = Stay at home restrictions lifted.

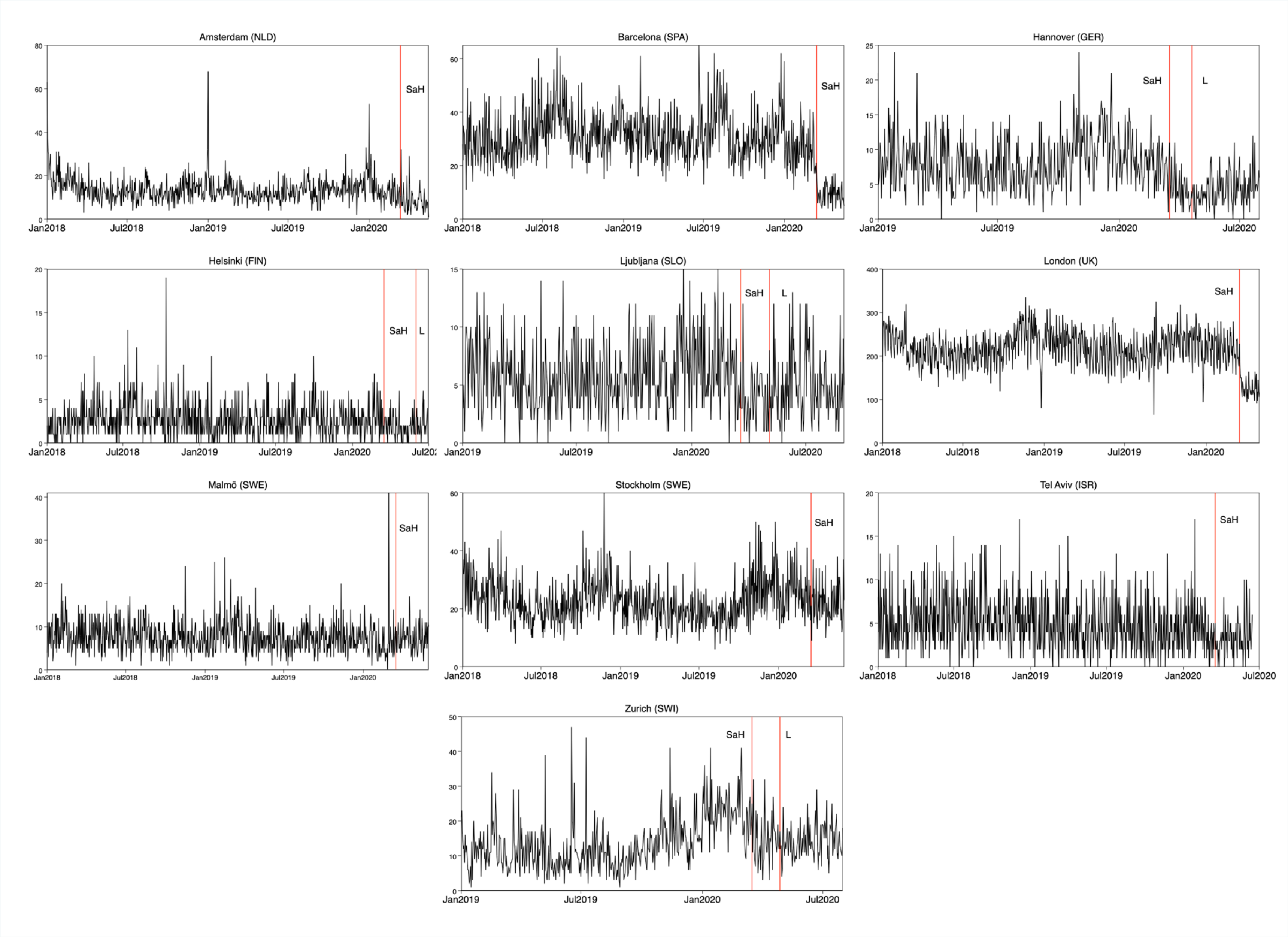

Supplementary Figure 10. Time series plots of daily number of burglaries by cities in Europe. SaH = Stay at home restrictions implemented. L = Stay at home restrictions lifted.

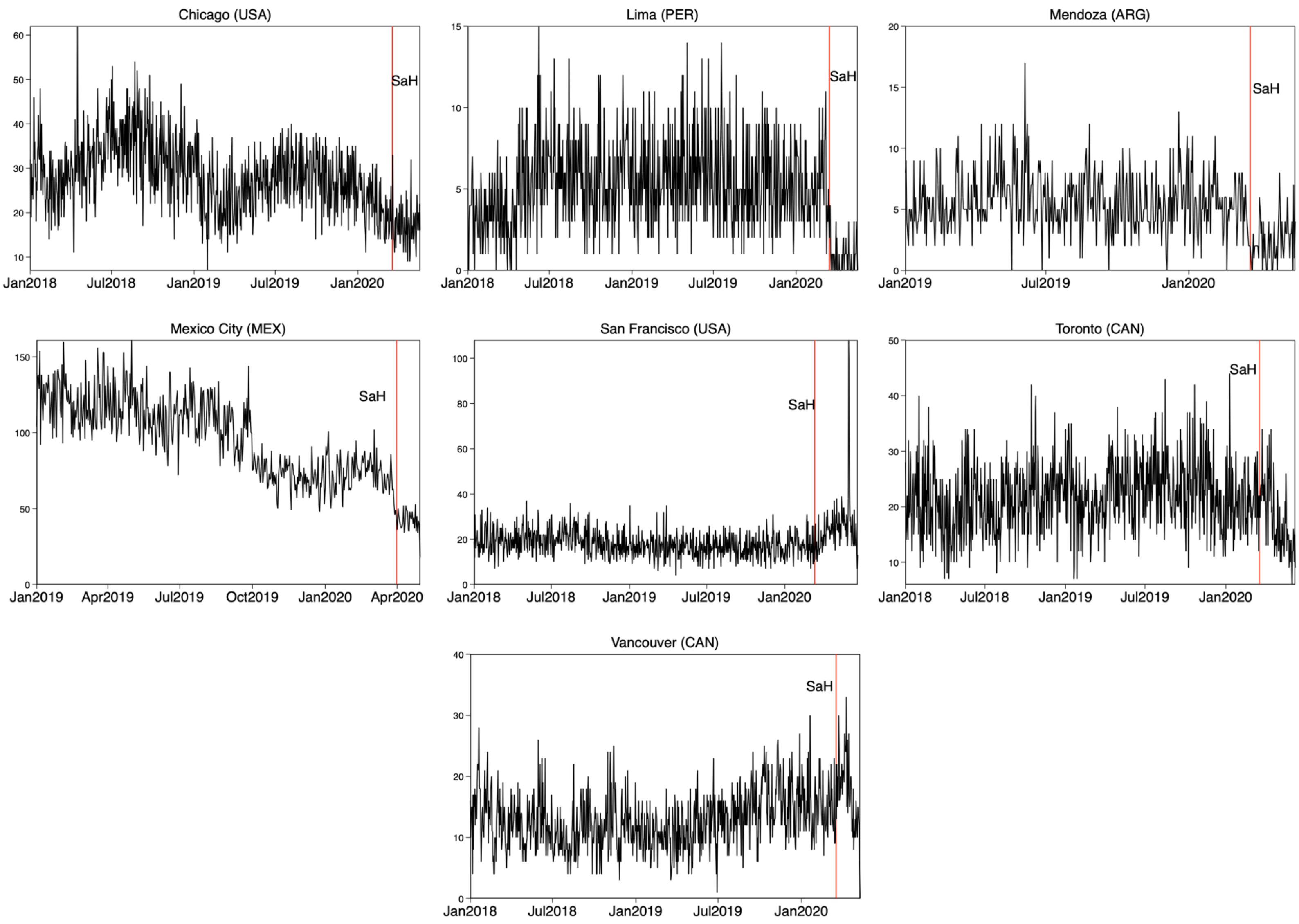

Supplementary Figure 11. Time series plots of daily number of burglaries by cities in the Americas. SaH = Stay at home restrictions implemented. L = Stay at home restrictions lifted.

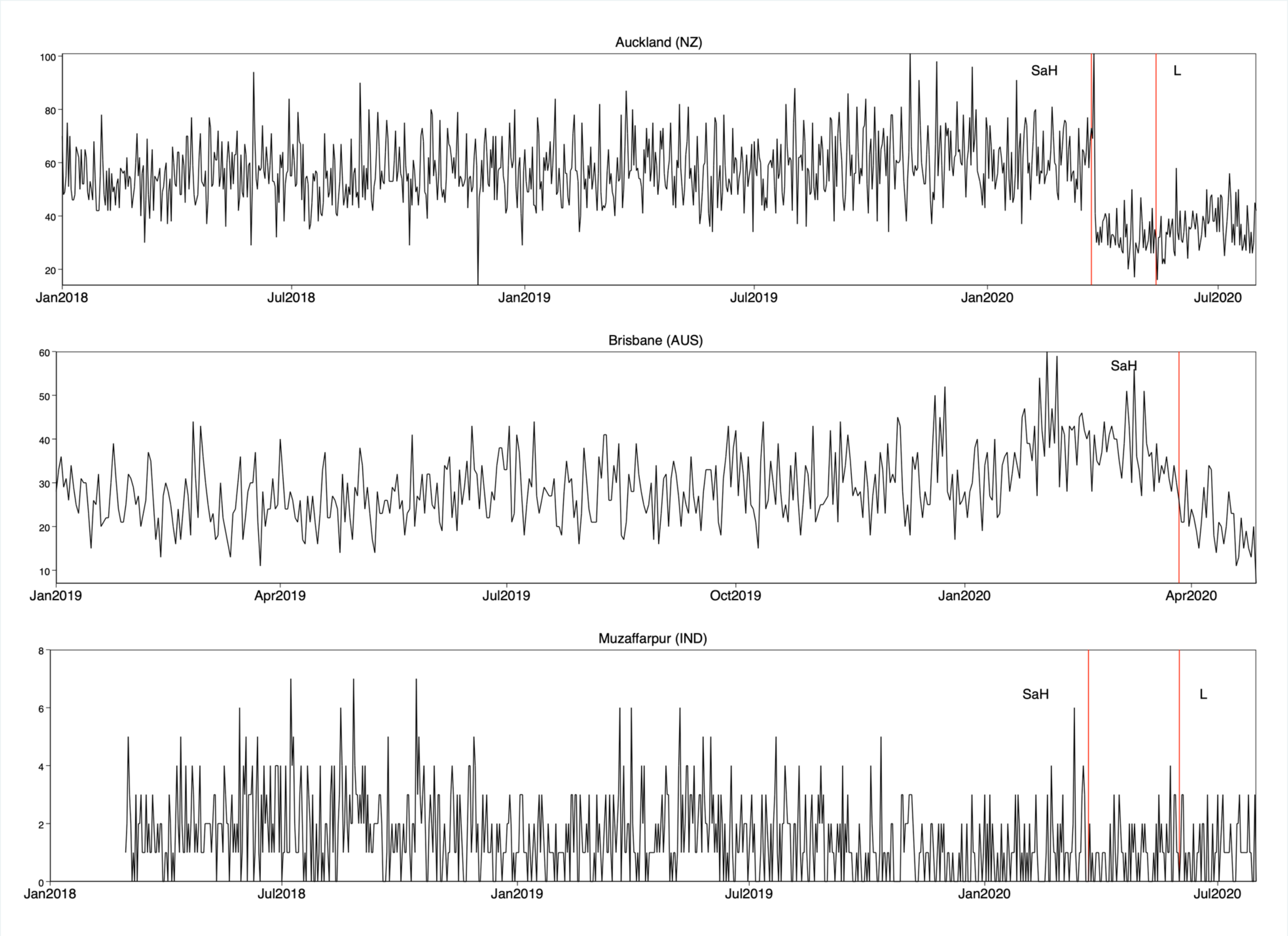

Supplementary Figure 12. Time series plots of daily number of burglaries by cities in Asia and Oceania. SaH = Stay at home restrictions implemented. L = Stay at home restrictions lifted.

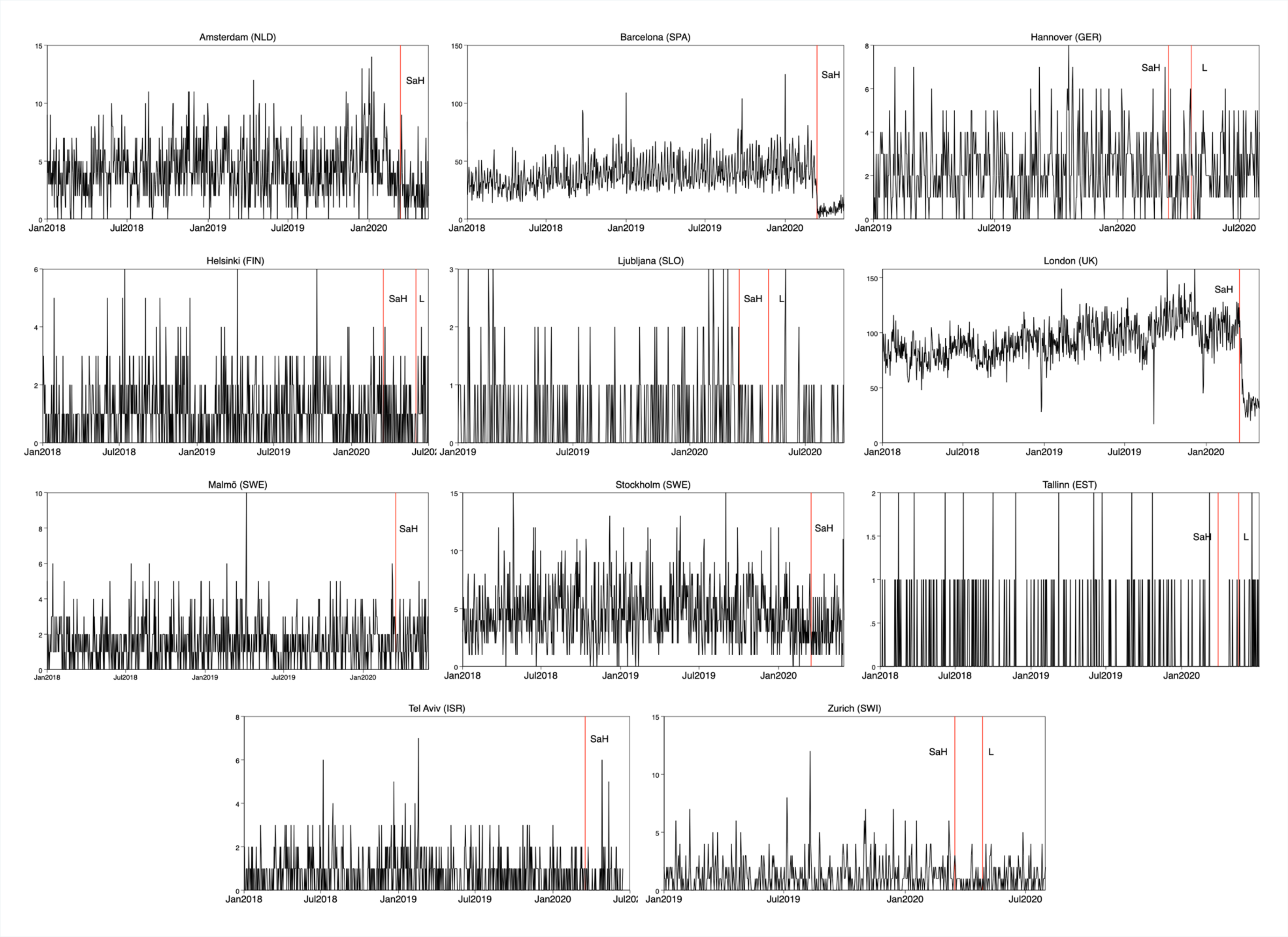

Supplementary Figure 13. Time series plots of daily number of robberies by cities in Europe. SaH = Stay at home restrictions implemented. L = Stay at home restrictions lifted.

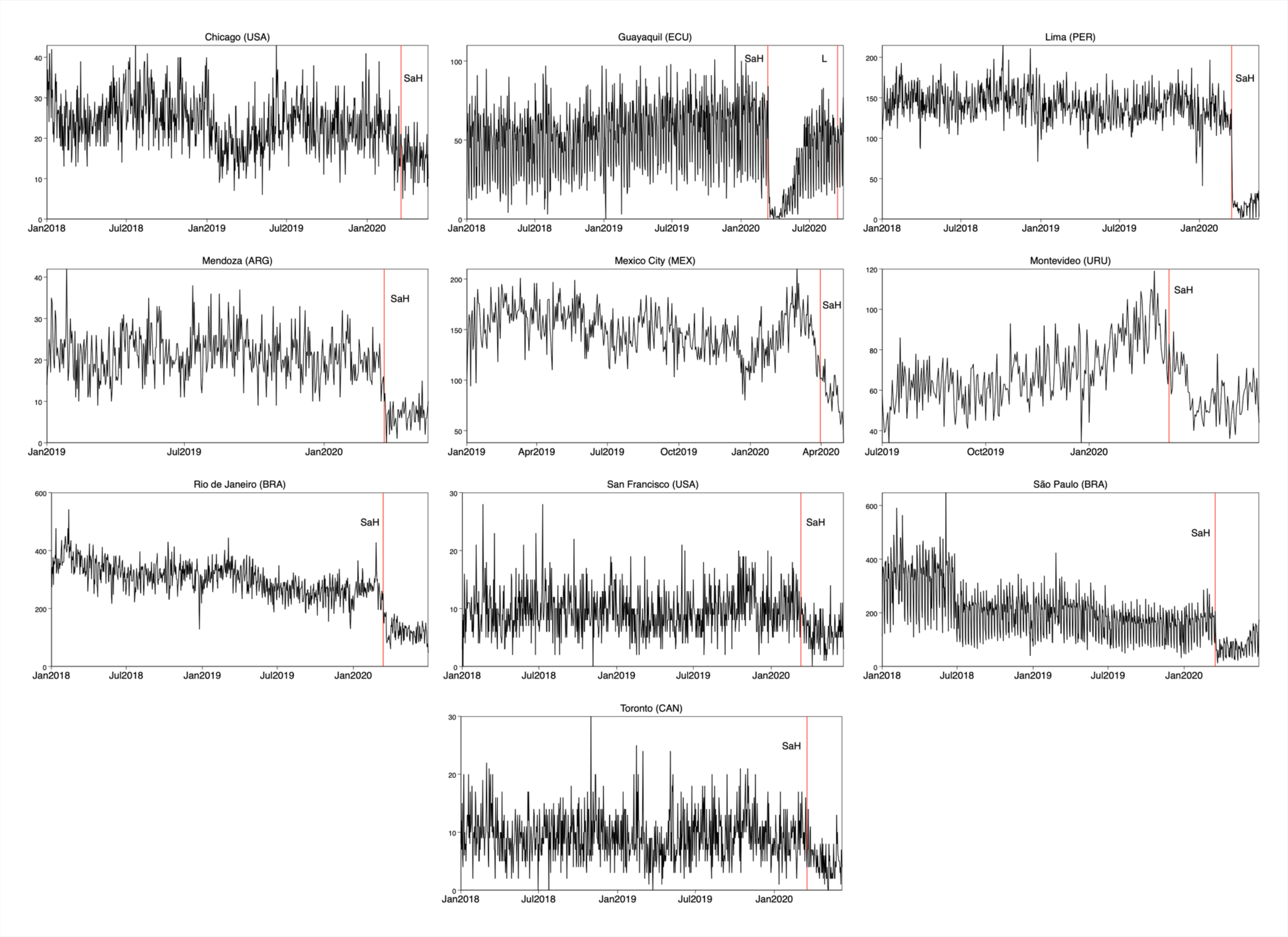

Supplementary Figure 14. Time series plots of daily number of robberies by cities in the Americas. SaH = Stay at home restrictions implemented. L = Stay at home restrictions lifted.

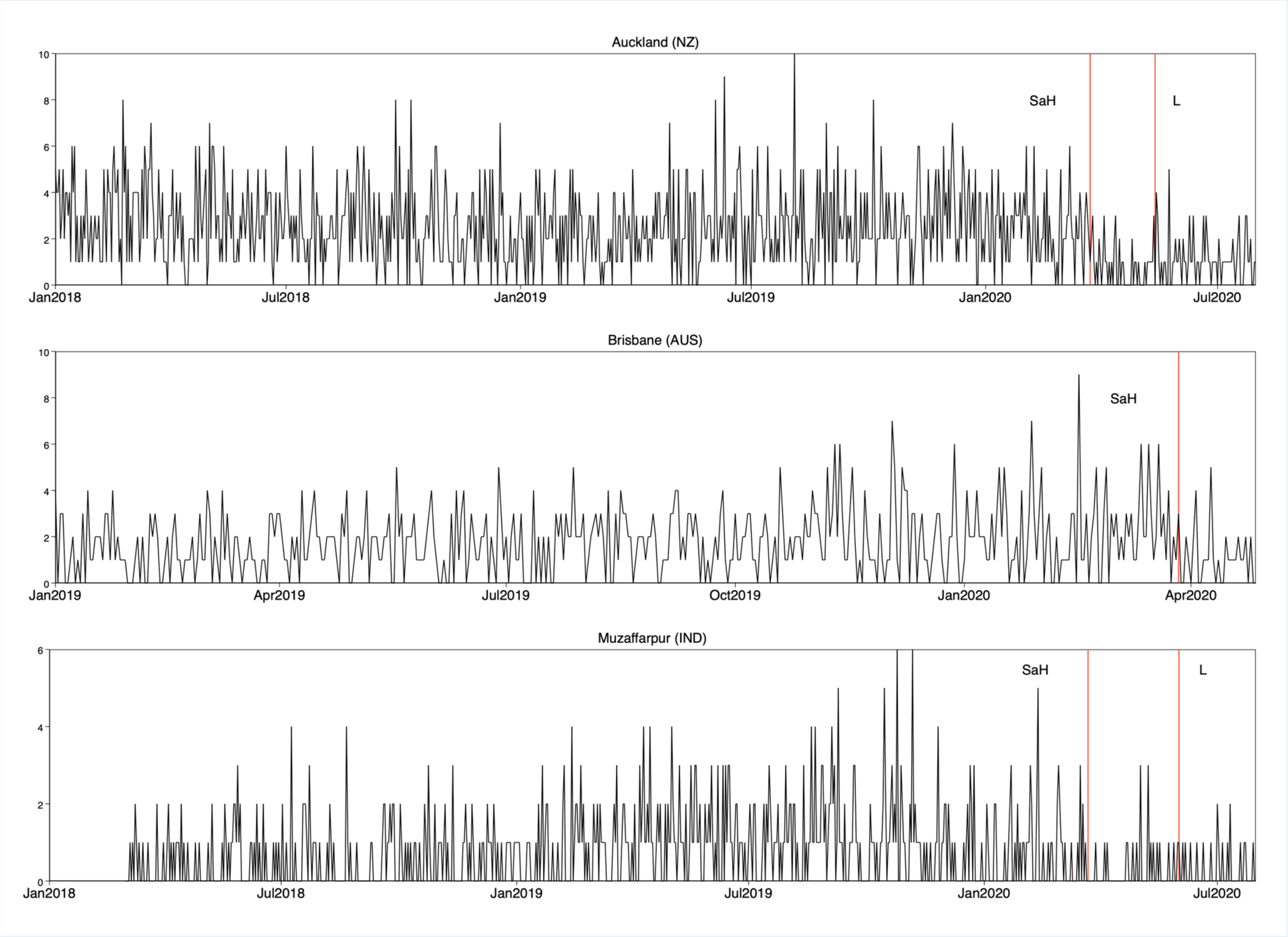

Supplementary Figure 15. Time series plots of daily number of robberies by cities in Asia and Oceania. SaH = Stay at home restrictions implemented. L = Stay at home restrictions lifted.

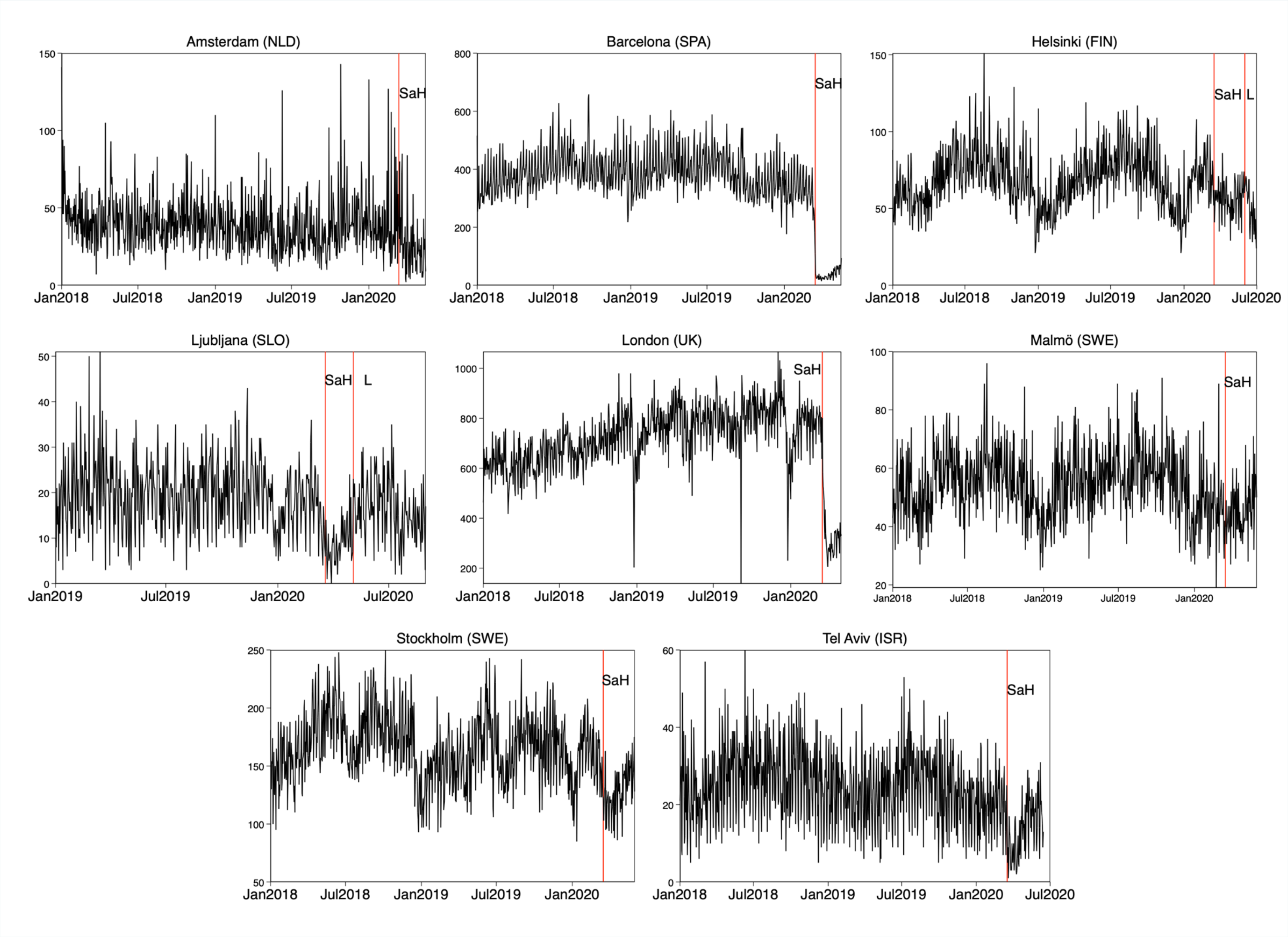

Supplementary Figure 17. Time series plots of daily number of thefts by cities in Europe. SaH = Stay at home restrictions implemented. L = Stay at home restrictions lifted.

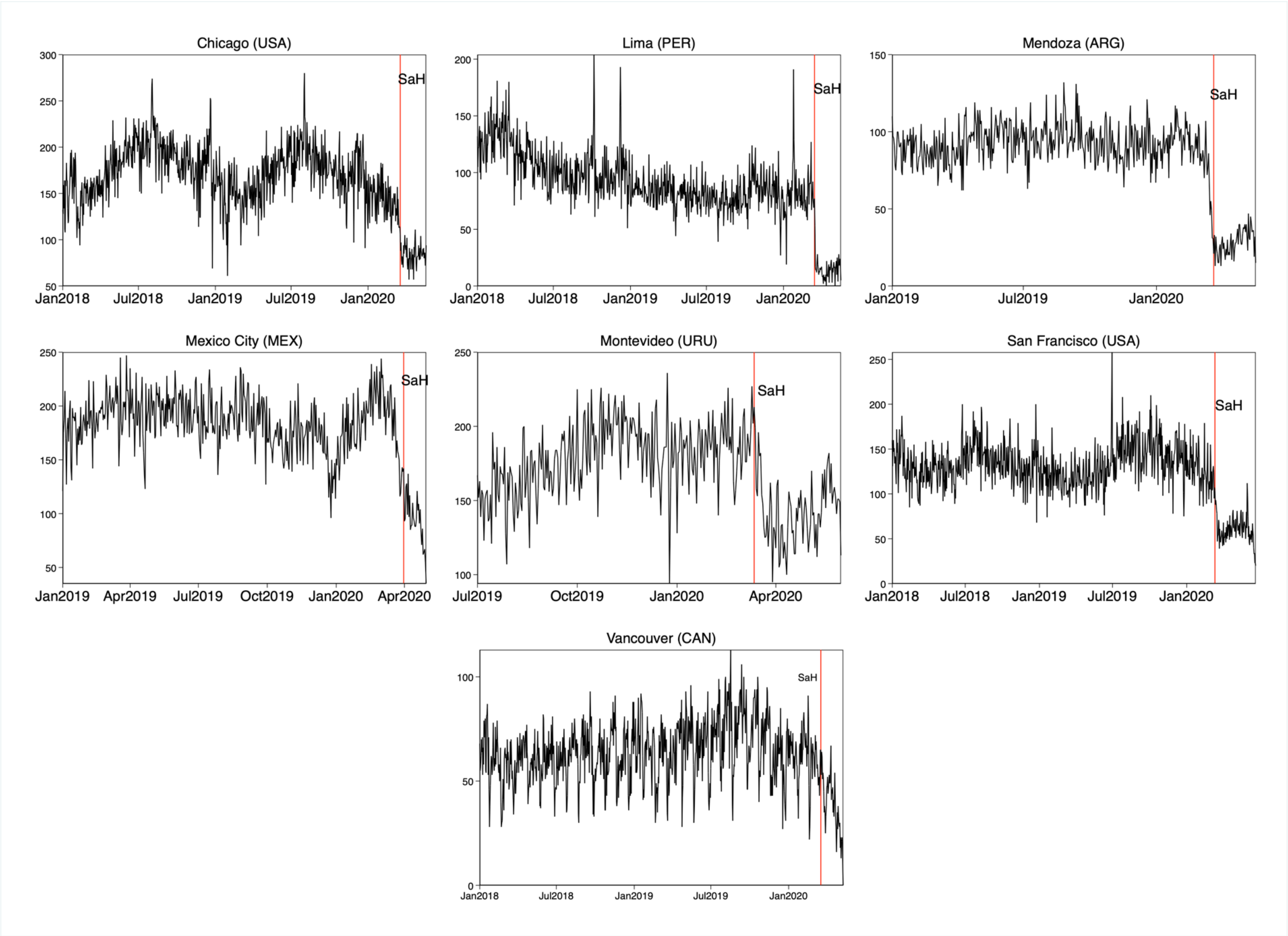

Supplementary Figure 18. Time series plots of daily number of thefts by cities in the Americas. SaH = Stay at home restrictions implemented. L = Stay at home restrictions lifted.

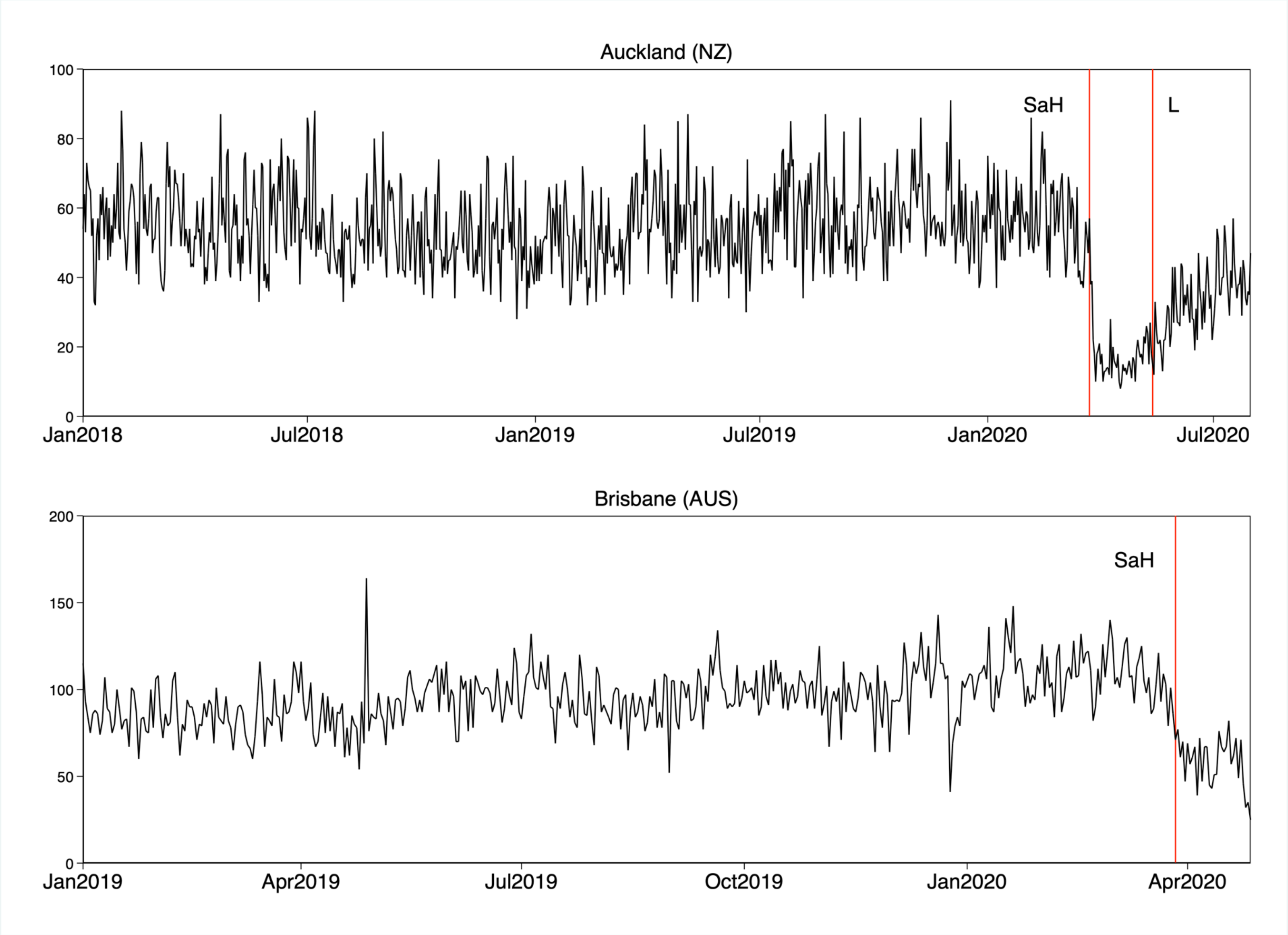

Supplementary Figure 19. Time series plots of daily number of thefts by cities in Oceania. SaH = Stay at home restrictions implemented. L = Stay at home restrictions lifted.

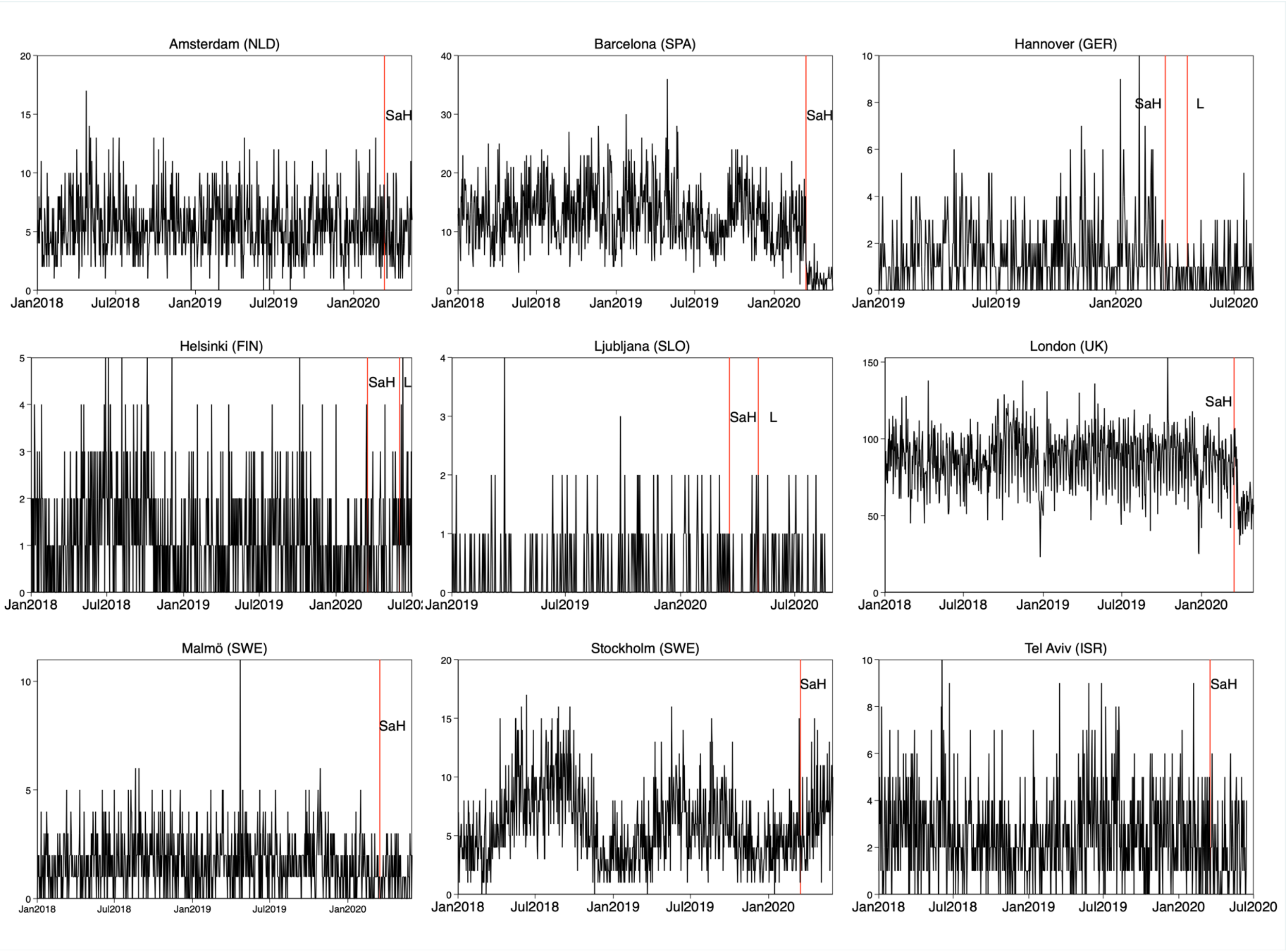

Supplementary Figure 20. Time series plots of daily number of vehicle thefts by cities in Europe. SaH = Stay at home restrictions implemented. L = Stay at home restrictions lifted.

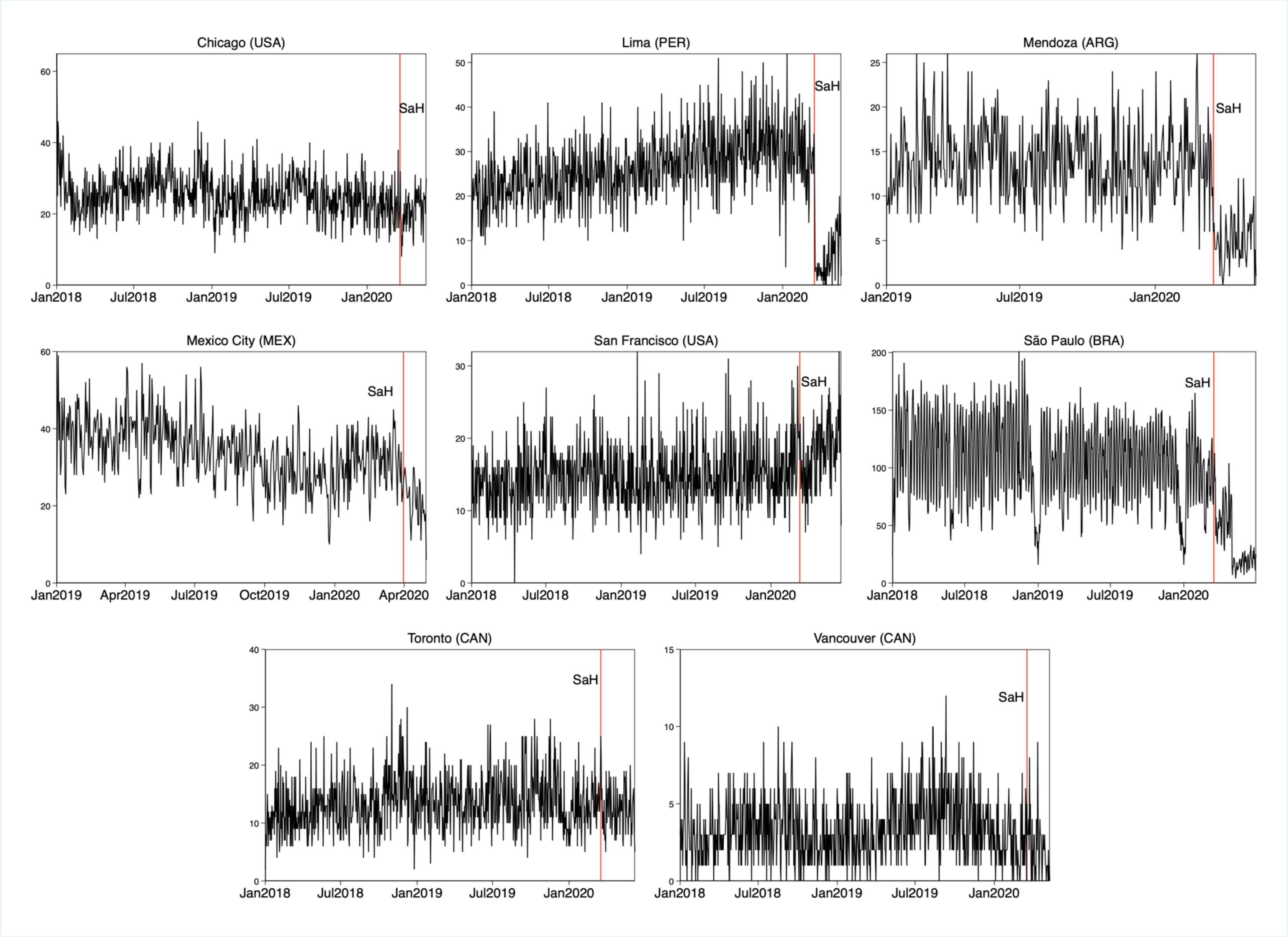

Supplementary Figure 21. Time series plots of daily number of vehicle thefts by cities in the Americas. SaH = Stay at home restrictions implemented. L = Stay at home restrictions lifted.

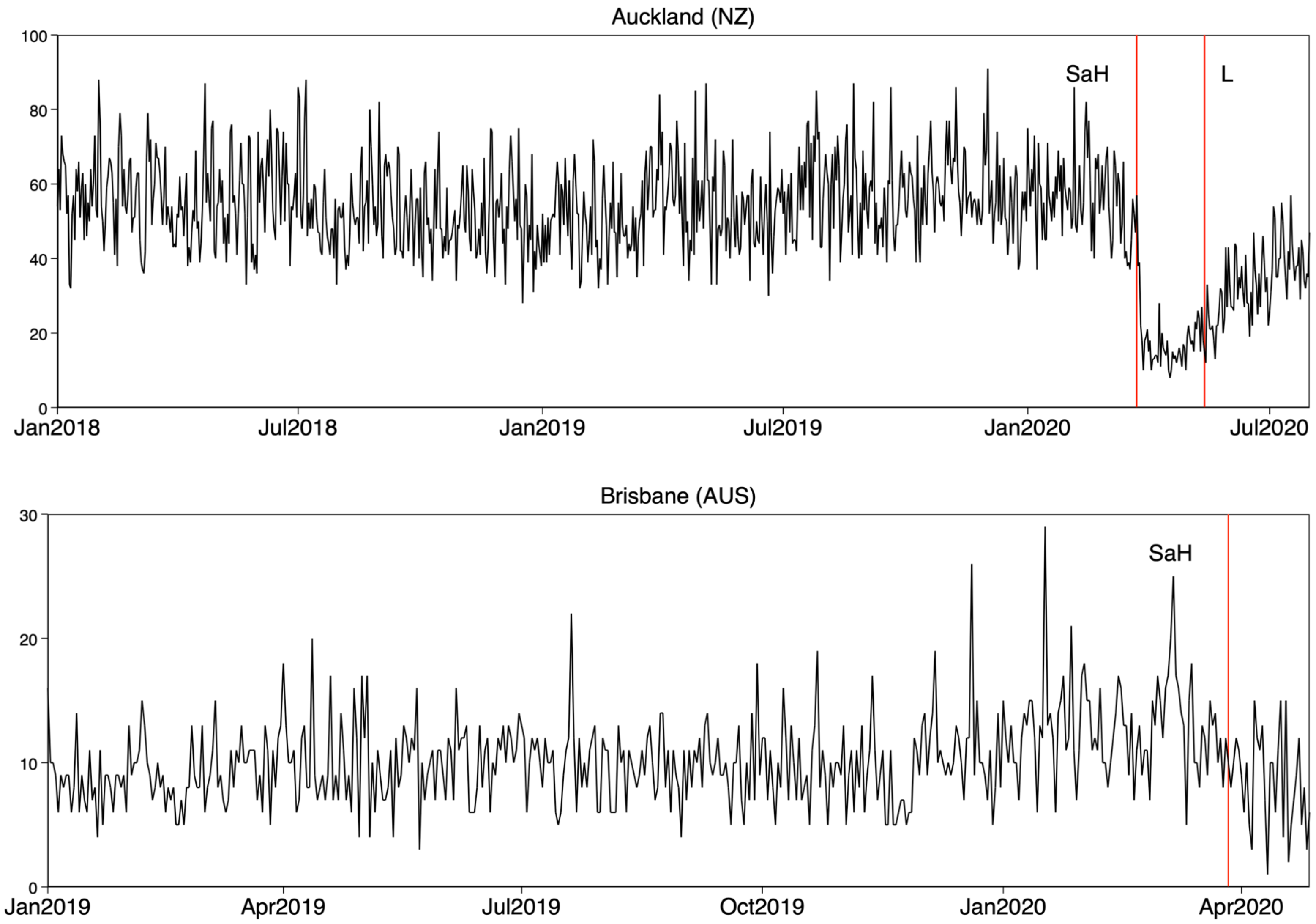

Supplementary Figure 22. Time series plots of daily number of vehicle thefts by cities in Oceania. SaH = Stay at home restrictions implemented. L = Stay at home restrictions lifted.

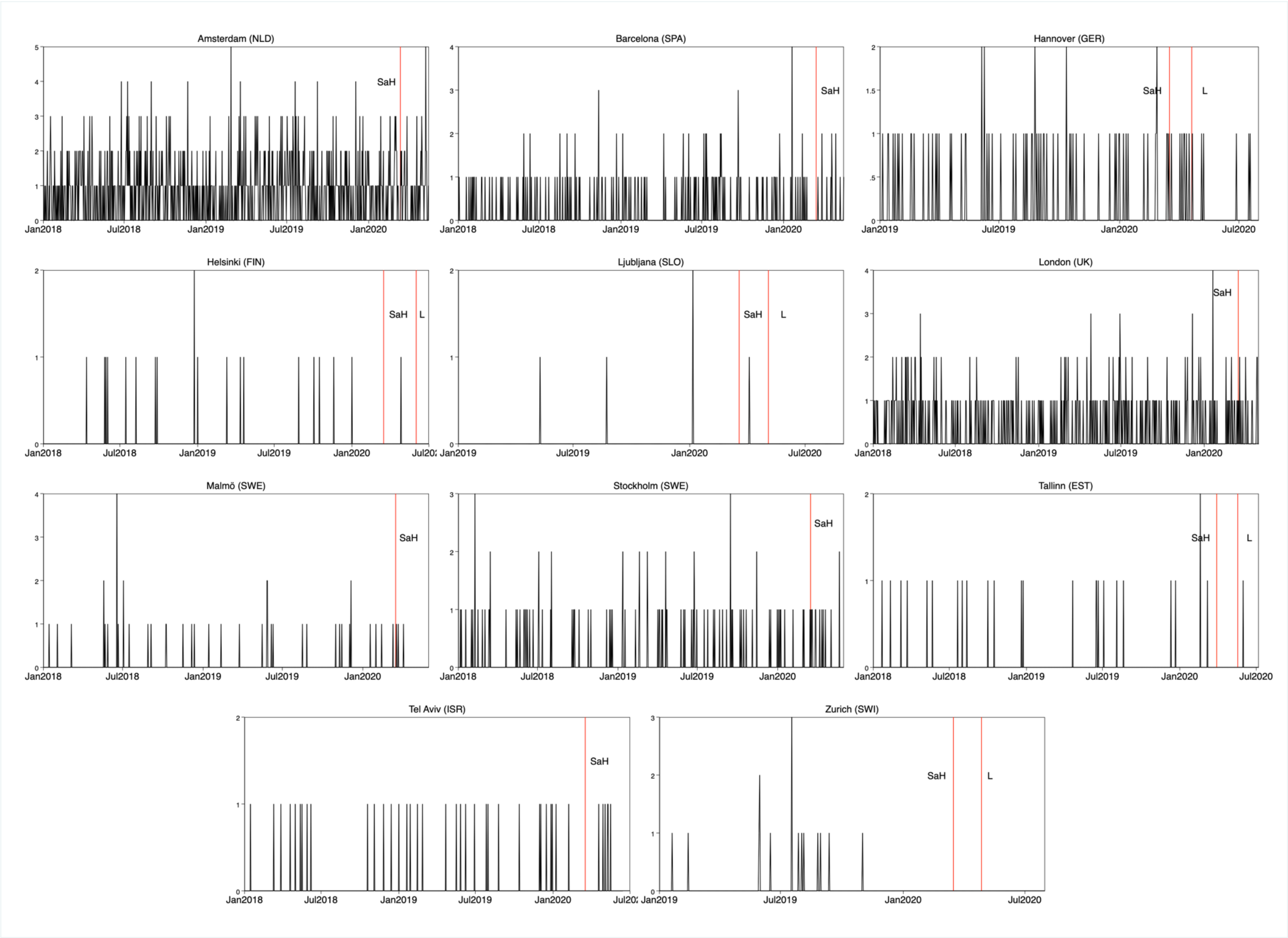

Supplementary Figure 23. Time series plots of daily number of homicides by cities in Europe. SaH = Stay at home restrictions implemented. L = Stay at home restrictions lifted.

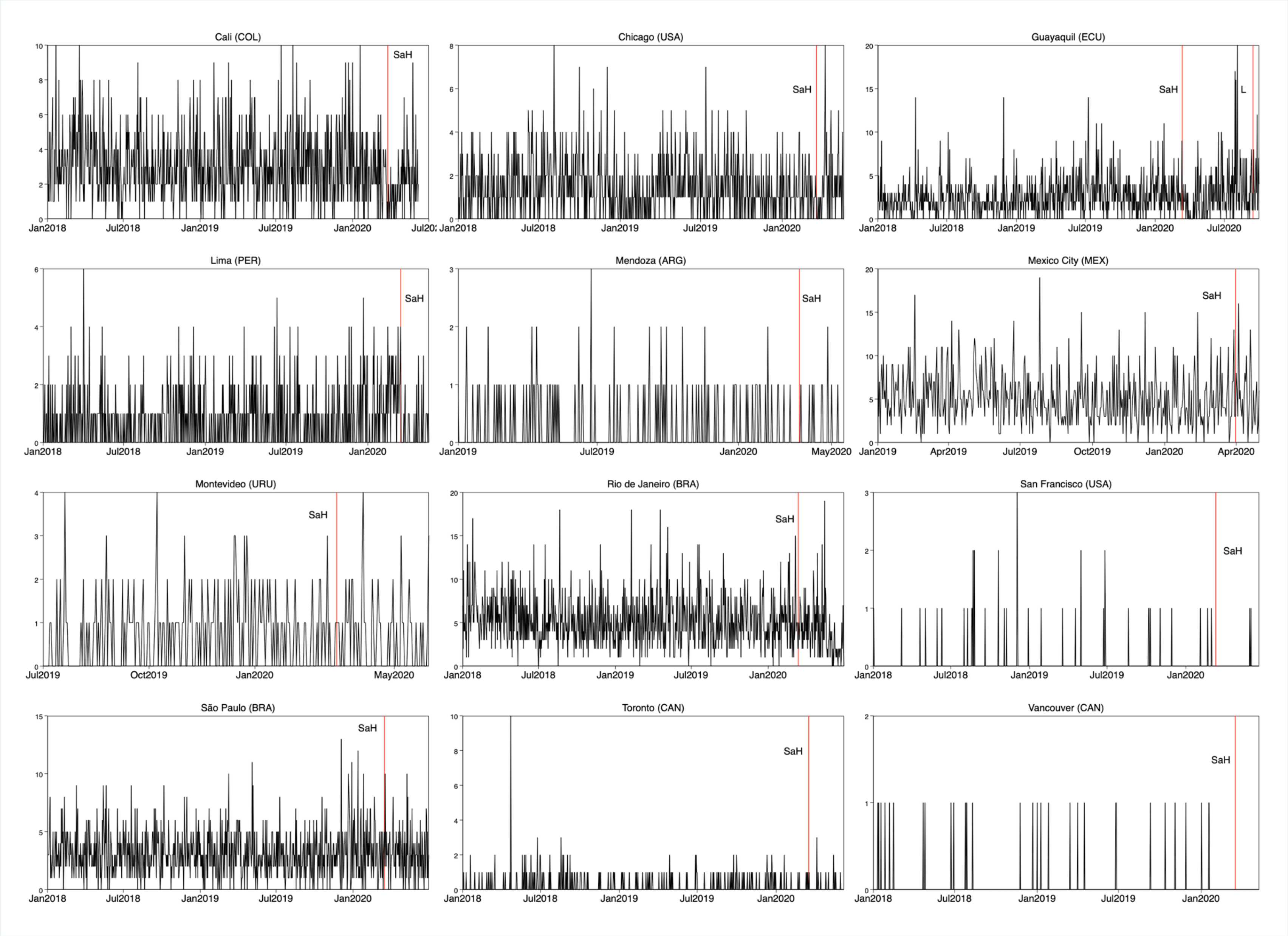

Supplementary Figure 24. Time series plots of daily number of homicides by cities in the Americas. SaH = Stay at home restrictions implemented. L = Stay at home restrictions lifted.

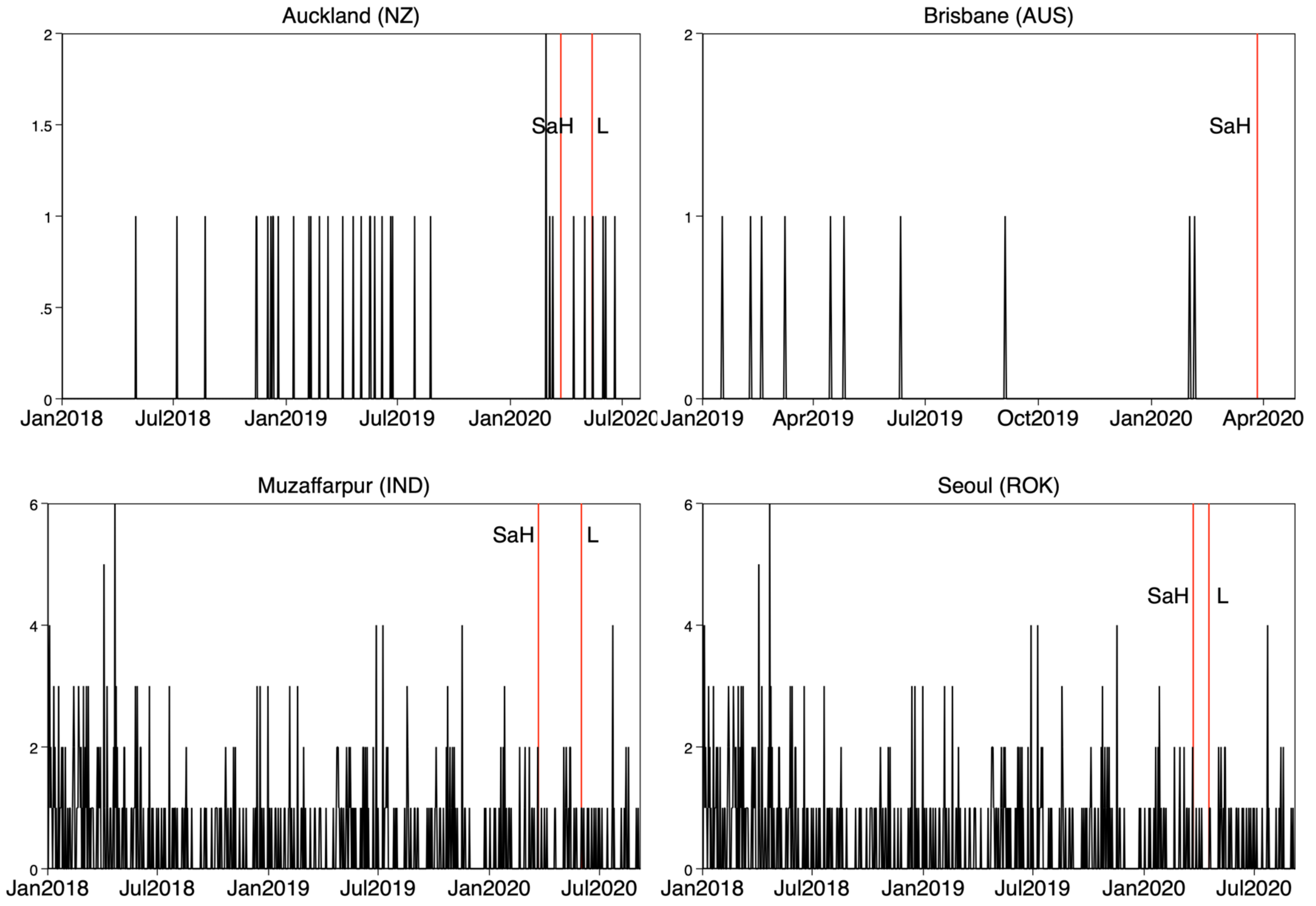

Supplementary Figure 25. Time series plots of daily number of homicides by cities in Asia and Oceania. SaH = Stay at home restrictions implemented. L = Stay at home restrictions lifted.

2.4 Additional information on ITS analyses

Here we provide a detailed description of the interrupted time series analyses. While the data are all count, the number of daily crimes differ across crime types and cities (i.e. from 0 to >500 per day). We aimed for a flexible modelling approach with which we could estimate comparable estimates of effect across models, and that would be appropriate for modelling both low and high counts of daily crime. We therefore opted for a Poisson Generalized Linear Model [GLM] with a logit-link function, as this approach is the most common for handling time series count data, as well as the number of crime counts (*50-51*). The baseline Poisson regression model with treatment effect can be expressed as (*51*):

$$\log\big(E(y|\lambda)\big) = \alpha + x_T\beta_T + x_k\beta_k$$

Where y reflects the expected count of daily crime per category, dependent on the expected rate of the crime outcome (λ) based on a Poisson distribution. The expected outcome is a function of the intercept (α), the treatment variable (x_T) and a set of covariates (x_k). The variable x_T reflects the 'treatment' variable expressed as a step function, whereby 0 represents the period prior to (or following) the implementation of restrictions and 1 represents the period in which restrictions were in place. The models also include a vector of covariates (x_k), including daily temperature, time trend, seasonal dummy variables, and dummy variables for any holidays or outliers. All models include an offset for population and are adjusted for autocorrelation based on the examination of the residuals. In addition, given that in some cases the data tended to be overdispersed, we included in all models an adjustment to estimate the appropriate standard errors as recommended in epidemiological studies (*50*).

Below we provide examples of model specifications for a selection of cities and crime categories. The raw results for all cities and crime types are available in RTF format from the first author upon request.

Amsterdam

- Assault: daily temperature, linear time trend, dummy variables for year, month, week, and day of the week, and two dummy variables for outliers at August 4th and 30th, 2018.
- Theft: daily temperature, linear time trend, dummy variables for year, month, and day of the week, and three dummy variables for outliers at New Year's Day, October 26th and June 9th, 2019, and February 16th, 2020.
- Burglary: daily temperature, linear time trend, dummy variables for year, month, and day of the week, a dummy variable for New Year's Day, and a lagged dependent variable.
- Robbery: daily temperature, linear time trend, dummy variables for year, month, and day of the week, and a dummy variable for New Year's Day.
- Vehicle theft: daily temperature, linear time trend, dummy variables for year, month, and day of the week.
- Homicide: daily temperature, linear time trend, dummy variable for year.

Brisbane

- Assault: daily temperature, linear time trend, dummy variables for year, month, and day of the week, a dummy variable for an outlier at April 28th, 2019, and lagged residuals at lag 4.

- Theft: daily temperature, linear time trend, dummy variables for year, month, and day of the week, dummy variable for Christmas and an outlier at April 28th, 2019, and lagged residual at lag 1.
- Burglary: daily temperature, linear time trend, dummy variables for year, month, and day of the week, and lagged residuals at lag 1.
- Robbery: daily temperature, linear time trend, dummy variables for year and day of the week.
- Vehicle theft: daily temperature, linear time trend, dummy variables for year, month, week, and day of the week, dummy variable for outlier at December 20th, 2019, and lagged residuals at lag 1.
- Homicide: too few cases.

Chicago

- Assault: daily temperature, linear time trend, dummy variables for year, month, and day of the week, a dummy variable for New Year's Day, and lagged residuals at lags 1 and 2.
- Theft: daily temperature, linear time trend, dummy variables for year, month, and day of the week, dummy variables for Christmas and an outlier at August 2nd-3rd, 2018 and 2019, lagged dependent variable at lag 1, and lagged residuals at lags 1 and 2.
- Burglary: daily temperature, linear time trend, dummy variables for year, month, and day of the week, and lagged residuals at lags 1 and 5.
- Robbery: daily temperature, linear time trend, dummy variables for year and day of the week, and lagged residuals at lags 1 and 2.
- Vehicle theft: daily temperature, linear time trend, dummy variables for year, month, week, and day of the week, dummy variable for New Year's Day, and lagged residuals at lags 1 through 3.
- Homicide: daily temperature, linear time trend, dummy variables for year, month, week, and day of the week, and lagged residuals at lag 1.

2.4.1 Lima with and without temperature data

Temperature data for Lima was not available for the period between January and May 2018. The results presented in the main paper for Lima were estimated without temperature data. Since average temperature could act as a potential confounder, we re-estimated all models with the available climate data starting from May 2018. The inclusion of daily average temperature as a covariate does not affect the main results (see Supplementary Table 16). The full results for Lima with and without temperature as a covariate are available in RTF format from the first author upon request.

		SaH restrictions	
Crime category	Estimate	Excl. temp.	Incl. temp.
Assault	b	-1.37	-1.32
	SE	0.06	0.07
	IRR	0.25	0.27
	N	866	725
Burglary	b	-1.81	-1.80
	SE	0.16	0.16
	IRR	0.16	0.17
	N	854	712
Robbery	b	-1.87	-1.84
	SE	0.05	0.05
	IRR	0.15	0.16
	N	846	722
Theft	b	-1.14	-1.11
	SE	0.07	0.08
	IRR	0.32	0.33
	N	854	706
Vehicle theft	b	-1.54	-1.54
	SE	0.07	0.07
	IRR	0.21	0.21
	N	861	719
Homicide	b	-1.43	-1.45
	SE	0.26	0.28
	IRR	0.24	0.23
	N	868	726

Supplementary Table 16. Estimates of the level change in number of crimes per day following the implementation of stay at home restrictions in Lima, Peru, with and without daily temperature as a covariate. All p-values are below 0.001. SaH=stay at home restrictions; b=unstandardized coefficient; SE=standard error; IRR=incidence rate ratio; N=number of days (sample size).

2.5 Additional meta-analytic and meta-regression analyses

A series of additional analyses were conducted in order to evaluate the sensitivity of the meta-analytic and meta-regression analyses. First, we calculated the summary results by city instead of crime type in order to examine overall city trends for the crimes included (Supplementary Table 17).

City	Crime	ES	95%CI Lower	95%CI Upper	Heterogeneity statistic	p-value	tau^2	I^2
Amsterdam	Assault	0.83	0.72	0.95	12.19	0.03	0.01	59.00%
	Theft	0.70	0.59	0.83				
	Burglary	0.89	0.76	1.04				
	Robbery	0.58	0.46	0.74				
	Vehicle Theft	0.76	0.64	0.91				
	Homicide	0.99	0.67	1.47				
	Summary	0.77	0.68	0.87				
Auckland	Assault	0.94	0.87	1.02	201.24	<0.001	0.15	97.50%

	Theft	0.61	0.54	0.68				
	Burglary	0.86	0.78	0.95				
	Robbery	0.47	0.34	0.66				
	Vehicle Theft	0.41	0.37	0.45				
	Homicide	0.76	0.17	3.35				
	Summary	0.63	0.45	0.89				
Barcelona	Assault	0.16	0.14	0.18	274.66	<0.001	0.29	98.20%
	Theft	0.11	0.10	0.13				
	Burglary	0.39	0.34	0.44				
	Robbery	0.20	0.18	0.23				
	Vehicle Theft	0.21	0.16	0.26				
	Homicide	1.57	0.59	4.18				
	Summary	0.24	0.15	0.38				
Brisbane	Assault	0.63	0.52	0.77	4.83	0.31	0.002	17.10%
	Theft	0.57	0.52	0.61				
	Burglary	0.43	0.27	0.68				
	Robbery	0.42	0.27	0.66				
	Vehicle Theft	0.61	0.51	0.73				
	Summary	0.57	0.53	0.63				
Cali	Homicide	0.71	0.56	0.90	NA		NA	NA
	Summary	0.71	0.56	0.90				
Chicago	Assault	0.66	0.62	0.70	69.01	<0.001	0.03	92.80%
	Theft	0.63	0.58	0.68				
	Burglary	0.81	0.74	0.89				
	Robbery	0.75	0.68	0.83				
	Vehicle Theft	0.93	0.86	1.01				
	Homicide	0.90	0.64	1.25				
	Summary	0.76	0.66	0.87				
Guayaquil	Assault	0.66	0.42	1.03	63.64	<0.001	0.37	96.90%
	Robbery	0.33	0.28	0.38				
	Homicide	0.84	0.71	1.01				
	Summary	0.56	0.28	1.15				
Hannover	Assault	0.80	0.69	0.91	7.62	0.11	0.02	47.50%
	Burglary	0.80	0.64	0.99				
	Robbery	0.86	0.64	1.16				
	Vehicle Theft	0.51	0.29	0.89				
	Homicide	2.03	0.88	4.73				
	Summary	0.81	0.67	0.97				
Helsinki	Assault	0.74	0.63	0.87	8.56	0.07	0.01	53.30%
	Theft	0.86	0.81	0.91				
	Burglary	0.63	0.48	0.83				
	Robbery	0.79	0.54	1.14				
	Vehicle Theft	1.03	0.71	1.50				
	Summary	0.80	0.70	0.91				
Lima	Assault	0.25	0.23	0.29	87.14	<0.001	0.10	94.30%
	Theft	0.32	0.28	0.37				
	Burglary	0.16	0.12	0.22				
	Robbery	0.15	0.14	0.17				
	Vehicle Theft	0.21	0.19	0.25				
	Homicide	0.24	0.14	0.40				

	Summary	0.22	0.17	0.28				
Ljubljana	Theft	0.55	0.47	0.64	9.87	0.02	0.05	69.60%
	Burglary	0.84	0.67	1.04				
	Robbery	0.61	0.32	1.19				
	Vehicle Theft	0.77	0.40	1.50				
	Summary	0.68	0.50	0.90				
London	Assault	0.91	0.87	0.94	360.84	<0.001	0.12	98.60%
	Theft	0.54	0.47	0.62				
	Burglary	0.71	0.66	0.77				
	Robbery	0.40	0.37	0.43				
	Vehicle Theft	0.74	0.69	0.80				
	Homicide	0.84	0.41	1.69				
	Summary	0.65	0.49	0.87				
Malmö	Assault	0.91	0.77	1.09	15.18	0.01	0.02	67.10%
	Theft	0.91	0.84	0.98				
	Burglary	1.31	1.10	1.56				
	Robbery	0.96	0.70	1.32				
	Vehicle Theft	0.84	0.60	1.17				
	Homicide	0.64	0.11	3.76				
	Summary	0.98	0.84	1.14				
Mexico City	Assault	0.55	0.45	0.67	19.12	0.002	0.01	73.90%
	Theft	0.60	0.55	0.64				
	Burglary	0.58	0.52	0.64				
	Robbery	0.59	0.54	0.64				
	Vehicle Theft	0.64	0.57	0.72				
	Homicide	1.14	0.83	1.56				
	Summary	0.62	0.56	0.68				
Mendoza	Assault	0.48	0.44	0.53	59.42	<0.001	0.05	91.60%
	Theft	0.30	0.28	0.33				
	Burglary	0.38	0.30	0.48				
	Robbery	0.33	0.28	0.37				
	Vehicle Theft	0.34	0.29	0.40				
	Homicide	0.87	0.41	1.87				
	Summary	0.38	0.31	0.46				
Montevideo	Assault	0.71	0.57	0.88	1.84	0.40	0.00	0.00%
	Robbery	0.76	0.69	0.83				
	Homicide	1.08	0.61	1.91				
	Summary	0.76	0.69	0.82				
Muzaffarpur	Burglary	0.78	0.56	1.09	5.75	0.08	0.06	65.20%
	Robbery	0.44	0.25	0.79				
	Homicide	1.03	0.71	1.50				
	Summary	0.75	0.50	1.13				
Rio de Janeiro	Assault	0.44	0.41	0.48	119.78	<0.001	0.06	97.50%
	Robbery	0.50	0.48	0.52				
	Vehicle Theft	0.75	0.69	0.81				
	Homicide	0.76	0.64	0.91				
	Summary	0.59	0.47	0.75				
Seoul	Assault	1.07	1.01	1.14	2.1	0.15	0.26	52.30%
	Homicide	0.40	0.10	1.53				
	Summary	0.83	0.35	1.95				

San Francisco	Assault	0.64	0.58	0.70	314.06	<0.001	0.18	98.40%
	Theft	0.54	0.51	0.58				
	Burglary	1.38	1.25	1.52				
	Robbery	0.63	0.54	0.74				
	Vehicle Theft	1.02	0.93	1.13				
	Homicide	0.30	0.04	2.25				
	Summary	0.77	0.53	1.11				
São Paulo	Assault	0.77	0.73	0.82	227.70	<0.001	0.18	98.70%
	Robbery	0.54	0.50	0.59				
	Vehicle Theft	0.30	0.26	0.33				
	Homicide	0.90	0.72	1.11				
	Summary	0.56	0.38	0.87				
Stockholm	Assault	0.91	0.81	1.03	36.57	<0.001	0.02	86.30%
	Theft	0.74	0.71	0.77				
	Burglary	0.96	0.87	1.06				
	Robbery	0.77	0.62	0.96				
	Vehicle Theft	0.94	0.77	1.15				
	Homicide	1.62	0.61	4.31				
	Summary	0.87	0.75	1.00				
Tallinn	Assault	0.84	0.71	0.98	0.76	0.39	0.00	0.00%
	Robbery	1.09	0.61	1.97				
	Summary	0.85	0.73	0.99				
Tel Aviv	Assault	0.77	0.67	0.88	9.22	0.10	0.01	45.80%
	Theft	0.62	0.55	0.70				
	Burglary	0.74	0.61	0.90				
	Robbery	0.89	0.55	1.42				
	Vehicle Theft	0.71	0.55	0.92				
	Homicide	1.42	0.50	4.01				
	Summary	0.72	0.64	0.81				
Toronto	Assault	0.70	0.65	0.76	22.82	<0.001	0.02	82.50%
	Burglary	0.85	0.77	0.94				
	Robbery	0.65	0.55	0.78				
	Vehicle Theft	0.92	0.82	1.04				
	Homicide	0.73	0.37	1.41				
	Summary	0.78	0.67	0.90				
Vancouver	Theft	0.62	0.57	0.67	86.52	<0.001	0.18	97.70%
	Burglary	1.21	1.08	1.36				
	Vehicle Theft	0.81	0.62	1.07				
	Summary	0.85	0.52	1.39				
Zurich	Assault	0.71	0.54	0.94	2.61	0.27	0.01	23.40%
	Burglary	0.87	0.72	1.06				
	Robbery	0.64	0.43	0.94				
	Summary	0.77	0.65	0.92				
Overall	Summary	0.63	0.58	0.69	7006.91	<0.001	0.19	98.20%

Supplementary Table 17. Meta-analytic results reporting summary effect sizes by city, including overall effect size for all crimes and cities in the sample. ES=effect size, CI=confidence interval, p-values refer to the heterogeneity statistic. Overall summary effects are estimated using random effects meta-analytic techniques.

Second, because the differences across crime types might be sensitive to the number of cities included, we re-estimated the summary effects using only cities that reported all crime categories.

The results are reported in Supplementary Table 18. The overall effect sizes do not substantially change when evaluating only cities with all available crime categories, however the confidence intervals for the summary homicide effect now overlap 1 (Supplementary Table 18, 95%CI=0.61-1.19). This is likely because two of the three cities that saw significant declines in homicide (Cali and Rio de Janeiro) are excluded from the analyses. This suggests that the summary results for homicide may be driven in part by these two cases.

	Overall ES	95%CI Lower	95%CI Upper	Heterogenity statistic	p-value	tau^2	I^2	N
Assault	0.60	0.46	0.77	1063.52	<0.001	0.21	99.00%	12
Burglary	0.68	0.54	0.86	497.40	<0.001	0.16	97.80%	12
Robbery	0.49	0.35	0.68	869.93	<0.001	0.34	98.70%	12
Theft	0.50	0.37	0.66	1683.50	<0.001	0.26	99.30%	12
Vehicle theft	0.57	0.43	0.76	687.61	<0.001	0.25	98.40%	12
Homicide	0.85	0.61	1.19	32.92	0.001	0.19	66.60%	12

Supplementary Table 18. Summary effect sizes from meta-analyses using only cities with all available crime categories (n=12). ES=effect size, CI=confidence interval, p-values refer to the heterogeneity statistic. Overall summary effects are estimated using random effects meta-analytic techniques.

Third, during the course of the meta-regression analyses, we inspected the scatterplots of predicted random effects estimates against average stringency of stay at home restrictions (Supplementary Figure 26). The scatterplots revealed Barcelona as a potential outlier. This is particularly the case for assault and theft, where Barcelona experienced significant declines in all crimes except homicide, yet has an average rating of 2 for the stringency of stay at home restrictions. In order to examine the potential influence this outlier has on the meta-regression results, we estimated all models with and without Barcelona. The results suggest that, when Barcelona is excluded from the meta-regression analyses, more stringent stay at home restrictions are significantly associated with more negative effect sizes (i.e. larger declines) for assault (Supplementary Table 19). The model fit for assault did not substantially improve with the exclusion of the outlier. However for theft the proportion of variance explained by the stringency of stay at home restrictions increased to 57.89%. The results remained largely similar for burglary, robbery, vehicle theft, and homicide.

		Assault	Burglary	Robbery	Theft	Vehicle theft	Homicide
Stringency of stay at home restrictions	b	-0.21	-0.35	-0.38	-0.27	-0.36	-0.27
	SE	(0.09)	(0.12)	(0.10)	(0.06)	(0.12)	(0.16)
	p-value	0.032	0.008	0.002	0.001	0.010	0.112
	95% CI	(-0.40, -0.02)	(-0.59, -0.11)	(-0.59, -0.16)	(-0.41, -0.14)	(-0.62, -0.10)	(-0.60, 0.07)
	exp(b)	0.81	0.71	0.69	0.76	0.70	0.77
	tau^2	0.08	0.14	0.11	0.04	0.14	0.06
	Adj. R^2	17.53%	34.21%	40.48%	57.89%	30.27%	19.29%
	N	22	19	23	15	19	20

Supplementary Table 19. Meta-regression results for the stringency of stay at home restrictions on the size of the effect of stay at home orders on crime excluding Barcelona. 95% CI=95% confidence intervals. Exp(b) reflects the standardized exponentiated coefficient. Adj. (adjusted) R^2 reflects the proportion of variance in the effect sizes explained by the given covariate.

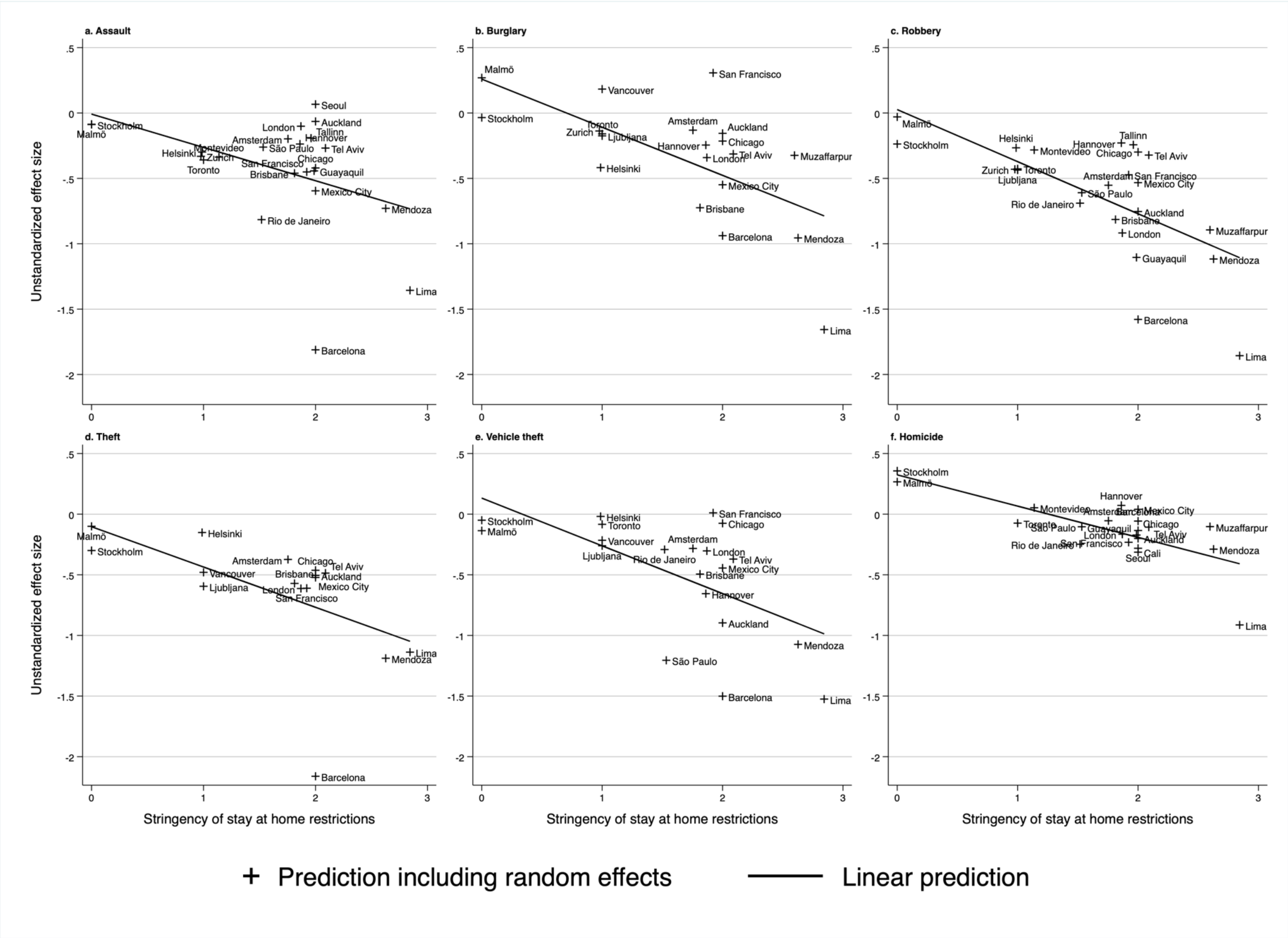

Supplementary Figure 26. Scatterplots of predicted effect size including random effects against the stringency of stay at home restrictions. Estimated predictions are drawn from meta-regression analyses.

In addition, for some cities and countries, domestic or family assault is not distinguished in police data from non-domestic assault incidents. Since there is some reason to believe that domestic and non-domestic assaults may react differently to the implementation of stay at home restrictions, we re-estimated the meta-analytic and meta-regression results excluding these cases (Amsterdam, Helsinki, Toronto, Tallinn, and London).

The results suggest that the summary effect size is not substantially affected by the exclusion of these cities (overall ES=0.60, 95%CI=0.47-0.75, tau^2=0.20, I^2=98.9%). The meta-regression results reported in Supplementary Table 20 are similarly in line with the results reported for assault in Table 2 in the main text, and Supplementary Table 19 excluding Barcelona. The model fit, as measured by the proportion of variance explained, improves slightly when excluding both Barcelona and the cities including domestic-type assaults (from 18.62% to 24.25%). However, the results for the overall stringency index remain non-significant even when excluding Barcelona.

	incl. Barcelona	excl. Barcelona
b	-0.29	-0.25
SE	(0.15)	(0.11)
p-value	0.08	0.04
exp(b)	0.75	0.78
tau^2	0.21	0.10
Adj. R^2	14.83%	24.11%
N	16	15

Supplementary Table 20. Meta-regression results for the stringency of stay at home restrictions on the size of the effect for assault excluding cities where domestic-type assaults are not clearly distinguished within police records. Exp(b) reflects the standardized exponentiated coefficient. Adj. (adjusted) R^2 reflects the proportion of variance in the effect sizes explained by the given covariate.

Next, given that most COVID-19-related stay at home restrictions were implemented alongside a wide array of other containment policies that may have influenced the degree of decline instead, or in addition to, the stringency of stay at home orders. We therefore re-estimated a series of meta-regression analyses that evaluate the relationship between the stringency of school closures, workplace closures, restrictions on public events, restrictions on private gatherings, restrictions on public transportation, restrictions on internal travel, and overall stringency index. In addition, countries or cities that implemented economic policies to mitigate the negative effects of economic strain due to COVID-19-related policies (i.e. unemployment, income loss). By mitigating economic strain, there may be less motivation for potential offenders to cope through crime, resulting in greater, more negative effects on crime levels. We therefore expect that higher scores on the economic index would be associated with more negative effects of stay at home restrictions on crime. The results are presented in Supplementary Table 21.

		Assault	Burglary	Robbery	Theft	Vehicle theft	Homicide
Closing schools	b	-0.23	-0.21	-0.31	-0.35	-0.28	-0.24
	SE	(0.15)	(0.17)	(0.16)	(0.17)	(0.18)	(0.25)
	p-value	0.14	0.23	0.07	0.05	0.14	0.35
	exp(b)	0.80	0.81	0.73	0.70	0.76	0.79
	tau^2	0.18	0.21	0.19	0.20	0.23	0.08
	Adj. R^2	5.87%	3.04%	11.40%	19.16%	7.02%	4.34%
Workplace restrictions	b	-0.21	-0.17	-0.23	-0.30	-0.23	-0.15
	SE	(0.14)	(0.18)	(0.15)	(0.18)	(0.19)	(0.17)
	p-value	0.14	0.35	0.14	0.11	0.23	0.41
	exp(b)	0.81	0.85	0.80	0.74	0.79	0.86
	tau^2	0.18	0.22	0.20	0.22	0.24	0.09
	Adj. R^2	6.23%	-0.43%	6.63%	11.26%	2.89%	-8.17%
Restrictions on public events	b	-0.32	-0.42	-0.42	-0.42	-0.37	-0.32
	SE	(0.27)	(0.29)	(0.29)	(0.31)	(0.32)	(0.43)
	p-value	0.24	0.17	0.17	0.19	0.26	0.46
	exp(b)	0.72	0.66	0.66	0.66	0.69	0.72
	tau^2	0.18	0.21	0.20	0.24	0.24	0.08
	Adj. R^2	2.06%	5.65%	5.11%	5.78%	2.02%	0.27%
Restrictions on private gatherings	b	-0.05	-0.03	-0.12	-0.16	-0.18	-0.14
	SE	(0.09)	(0.13)	(0.09)	(0.13)	(0.13)	(0.11)
	p-value	0.61	0.845	0.191	0.225	0.198	0.195
	exp(b)	0.96	0.98	0.88	0.85	0.84	0.87
	tau^2	0.19	0.23	0.21	0.24	0.24	0.07
	Adj. R^2	-3.42%	-5.81%	4.38%	3.97%	4.29%	10.94%
Closing public transport	b	-0.22	-0.21	-0.31	-0.32	-0.33	-0.10
	SE	(0.12)	(0.15)	(0.12)	(0.18)	(0.14)	(0.15)
	p-value	0.10	0.19	0.02	0.10	0.03	0.51
	exp(b)	0.81	0.81	0.73	0.73	0.72	0.91
	tau^2	0.17	0.21	0.17	0.22	0.19	0.09
	Adj. R^2	8.89%	5.93%	21.22%	12.82%	21.64%	-11.01%
Restrictions on internal movement	b	-0.02	-0.23	-0.26	-0.06	-0.18	-0.37
	SE	(0.21)	(0.24)	(0.22)	(0.28)	(0.26)	(0.23)
	p-value	0.93	0.35	0.24	0.83	0.50	0.13
	exp(b)	0.98	0.80	0.77	0.94	0.84	0.69
	tau^2	0.20	0.22	0.21	0.27	0.26	0.08
	Adj. R^2	-4.77%	-1.30%	1.52%	-6.84%	-3.18%	6.82%
Overall stringency index	b	-0.01	-0.02	-0.02	-0.02	-0.02	-0.01
	SE	(0.01)	(0.01)	(0.01)	(0.01)	(0.01)	(0.01)
	p-value	0.05	0.02	0.001	0.03	0.003	0.10
	exp(b)	0.99	0.98	0.98	0.98	0.98	0.99
	tau^2	0.16	0.16	0.13	0.19	0.15	0.06
	Adj. R^2	12.87%	26.41%	40.18%	24.72%	38.54%	25.53%
Economic support index	b	-0.004	-0.002	-0.01	-0.01	-0.003	-0.003
	SE	(0.004)	(0.01)	(0.004)	(0.01)	(0.01)	(0.004)
	p-value	0.40	0.62	0.08	0.24	0.56	0.46
	exp(b)	1.00	1.00	0.99	0.99	1.00	1.00
	tau^2	0.19	0.23	0.19	0.24	0.26	0.08
	Adj. R^2	-1.17%	-4.86%	9.65%	3.39%	-3.76%	0.78%
	N	23	20	24	16	20	21

Supplementary Table 21. Meta-regression results for the stringency of containment policies, overall stringency index, and economic index on the size of the effect of stay at home orders on crime. Exp(b) reflects the standardized exponentiated coefficient. Adj. (adjusted) R^2 reflects the proportion of variance in the effect sizes explained by the given covariate.

In essence, we found that the stringency of other containment policies was generally not significantly associated with the size of the effects on crime, with two exceptions. More severe restrictions on public transportation (i.e. reducing services, prohibiting most citizens from using it) are associated with a stronger negative effect on vehicle theft (*b*=-0.33, SE=0.14, p=.03) and robbery (*b*=-0.31, SE=0.12, p=.02). Restrictions on public transportation account for 21.64% of the variation in effect sizes for vehicle theft and 21.22% of the variation in effect sizes for robbery. Restricting or prohibiting the use of public transportation may have led to more people using their vehicle to commute to work, where possible. Alternatively, the reduction in the use of public transportation may have also led to fewer suitable targets and opportunities for vehicle theft in public transportation parking lots.

The results for the overall stringency index are relatively similar to those reported in the main text in Table 2 for stay at home restrictions. For assault, the proportion of variation explained by the stringency index remains largely similar to the stay at home restrictions (12.49% for stay at home restrictions vs. 12.87% for overall stringency index). Similarly, for robbery, theft, and homicide the proportion of variance explained by the stringency index is similar to the stay at home models. This suggests that accounting for the stringency of the overall combined policy response does not substantially improve the model fit.

By contrast, for burglary the proportion of variance in effect sizes explained by the stringency index is relatively lower than the stay at home restrictions (26.41% vs. 34.42% respectively). This suggests that accounting for broader policy responses provides a worse fit, and explains slightly less variation in the effect sizes for burglary compared to stay at home restrictions. By contrast, the model fit for vehicle theft marginally increased when using the stringency index instead of stay at home restrictions (38.54% vs. 28.67% respectively).

Finally, we estimated the association between changes in mobility indices, as measured by the Google COVID-19 Community Mobility Reports, and effect sizes for each crime type. More negative, smaller absolute values for the mobility measures indicate larger declines in visits and length of stay at given locations compared to a baseline established in January-February 2020.

		Assault	Burglary	Robbery	Theft	Vehicle theft	Homicide
Retail and recreation	b	0.01	0.01	0.02	0.02	0.02	0.004
	SE	(0.004)	(0.01)	(0.01)	(0.01)	(0.01)	(0.01)
	p-value	0.01	0.11	0.003	0.01	0.01	0.59
	95% CI	(0.004, 0.02)	(-0.003, 0.02)	(0.01, 0.03)	(0.01, 0.03)	(0.005, 0.03)	(-0.01, 0.02)
	exp(b)	1.01	1.01	1.02	1.02	1.02	1.00
	tau^2	0.14	0.20	0.14	0.15	0.17	0.08
	Adj. R^2	26.61%	11.18%	34.52%	40.91%	29.96%	-0.83%
Grocery and pharmacy	b	0.02	0.02	0.03	0.02	0.02	0.01
	se	(0.01)	(0.01)	(0.004)	(0.01)	(0.01)	(0.01)
	p-value	<0.001	0.01	<0.001	0.003	0.01	0.04
	95% CI	(0.01, 0.03)	(0.01, 0.03)	(0.02, 0.04)	(0.01, 0.03)	0.01, 0.03)	(0.001, 0.03)
	exp(b)	1.02	1.02	1.03	1.02	1.03	1.01
	tau^2	0.09	0.14	0.08	0.14	0.16	0.04
	Adj. R^2	52.05%	35.81%	64.52%	44.95%	36.29%	45.63%
Parks	b	0.01	0.01	0.01	0.01	0.01	0.003
	SE	(0.001)	(0.002)	(0.002)	(0.002)	(0.002)	(0.002)
	p-value	0.003	0.01	0.001	0.003	0.003	0.21

	95% CI	(0.002, 0.01)	(0.002, 0.01)	(0.003, 0.01)	(0.003, 0.01)	(0.003, 0.01)	(-0.002, 0.01)
	exp(b)	1.01	1.01	1.01	1.01	1.01	1.00
	tau^2	0.13	0.14	0.13	0.14	0.15	0.06
	Adj. R^2	33.09%	36.21%	38.65%	43.70%	39.01%	20.10%
Transit stations	b	0.01	0.02	0.03	0.02	0.02	0.01
	SE	(0.01)	(0.01)	(0.01)	(0.01)	(0.01)	(0.01)
	p-value	0.02	0.09	<0.001	0.04	0.07	0.10
	95% CI	(0.003, 0.03)	(-0.003, 0.04)	(0.02, 0.04)	(0.001, 0.04)	(-0.002, 0.04)	(-0.003, 0.03)
	exp(b)	1.02	1.02	1.03	1.02	1.03	1.01
	tau^2	0.15	0.20	0.11	0.19	0.22	0.05
	Adj. R^2	20.98%	11.37%	49.53%	22.15%	13.07%	40.86%
Workplace	b	0.01	0.01	0.02	0.02	0.01	0.01
	SE	(0.01)	(0.01)	(0.01)	(0.01)	(0.01)	(0.01)
	p-value	0.05	0.59	0.04	0.04	0.40	0.56
	95% CI	(0.00002, 0.03)	(-0.02, 0.03)	(0.001, 0.04)	(0.001, 0.04)	(-0.01, 0.03)	(-0.01, 0.02)
	exp(b)	1.02	1.01	1.02	1.02	1.02	1.01
	tau^2	0.16	0.23	0.18	0.20	0.25	0.08
	Adj. R^2	13.26%	-3.92%	16.01%	21.42%	-1.53%	-0.94%
Residential	b	-0.04	-0.03	-0.06	-0.05	-0.04	-0.03
	SE	(0.02)	(0.02)	(0.01)	(0.02)	(0.02)	(0.02)
	p-value	0.01	0.08	<0.001	0.01	0.02	0.07
	95% CI	(-0.06, -0.01)	(-0.07, 0.004)	(-0.08, -0.03)	(-0.08, -0.02)	(-0.08, -0.01)	(-0.07, 0.003)
	exp(b)	0.95	0.97	0.95	0.95	0.94	0.97
	tau^2	0.14	0.19	0.11	0.15	0.19	0.05
	Adj. R^2	26.94%	12.55%	47.74%	40.06%	23.35%	43.28%
	N	23	20	24	16	20	21

Supplementary Table 22. Meta-regression results for Google COVID-19 Community Mobility Report measures on the size of the effect of stay at home orders on crime. 95% CI=95% confidence intervals. Exp(b) reflects the standardized exponentiated coefficient. Adj. (adjusted) R^2 reflects the proportion of variance in the effect sizes explained by the given covariate. SD=standard deviation. Mobility measures reflect percentage change in visits and length of stay in a given location relative to a baseline established in January-February 2020. Note that due to differences in categorization of locations between regions and countries, Google does not recommend the use of mobility data to make comparisons across countries. We therefore urge caution in interpreting these results.

Although we must consider the mobility data with caution due to issues with comparability across countries, the results in Supplementary Table 22 are generally in line with the results using the stringency of stay at home measures (see Table 2). Cities that saw greater declines in the use of public space, in particular retail and recreation, groceries and pharmacies, and parks experiences larger declines in crime, with the exception of homicide. For example, Supplementary Figure 27 contains the scatterplots displaying the association between estimated effect size and the percentage change in retail and recreation mobility. The differential results for homicide may reflect differences in the operationalization and measurement of stay at home restrictions and their effects. Lima, one of the few cities that experienced a significant decline in homicides, has one of the most stringent stay at home policies according to OxCGRT coding, whereas the percentage decline in retail and recreation mobility is relatively on par with cities that did not experience such declines (e.g. London, Amsterdam). In addition, the actual change in mobility may be imperfectly captured by the Google reports due to differences in categorization of locations across regions.

2.6 Residential and commercial burglaries

It is important to note that the majority of cities in our sample do not distinguish between residential and commercial burglaries, whereas the opportunities for these crimes may have been differentially affected by stay at home restrictions. With more people at home, and fewer houses left empty during the day, the number of suitable targets for residential burglary likely declined. While more commercial properties may have been empty during this same period, it is not clear to what extent motivated offenders may have substituted commercial for residential targets. The number of suitable commercial targets may have increased, however the reduction in movement in commercial areas coupled with potential increases in policing might mean that the risk of detection is higher than usual. It was possible to examine to what extent these trends differ in a subset of cities in our sample: Barcelona, Vancouver, San Francisco, and London. Specifically, we plotted the number of residential and commercial burglaries per day starting on January 1st, 2020. The results suggest that the number of residential burglaries declined following the implementation of stay at home restrictions, whereas commercial burglaries remained unaffected (Supplementary Figure 28). In Vancouver, there was a slight increase in the number of commercial burglaries during the lockdown period, which might suggest substitution from residential to commercial properties. However, in the other three cities, there appears to be little to no substitution from residential to commercial properties.

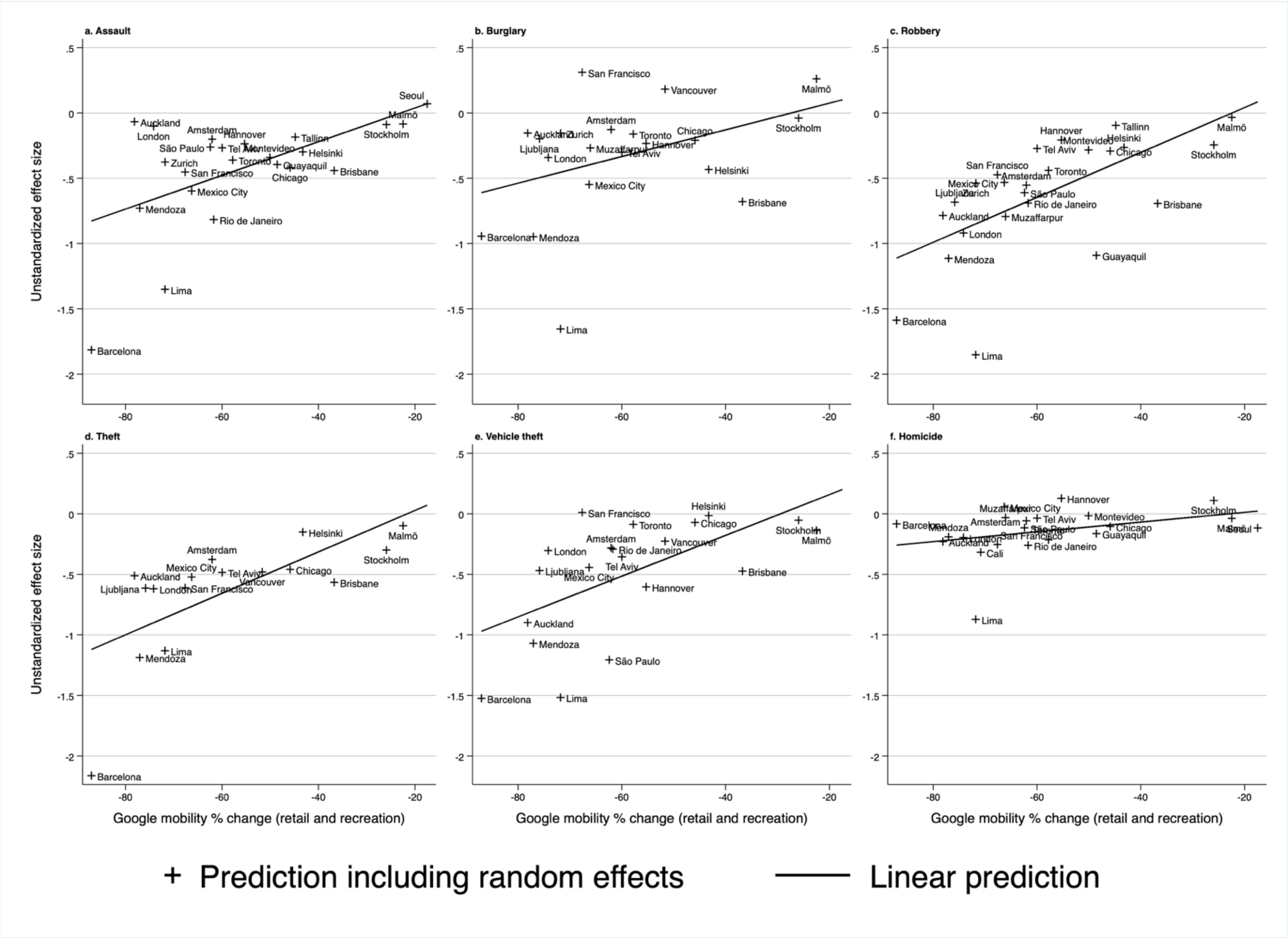

Supplementary Figure 27. Scatterplots of predicted effect size including random effects against changes in retail and recreation mobility. Estimated predictions are drawn from meta-regression analyses. Mobility measures reflect percentage change in visits and length of stay in a given location relative to a baseline established in January-February 2020. Note that due to differences in categorization of locations between regions and countries, Google does not recommend the use of mobility data to make comparisons across countries. We therefore urge caution in interpreting these results.

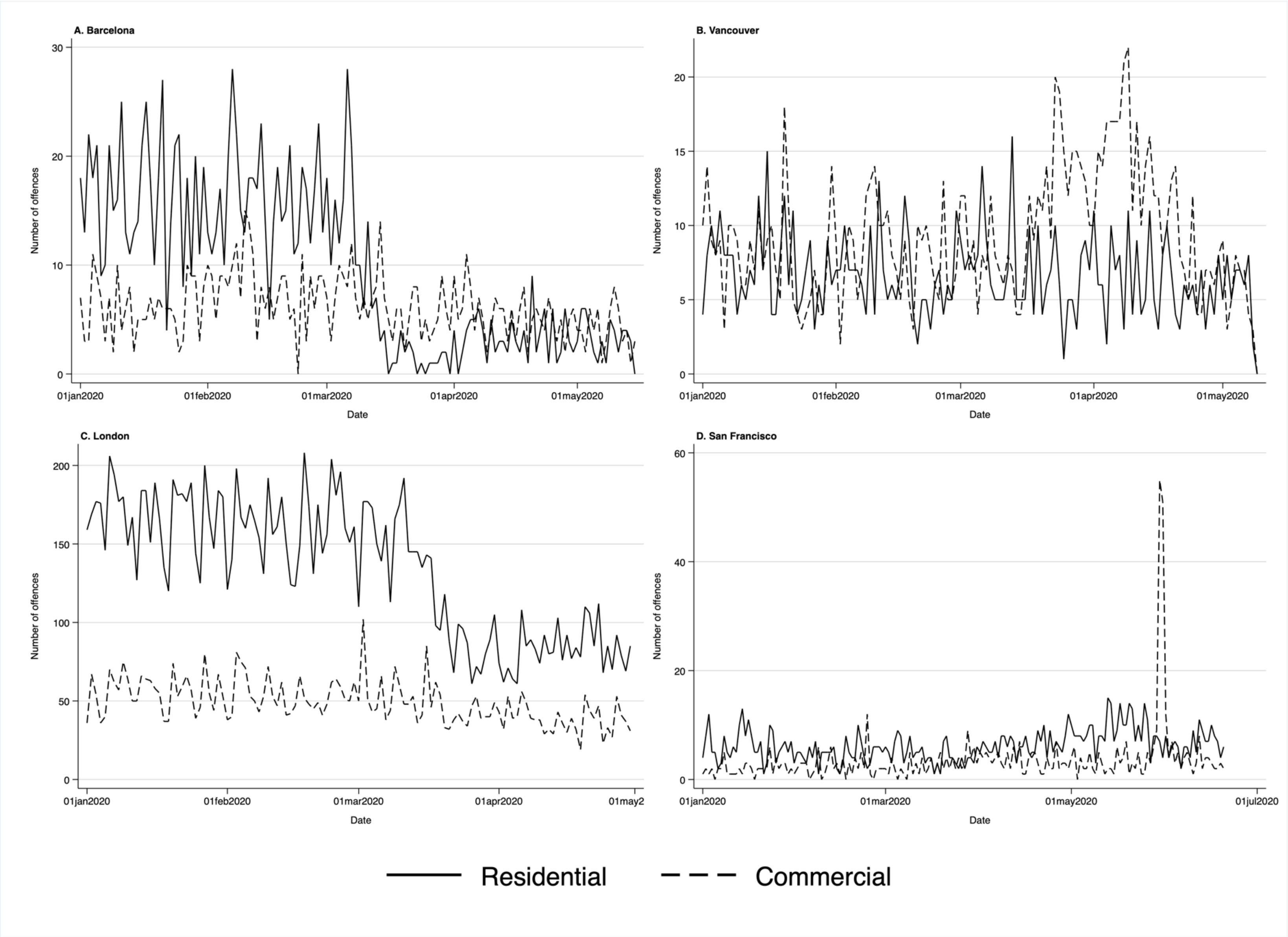

Supplementary Figure 28. Time series plots of residential and commercial burglary for four cities.

Supplementary References

1. New Zealand Police. Daily occurences of crime and family violence investigations https://www.police.govt.nz/about-us/statistics-and-publications/data-and-statistics/daily-occurrences-crime (New Zealand Police, 2020).
2. Queensland Police Service. Queensland Police Service – Online Crime Map https://qps-ocm.s3-ap-southeast-2.amazonaws.com/index.html (Queensland Police Service, 2020).
3. Alcaldía de Santiago de Cali. Observatorio de Seguridad https://www.cali.gov.co/observatorios/publicaciones/147466/observatorio_seguridad/ (Alcaldía de Santiago de Cali, 2020).
4. Chicago Data Portal. *Crimes – 2001 to Present*. https://data.cityofchicago.org/Public-Safety/Crimes-2001-to-Present/ijzp-q8t2 (2020).
5. DataMendoza. Análisis de delitos seleccionados para el Centro de Estudios Latinoamericanos sobre Inseguridad y Violencia. Report, May 2020 (Ministerio de Seguridad, 2020).
6. DataCDMX. Portal de Datos Abiertos de la Ciudad de Mexico (CDMX) https://datos.cdmx.gob.mx/dataset/victimas-en-carpetas-de-investigacion-fgj (Gobierno de la Ciudad de Mexico, 2020).
7. State Crimes Records Bureau Bihar, [No Title] http://scrb.bihar.gov.in/View_FIR.aspx (2020).
8. DataSF. *Police Department Incident Reports: 2018 to Present* https://data.sfgov.org/Public-Safety/Police-Department-Incident-Reports-2018-to-Present/wg3w-h783 (San Francisco Police Department, 2020).
9. Toronto Police Service. *Public Safety Data Portal* https://data.torontopolice.on.ca/search?q=crime *(*2020).
10. Vancouver Police Department. *Crime Data Download* https://geodash.vpd.ca/opendata/ (2020).
11. Bisogno, E., Dawson-Faber, J. & Jandl, M. The International Classification of Crime for Statistical Purposes: A new instrument to improve comparative criminological research. *Eur J Criminol* **12**, 535-550 (2015).
12. NOS Nieuws. *Nieuwe Corona-maatregelen: Dit Betekenen ze Voor Jou* https://nos.nl/artikel/2326888-nieuwe-corona-maatregelen-dit-betekenen-ze-voor-jou.html (2020).
13. *New Zealand Government Covid-19 Alert System* https://covid19.govt.nz/alert-system/alert-system-overview/ (2020).
14. Gobierno de España. *Real Decreto 463/2020, de 14 de marzo, por el que se declara el estado de alarma para la gestión de la situación de crisis sanitaria ocasionada por el COVID-19* https://www.boe.es/diario_boe/txt.php?id=BOE-A-2020-3692 (2020).
15. Queensland Health. *Queensland novel coronavirus (COVID-19) update: 29 March 2020* https://www.health.qld.gov.au/news-events/doh-media-releases/releases/queensland-novel-coronavirus-covid-19-update15 (2020).
16. AS. *¿Cuántos casos de coronavirus hay en Colombia a día 24 de marzo?* https://colombia.as.com/colombia/2020/03/24/tikitakas/1585069534_002011.html (2020).
17. Hale, T., et al. A global panel database of pandemic policies (Oxford COVID-19 government response tracker). *Nat Hum Behav*, https://doi.org/10.1038/s41562-021-01079-8 (2021).

18. El Comercio. *Coronavirus en Ecuador: Un ABC de la cuarentena, restricciones,* teletrabajo https://www.elcomercio.com/actualidad/coronavirus-cuarentena-ecuador-teletrabajo-salvoconductos.html (2020).
19. El Comercio, *El COE de Guayaquil endurece medidas por el aumento de casos de covid-19; las UCI están al 95%* https://www.elcomercio.com/actualidad/restriccion-bebidas-alcoholicas-guayaquil-covid19.html (2020).
20. El Universo. *Qué implica el final del estado de excepción en Ecuador* https://www.eluniverso.com/noticias/2020/08/26/nota/7955587/que-implica-final-estado-excepcion-ecuador (2020).
21. Niedersächsisches Ministerium für Soziales Gesundheit und Gleichstellung. *Maßnahmen im Kampf gegen Covid-19: Land untersagt alle öffentlichen Veranstaltungen - Schließung aller Freizeit- und Kultureinrichtungen und Teile des Einzelhandels* https://www.ms.niedersachsen.de/startseite/service_kontakt/presseinformationen/massnahmen-im-kampf-gegen-covid-19-land-untersagt-alle-offentlichen-veranstaltungen-schliessung-aller-freizeit-und-kultureinrichtungen-und-teile-des-einzelhandels-186324.html (2020),
22. Finnish Government. *Changes to Restrictions Imposed due to COVID-19 Epidemic as of 1 June* https://valtioneuvosto.fi/en/-/10616/muutoksia-koronavirusepidemian-vuoksi-asetettuihin-rajoituksiin-1-kesakuuta (2020).
23. YLE. *Coronavirus Updates Archive: 10 March - 10 April 2020* https://yle.fi/uutiset/osasto/news/coronavirus_updates_archive_10_march_-_10_april_2020/11307944 (Finnish Broadcasting Company, 2020).
24. Wikipedia España. *Pandemia de COVID-19 en Perú* https://es.wikipedia.org/wiki/Pandemia_de_COVID-19_en_Perú (2020).
25. European Centre for Disease Prevention and Control. *COVID-19 Country Overviews* https://covid19-country-overviews.ecdc.europa.eu/#31_slovenia (2020).
26. Republic of Slovenia. *Ordinance on the Temporary Prohibition of Public Gathering at Public Meetings and Public Events and Other Events in Public Places in the Republic of Slovenia* https://www.gov.si/en/news/2020-03-19-ordinance-on-the-temporary-prohibition-of-public-gathering-at-public-meetings-and-public-events-and-other-events-in-public-places-in-the-republic-of-slovenia/ (2020).
27. The Health Foundation. *COVID-19 Policy Tracker* https://www.health.org.uk/news-and-comment/charts-and-infographics/covid-19-policy-tracker (2020).
28. Wikipedia English. *Swedish Government Response to the COVID-19 Pandemic* https://en.wikipedia.org/wiki/Swedish_government_response_to_the_COVID-19_pandemic (2020).
29. Folkhälsomyndigheten. *Covid-19* https://www.folkhalsomyndigheten.se/smittskydd-beredskap/utbrott/aktuella-utbrott/covid-19/ (2020).
30. Gobierno de México. *Se declara como emergencia sanitaria la epidemia generada por COVID-19* https://www.gob.mx/cjef/documentos/se-declara-como-emergencia-sanitaria-la-epidemia-generada-por-covid-19?idiom=es (2020).
31. Wikipedia English. *COVID-19 Pandemic in Uruguay* https://en.wikipedia.org/wiki/COVID-19_pandemic_in_Uruguay (2020).
32. República Oriental del Uruguay - Ministerio de Economía y Finanzas [*Powerpoint*] https://www.gub.uy/ministerio-economia-finanzas/sites/ministerio-economia-finanzas/files/documentos/noticias/Uruguay_Institutional%20Presentation_July%202020%20AS-COA%20Final.pdf (2020).

33. Taylor, L. Uruguay is winning against Covid-19. This is how. *BMJ* ***370,*** 1-2. (2020).
34. Poblete-Cazenave, R. The Great Lockdown and criminal activity: Evidence from Bihar, India. *Covid Economics* **29,** 141-163 (2020).
35. Procuradoria Geral. *Diario Oficial Do Estado Do Rio de Janeiro* https://pge.rj.gov.br/comum/code/MostrarArquivo.php?C=MTAyMjE%2C (2020).
36. Prefeitura Da Cidado Do Rio de Janeiro. *Leis Municipais, Decretos, Resoluções e Portarias* http://www.rio.rj.gov.br/web/transparencia/legislacao-coronavirus (2020).
37. Câmara Municipal de São Paulo. *Informação No Combate Ao Coronavírus* http://www.saopaulo.sp.leg.br/coronavirus/leis/ (2020).
38. Center for Strategic and International Studies. *A Timeline of South Korea's Response to COVID-19* https://www.csis.org/analysis/timeline-south-koreas-response-covid-19/ (2020).
39. Wikipedia English. *COVID-19 Pandemic in South Korea* https://en.wikipedia.org/wiki/COVID-19_pandemic_in_South_Korea#cite_note-:0-97 (2020).
40. Vabariigi Valitsuse. *Koroonakriis Eestis* https://www.valitsus.ee/et/eriolukord-eestis (2020).
41. State of Israel. *The Novel Coronavirus* https://govextra.gov.il/ministry-of-health/corona/corona-virus-en/ (2020).
42. National Post. *COVID-19: Ontario and Quebec Order Non-essential Businesses Closed after Spike in Coronavirus Totals* https://nationalpost.com/news/covid-19-ontario-and-quebec-order-non-essential-businesses-closed-after-spike-in-coronavirus-totals (2020).
43. Vancouver Sun. *COVID-19: B.C. Officials Declare Public Health Emergency as 83 New Cases Announced* https://vancouversun.com/health/covid-19-bc-officials-to-update-case-count (2020).
44. City of Vancouver. *Stay Home, Stay Put and Help Flatten Vancouver's Curve* https://vancouver.ca/news-calendar/stay-home-stay-put-and-help-flatten-vancouvers-curve.aspx (2020).
45. Swiss Confederation. *Coronavirus: Bundesrat erklärt die «ausserordentliche Lage» und verschärft die Massnahmen* https://www.admin.ch/gov/de/start/dokumentation/medienmitteilungen.msg-id-78454.html (2020).
46. GitHub. *Codebook for the Oxford Covid-19 Government Response Tracker* https://github.com/OxCGRT/covid-policy-tracker/blob/master/documentation/codebook.md (2020).
47. GitHub. *Methodology for Calculating Indices* https://github.com/OxCGRT/covid-policy tracker/blob/master/documentation/index_methodology.md (2020).
48. Google, Inc. *Google COVID-19 Community Mobility Reports* https://www.google.com/covid19/mobility/index.html?hl=en (2020).
49. Google, Inc. *Mobility Report CSV Documentation* https://www.google.com/covid19/mobility/data_documentation.html?hl=en (2020).
50. Bhaskaran, K., Gasparrini, A., Hajat, S., Smeeth, L. & Armstrong, B. Time series regression studies in environmental epidemiology. *Int J Epidemiol* **42**, 1187–1195 (2013).
51. MacDonald, J.M. & Lattimore, P.K. Count Models in Criminology. in *Handbook of Quantitative Criminology*, (eds. Piquero, A.D. & Weisburd, D.) 683–698 (Springer, 2010).
