# Supplementary material for: A global analysis of the impact of COVID-19 stay-at-home restrictions on crime
Source: Nat Hum Behav. 2021 Jun 2;5(7):868–77. doi: 10.1038/s41562-021-01139-z (PMC8298205; doi:10.1038/s41562-021-01139-z)
Supplement: Supplementary file 2 — Reporting summary [file 41562_2021_1139_MOESM2_ESM.pdf]

nature research

Corresponding author(s): Nivette, Amy

Last updated by author(s): May 3, 2021

Reporting Summary

Nature Research wishes to improve the reproducibility of the work that we publish. This form provides structure for consistency and transparency in reporting. For further information on Nature Research policies, see our Editorial Policies and the Editorial Policy Checklist.

Statistics

For all statistical analyses, confirm that the following items are present in the figure legend, table legend, main text, or Methods section.

n/a	Confirmed	
☒	☐	The exact sample size (*n*) for each experimental group/condition, given as a discrete number and unit of measurement
☒	☐	A statement on whether measurements were taken from distinct samples or whether the same sample was measured repeatedly
☐	☒	The statistical test(s) used AND whether they are one- or two-sided<br>*Only common tests should be described solely by name; describe more complex techniques in the Methods section.*
☐	☒	A description of all covariates tested
☐	☒	A description of any assumptions or corrections, such as tests of normality and adjustment for multiple comparisons
☐	☒	A full description of the statistical parameters including central tendency (e.g. means) or other basic estimates (e.g. regression coefficient) AND variation (e.g. standard deviation) or associated estimates of uncertainty (e.g. confidence intervals)
☐	☒	For null hypothesis testing, the test statistic (e.g. *F*, *t*, *r*) with confidence intervals, effect sizes, degrees of freedom and *P* value noted<br>*Give P values as exact values whenever suitable.*
☒	☐	For Bayesian analysis, information on the choice of priors and Markov chain Monte Carlo settings
☐	☒	For hierarchical and complex designs, identification of the appropriate level for tests and full reporting of outcomes
☐	☒	Estimates of effect sizes (e.g. Cohen's *d*, Pearson's *r*), indicating how they were calculated

Our web collection on statistics for biologists contains articles on many of the points above.

Software and code

Policy information about availability of computer code

Data collection	No custom code was used for data collection.
Data analysis	All statistical code for analyses was created in Stata 16.

For manuscripts utilizing custom algorithms or software that are central to the research but not yet described in published literature, software must be made available to editors and reviewers. We strongly encourage code deposition in a community repository (e.g. GitHub). See the Nature Research guidelines for submitting code & software for further information.

Data

Policy information about availability of data

All manuscripts must include a data availability statement. This statement should provide the following information, where applicable:

- Accession codes, unique identifiers, or web links for publicly available datasets
- A list of figures that have associated raw data
- A description of any restrictions on data availability

All data, code, and materials used in the analyses have been deposited in the data repository Data Archiving and Networked Services [DANS] for purposes of reproducing or extending the analysis.

Field-specific reporting

Please select the one below that is the best fit for your research. If you are not sure, read the appropriate sections before making your selection.

☐ Life sciences ☒ Behavioural & social sciences ☐ Ecological, evolutionary & environmental sciences

For a reference copy of the document with all sections, see nature.com/documents/nr-reporting-summary-flat.pdf

Behavioural & social sciences study design

All studies must disclose on these points even when the disclosure is negative.

Study description	The stay at home restrictions to control the spread of COVID-19 led to unparalleled sudden change in daily life, but it is unclear how they affected urban crime globally. We collected data on daily counts of crime in 26 cities across 22 countries in the Americas, Europe, the Middle East and Asia. We conducted interrupted time series analyses to assess the impact of stay at home restrictions on different types of crime in each city. Our findings show that the stay at home policies were associated with a considerable drop in urban crime, but with substantial variation across cities and types of crime. Meta-regression results showed that more stringent restrictions over movement in public space were predictive of larger declines in crime.
Research sample	Daily crime data were collected from a convenience sample of 26 cities representing 22 countries from around the globe. The cities were selected in an attempt to maximize geographical coverage and capture a range of policy responses aimed to reduce the transmission and spread of COVID-19.
Sampling strategy	The study is based on a convenience sample of 26 cities. The target sample of this study were cities that represent different parts of the world and that have an incident-base database of computerised daily counts of police recorded crime since at least the 1 January 2019. In many countries, access to police records of daily incident counts is only granted to trusted academics known to police forces. We therefore contacted colleagues across the world with a track record of working with local police data and a good understanding of the local data generation processes. In some cases data could not be contributed because no electronic databases with daily counts exist or because the authorities did not grant permission to access the data. The strategy resulted in a good representation of cities in North America, Western and Eastern Europe, South America, and Oceania. All attempts to include a city in Africa were unsuccessful (mainly because no such data exist), and coverage of Asia is patchy. Within each city all cases of police recorded crime within our defined crime categories are used.
Data collection	For each city, we sought the number of police-recorded offences for assault, burglary, robbery, theft, motor vehicle theft, and homicide. Since crime categories and definitions were likely to deviate across countries and cities, we utilized the International Classification of Crime for Statistical Purposes to ensure that crime categories were as comparable as possible.
Timing	Data were collected beginning in the second half of 2020. For most cities, the time series starts on 1 January 2018 or 2019, and ends on the most recent date available. Time series information for all cities is reported in Supplementary Table 10.
Data exclusions	Not all crime categories were available for each city, and in some contexts certain crimes are not treated as separate categories or do not fit the pre-determined ICCS definitions. For example, in Seoul burglary is not considered separately from robbery, and motor vehicle theft is not distinguished from theft. In order to ensure that the crime categories are as comparable as possible, we have excluded these combined outcomes from the analyses.
Non-participation	Not applicable.
Randomization	Not applicable.

Reporting for specific materials, systems and methods

We require information from authors about some types of materials, experimental systems and methods used in many studies. Here, indicate whether each material, system or method listed is relevant to your study. If you are not sure if a list item applies to your research, read the appropriate section before selecting a response.

Materials & experimental systems

n/a	Involved in the study	
☒	☐	Antibodies
☒	☐	Eukaryotic cell lines
☒	☐	Palaeontology and archaeology
☒	☐	Animals and other organisms
☒	☐	Human research participants
☒	☐	Clinical data
☒	☐	Dual use research of concern

Methods

n/a	Involved in the study	
☒	☐	ChIP-seq
☒	☐	Flow cytometry
☒	☐	MRI-based neuroimaging
