# Supplementary material for: A global analysis of the impact of COVID-19 stay-at-home restrictions on crime
Source: Nat Hum Behav. 2021 Jun 2;5(7):868–77. doi: 10.1038/s41562-021-01139-z (PMC8298205; doi:10.1038/s41562-021-01139-z)
Supplement: Supplementary file 3 — Peer review information [file 41562_2021_1139_MOESM3_ESM.pdf]

Peer Review Information

Journal: Nature Human Behaviour
Manuscript Title: A global analysis of the impact of COVID-19 stay at home restrictions on crime
Corresponding author name(s): Amy E. Nivette

Editorial Notes:

Reviewer Comments & Decisions:

Decision Letter, initial version:

10th February 2021

Dear Dr. Nivette,

Thank you once again for your manuscript, entitled "The impact of COVID-19 stay at home restrictions on crime: A global analysis," and for your patience during the peer review process.

Your manuscript has now been evaluated by 3 reviewers, whose comments are included at the end of this letter. Although the reviewers find your work to be of interest, they also raise some important concerns. We are very interested in the possibility of publishing your study in Nature Human Behaviour, but would like to consider your response to these concerns in the form of a revised manuscript before we make a decision on publication.

To guide the scope of the revisions, the editors discuss the referee reports in detail within the team, including with the chief editor, with a view to (1) identifying key priorities that should be addressed in revision and (2) overruling referee requests that are deemed beyond the scope of the current study. We hope that you will find the prioritised set of referee points to be useful when revising your study. Please do not hesitate to get in touch if you would like to discuss these issues further.

In particular, your revision must address the following (as well as all other reviewer comments):

1. Ensure that the models used are clearly presented and fully justified. The reviewers highlight several modeling choices that will require additional discussion, such as the choice of a Poisson model and linear vs. quadratic models.

2. Address concerns and questions raised by all 3 reviewers about the severity and stringency indices used.

3. Ensure that the definition and measurement of all variables are clear, and discuss any relevant variation across locations. Please also ensure that your Supplementary Information includes full details on the rationale behind your initial sampling strategy (e.g. how sites were chosen and what strategy you used to maximise geographical coverage and capture a range of policy responses).

4. Address concerns raised by Reviewer 3 about multiple hypothesis testing.

5. Include a detailed discussion of limitations of this work, as requested by Reviewer 1. Please discuss here the implications of the categories of crime covered by your data, and discuss explicitly the extent to which your data speak to concerns about displacement and rises in domestic violence and drug-related crime.

Finally, your revised manuscript must comply fully with our editorial policies and formatting requirements. Failure to do so will result in your manuscript being returned to you, which will delay its consideration. To assist you in this process, I have attached a checklist that lists all of our requirements. If you have any questions about any of our policies or formatting, please don't hesitate to contact me.

In sum, we invite you to revise your manuscript taking into account all reviewer and editor comments. We are committed to providing a fair and constructive peer-review process. Do not hesitate to contact us if there are specific requests from the reviewers that you believe are technically impossible or unlikely to yield a meaningful outcome.

To ensure timely potential dissemination, we hope to receive your revised manuscript within four to six weeks. Please do let me know whether this revision timeframe is feasible or whether you would envision the revision process taking longer.

With your revision, please:

• Include a “Response to the editors and reviewers” document detailing, point-by-point, how you addressed each editor and referee comment. If no action was taken to address a point, you must provide a compelling argument. This response will be used by the editors to evaluate your revision and sent back to the reviewers along with the revised manuscript.

• Highlight all changes made to your manuscript or provide us with a version that tracks changes.

Please use the link below to submit your revised manuscript and related files:

[REDACTED]

Note: This URL links to your confidential home page and associated information about manuscripts you may have submitted, or that you are reviewing for us. If you wish to forward this email to co-authors, please delete the link to your homepage.

We look forward to seeing the revised manuscript and thank you for the opportunity to review your work. Please do not hesitate to contact me if you have any questions or would like to discuss these revisions further.

Sincerely,
Aisha

Aisha Bradshaw
Editor
Nature Human Behaviour

Reviewer expertise:

Reviewer #1: criminology

Reviewer #2: mathematics, analysis of crime

Reviewer #3: criminology

REVIEWER COMMENTS:

Reviewer #1:
Remarks to the Author:
In this study, the authors aim at performing a globally-inspired analysis of the effects of C19 on crime across the globe. To my knowledge, this study is the first truly globally-informed analysis on how C19 has impacted crime across the planet.

I commend the authors for carefully executing what can only be described as a fantastic study. Before reviewing how this study could be improved, I will begin by reviewing what I believe are two unique strengths of this paper.

Strength 1: This paper's analysis is truly global. Up to this point in the research, studies have focused on either 1) individual cities, 2) groups of cities in the same country, and/or 3) no data at all. While I do understand that the 25 cities in this analysis are not randomly selected and hardly represent the 'total' global picture of crime, this study's purposes and goals are admirable. In short, this took a ton of work to achieve: I commend the authors for organizing such a herculean effort.

Strength 2: The findings offer new insight that is highly important. Before this article, the findings were relatively mixed as to whether there was a notable crime reduction across the globe (and in local

populations, as well). The main finding prior to this paper is that there was a crime drop, but it seems like it is highly geographically situational and also dependent on the type of crime in question. With this study, we now have evidence why: The crime reductions – in one of the main findings of this paper – were temporary. The authors clearly lay out that the crime drops during lockdowns were temporary and the baseline levels were reached after a few months. Furthermore, for the most serious crime in question in this study – homicide – the drops were minimal. The findings here paint a picture that significantly advances the research on C19 and crime across the globe in both broad, sweeping ways as well as in more nuanced manners.

With this stated, I do have some recommendations for ways that the study could be improved. I review these things in the remainder of this review. First, one of the most confusing things in this study is the two measures of C19 response severity: The overall stringency index and the severity index. Please include extra information on how both of these measures were created. Additionally, I noticed that there is a very substantial correlation between these two measures (r = .84). With this very strong correlation, I have two questions. First, is there really anything to be gained by analyzing both measures? Could this study be simplified by only presenting the findings from one of these measures? Second, I have a question about how these variables are being used in analysis. In Table 1, are both measures used in the same analysis? The reason why I ask is because it is highly unusual that the coefficient values of one of the two measures (overall stringency) is minimal. The coefficients of the other measure – stringency of stay at home restrictions – are much stronger in absolute value (I also understand that there are differences in scaling metrics). Since the measures are so highly correlated with each other, shouldn't the models effectively show the same thing? I am concerned here about issues of severe multicollinearity.

Second, I have some questions about definitions of crimes. While things like 'homicide' are relatively timeless and universal in what they mean, things like assault are not. Many places differentiate between assault (as a serious and credible threat to person) and battery (actual physical harm). Burglary can be also combined with robbery in some places (this is dependent on local laws and police practices). Is this captured in the current analysis?

Third, on lines 280-282, the authors talk about crime displacement. This is a very important section of this paper. Can you please explain why displacement was not found? I guess that it makes sense from the perspective that if all crime is decreasing there is not displacement, more on this would be very useful, particularly among extensive speculation about feared increases in domestic and household-based violence.

On lines 290-300, the authors discuss that the co-presence of offenders could have impacted the results. To be clear, co-offending was not captured in the analyses, correct? If this is the case, the authors should consider revising these comments as this analysis is not properly suited to investigate this issue.

Please include a detailed discussion of limitations (e.g., police data; non-random sample; not necessarily representative of everywhere; the dark figure of crime; etc.).

Thank you for a detailed supplemental document. I very much have enjoyed this study and it makes a significant contribution.

Reviewer #2:
Remarks to the Author:
This paper analyzes changes in crime rates in 25 cities in response to stay at home orders associated with the COVID-19 pandemic. The paper uses interrupted time series analysis to show that there were significant crime rate reductions (associated with more stringent public health measures), however variation existed across cities.

There are a number of papers that analyze similar trends in individual cities, however this paper is an important contribution as it provides the first meta analysis. The authors employ rigorous statistical methodology to analyze the drop in crime across cities in the spring of 2020 and contextualize their findings with social and criminological theory in the discussion. Overall I think this is a nice paper worthy of publication.

Below I have some specific comments that I think could improve the manuscript.

1. The authors state the dependent variable is crime “incidents.” This is incorrect, the dependent variable is reported crime incidents. This is an important distinction, as it’s possible that the pandemic led to under- or over-reporting effects not accounted for in the model.

2. Is stringency index the right independent variable to use? There is some evidence that human behavior (e.g. mobility) changes preceded public health measures:

Gupta, Sumedha, et al. Tracking public and private responses to the COVID-19 epidemic: evidence from state and local government actions. No. w27027. National Bureau of Economic Research, 2020.

Maybe using a geolocation based mobility index would provide a better predictor of the crime change response variable.

3. The supplemental material seems a bit long and un-organized. I would prefer the methodological material up front rather than variable definitions for each city. Or, maybe more details on the ITS model could be provided in the main text?

4. I think Figure 1 can be improved with better labeling. It was a little unclear what day 0 is, but I think it is the day stay at home measures were implemented. If this is the case, I think it would be better to have a vertical line with a text label indicating stay at home measures were implemented. I would also include 50-100 days prior (like is done in the supplemental material), because the drop is fairly dramatic when visualized in this way. Also, the y-axis label is confusing since it says number of offenses but they all start at 100. Are the time series normalized or just shifted? This should be stated in the figure.

5. Does the meta-analytic technique in Figure 2 control for multiple hypothesis testing? If so that should be explained in the caption. If not, then the statistical significance should be interpreted accordingly. Same for Figure 3.

6. Why is a quadratic fit used in Figure 4A and linear in 4b? Was some type of model selection criteria

used and if so what was the criteria? Uncertainty in the slope should also be shown in these figures.

Reviewer #3:
Remarks to the Author:
Key results: Please summarise what you consider to be the outstanding features of the work.

The outstanding features of the work include a large-scale, international comparison of crime trends as a result of COVID-19 related social restrictions. These results examines six crime types across 25 cities in 21 countries. This is the largest analysis f the impact of COVID-19 related restrictions on crime to date.

Validity: Does the manuscript have flaws which should prohibit its publication? If so, please provide details.

I don't believe the manuscript has flaws which should prohibit its publication. The research question is clear and concise, the review of the literature is succinct, yet comprehensive, the methods are appropriate, and the findings are straightforward and consistent with much of the recent literature on the subject. The large scale comparison allows for testing of theoretical explanations and the findings have clear policy implications.

Originality and significance: What are the major claims of the paper? Do you think that they represent a significant advance in the field? If the conclusions are not original, please provide relevant references. On a more subjective note, do you feel that the results presented are of immediate interest to many people in your own discipline, and/or to people from several disciplines?

This is a significant paper. There has been a deluge of research on COVID-19, but most of this research is restricted to the city level (save for two articles on state-wide differences in Australia), as far as I'm aware.

Evidence based advance: The study is not completely original in that numerous other studies have examined the impact of COVID-19 on crime, have generally found declines, and have supported opportunity theories to explain these declines. However, this has not been examined at such a scale, and by controlling for severity of restrictions. I believe the paper promises to be an influential piece in the emerging area of COVID-19 and crime and the results presented are of immediate interest to social scientists in general. The study provides an international natural experiment of theories of crime and the findings support understandings of how changes in routine activities at the global scale can affect offending behaviour.

Applied/societal/policy related advance: In addition, the findings have implications for criminal justice agencies, like the police, who are responding to these changes in crime and who may use this information to reallocate resources during subsequent lockdowns or prepare for other pandemics.

Note that Nature Human Behaviour publishes manuscripts that represent a significant advance in one

or more of the following categories:

Conceptual novelty
Methodological novelty
Applied/Societal-/Policy-related Advance
Evidence-based advance [Although a manuscript may lack conceptual novelty, it may represent an evidence-based advance if its scale and/or rigour supersede the existing literature and significantly strengthen confidence in a scientific finding or convincingly falsify it.]

Data & methodology: Please comment on the validity of the approach, quality of the data and quality of presentation. Please note that we expect our reviewers to review all data, including any extended data and supplementary information. Is the reporting of data and methodology sufficiently detailed and transparent to enable reproducing the results?

The authors use interrupted time series analysis to compare daily counts of six types of crime across 25 cities in 21 countries. The methods are appropriate considering the count nature of the data. The authors also account for seasonality, weather and normalized metrics for crime types (including local definitions) and the stringency of social restrictions. This appropriately reflect the shift to routine activities and strain as outlined by the authors. In addition, the selection of a small number of key crime types allows for comparison, but is not overwhelming in terms of interpretation. The reporting of data and methodology is sufficiently detailed and transparent to enable reproducing the results.

Nonetheless, I have a few comments/questions for the authors:
1. Why is Poisson used? It appears that you don't find over-dispersion, but an over-dispersion variable is included. Some clarification is necessary here.
2. The authors make reference to a lot of zero counts (not surprising with daily data or with using homicide) – why not use a zero-inflated model?
3. Is the severity of stay at home restrictions variable entered in the analysis separately? It isn't clear in the description of the data. If it is, does it make sense for this variable to be ordinal? This should at least be tested and reported.
4. An equation showing what was estimated would be useful to answer some of these questions. If I haven't missed it, I would strongly suggest that an equation for the model that is estimated is included.

Preregistration: If any part of the work reported in the manuscript was pre-registered, did the authors follow their preregistration plan? Did they report any deviations from their preregistration? Note that we ask authors to provide a link to the pre-registration in the Methods section and state the date of pre-registration. We also ask that authors disclose all deviations from the pre-registered protocol and explain the rationale for deviation (e.g., flaw, suboptimality, or reviewer/editorial request). In cases of deviation from the analysis plan, the originally planned analyses need to be reported in Supplementary Information.

Appropriate use of statistics and treatment of uncertainties: Please include in your report a specific comment on the appropriateness of any statistical tests, and the accuracy of the description of any error bars and probability values.

Conclusions: Do you find that the conclusions and data interpretation are robust, valid and reliable?

The conclusions are straightforward, robust, valid and reliable. The authors found that more severe restrictions on non-essential movement led to larger declines in crime (with some differences in model fit). These do not lead to changes in effect sizes (save for public transit restrictions and vehicle theft). The researchers also recognize the limitations of not being able to examine cyber crime, which is a common limitation in this research area. Interpretations are consistent with results and with the current findings in the broader literature on COVID-19 and crime (in general).
Regarding the heterogeneity in declines in auto theft, it might be useful to interpret this in terms of significant declines in auto theft in the last 20 years. The introduction of electronic immobilizers has reduced auto-theft in some places over 85% (see: Hodgkinson et al. 2016) even despite small increases in the last few years (1 or 2%). This would suggest that your baseline is so low in many countries that analysis of changing trends for this crime type would be fairly volatile.
The authors suggest that future research should examine changes in crime after the social restrictions are removed. Two papers have already done this in Australia and China:

Andresen, M.A., & Hodgkinson, T. (2020). Somehow I always end up alone: COVID-19, social isolation and crime in Queensland, Australia. Crime Science, 9, Article 25.

Borrion, H., Kurland, J., Tilley, N., & Chen, P. (2020). Measuring the resilience of criminogenic ecosystems to global disruption: A case-study of COVID-19 in China. PLoS ONE. https ://doi.org/10.20944 /prepr ints2 02006 .0309.v1.

Suggested improvements: Please list additional analyses, experiments or data that could help strengthening the work in a revision.

I have relatively few suggested improvements, besides those I've already mentioned.
1. I think it might be useful to clarify in the manuscript that assault does not include DV in any of the jurisdictions. This is an issue that can be found in some publicly available data.
2. In the future research section (p. 7 – line 298). The authors refer to "summer months" here but that is only true for the northern hemisphere. Considering the inclusion of southern hemisphere cities, this would be winter and should be noted.
3. In the future research section, you might want to discuss the need to explore these changes at the neighbourhood level across cities. You elude to this with regards to changes from city centres (pg. 7 – line 298).
4. Pg 7 (line 308), the reference to "normal people" is probably not a fair label. Perhaps the authors want to use "non-offenders" or "those not involved in organized crime."

References: Does this manuscript reference previous literature appropriately? If not, what references should be included or excluded?

The references are appropriate and not overdone. I wouldn't make any changes.

Clarity and context: Is the abstract clear, accessible? Are abstract, introduction and conclusions

appropriate?

The abstract, introduction and conclusions are all clear, accessible and appropriate.

Please indicate any particular part of the manuscript, data, or analyses that you feel is outside the scope of your expertise, or that you were unable to assess fully.
N/A

Please address any other specific question asked by the editor via email.

We are now asking authors to complete an editorial policy checklist that verifies compliance with all required editorial policies. We hope it will aid in your evaluation of the paper and we would greatly appreciate your feedback on the information provided.

Author Rebuttal to Initial comments

Dear Prof. Bradshaw and reviewers,

We are grateful for the helpful comments from the reviewers, and for the chance to revise and improve our manuscript. We are pleased to attach our revised manuscript, reflecting the edits made in light of your comments.

To summarize our edits in response to the five points made by the editor, we have:

1) provided further justification and clarification regarding both the ITS and meta-analysis and regression models used. This includes moving much of the information on data, analytical models, and supplementary analyses from the supplement to the main text.
2) We have provided further clarification regarding the use of the severity indices used. Namely, we now focus exclusively on the stay at home stringency index, and have moved the overall index to the supplement. We also provide more information about additional analyses conducted using alternative indices and measures, such as behavioural measures from the Google mobility indices.
3) We now include more information about the data and sampling strategy in the methods section, as well as relevant limitations regarding the sample and use of police-recorded data.
4) We now caution the reader regarding interpretation of effects in light of multiple hypothesis testing.
5) We now include more detailed discussion of limitations regarding the sampling strategy, the use of police-recorded data, the dark figure of crime and reporting issues, the lack of data on cybercrime and domestic violence, as well as the unit of analysis (city-level). Alongside these limitations we have made several suggestions for future research to investigate these issues and questions.

In the main and supplementary text, major changes (not including grammatical or minor language edits) are highlighted in red. To indicate where figures or tables have been changed in both the main and supplementary text, the label (e.g. 'Figure 1') has been highlighted in red.

Below we outline our edits in more detail and respond to the major suggestions and concerns from the reviewers.

Reviewer #1:
Remarks to the Author:
In this study, the authors aim at performing a globally-inspired analysis of the effects of C19 on crime across the globe. To my knowledge, this study is the first truly globally-informed analysis on how C19 has impacted crime across the planet.

I commend the authors for carefully executing what can only be described as a fantastic study. Before reviewing how this study could be improved, I will begin by reviewing what I believe are two unique strengths of this paper.

Strength 1: This paper's analysis is truly global. Up to this point in the research, studies have focused on either 1) individual cities, 2) groups of cities in the same country, and/or 3) no data at all. While I do understand that the 25 cities in this analysis are not randomly selected and hardly represent the 'total' global picture of crime, this study's purposes and goals are admirable. In short, this took a ton of work to achieve: I commend the authors for organizing such a herculean effort.

Strength 2: The findings offer new insight that is highly important. Before this article, the findings were relatively mixed as to whether there was a notable crime reduction across the globe (and in local populations, as well). The main finding prior to this paper is that there was a crime drop, but it seems like it is highly geographically situational and also dependent on the type of crime in question. With this study, we now have evidence why: The crime reductions – in one of the main findings of this paper – were temporary. The authors clearly lay out that the crime drops during lockdowns were temporary and the baseline levels were reached after a few months. Furthermore, for the most serious crime in question in this study – homicide – the drops were minimal. The findings here paint a picture that significantly advances the research on C19 and crime across the globe in both broad, sweeping ways as well as in more nuanced manners.

With this stated, I do have some recommendations for ways that the study could be improved. I review these things in the remainder of this review. First, one of the most confusing things in this

study is the two measures of C19 response severity: The overall stringency index and the severity index. Please include extra information on how both of these measures were created. Additionally, I noticed that there is a very substantial correlation between these two measures (r = .84). With this very strong correlation, I have two questions. First, is there really anything to be gained by analyzing both measures? Could this study be simplified by only presenting the findings from one of these measures? Second, I have a question about how these variables are being used in analysis. In Table 1, are both measures used in the same analysis? The reason why I ask is because it is highly unusual that the coefficient values of one of the two measures (overall stringency) is minimal.

Response: First we would like to clarify regarding the second point from the reviewer that the stay at home and stringency variables were examined in separate analyses. However, regarding the first point, we agree with the reviewer that including the overall index alongside the stay at home restrictions may detract from the focus on the effects of stay at home restrictions on effect sizes. We have now moved the overall stringency index analyses to the supplementary materials.

On this point, our intention when including the overall stringency index was to try to assess to what extent the size of the decline may be due to stay at home restrictions alone compared to the broad package of policy responses that were implemented alongside stay at home order. However, we acknowledge that the current paper cannot adequately decompose these effects, so we have included some discussion on this point in the limitations section (pgs 8-9):

"We also acknowledge that identifying the specific policy components that affected crime levels remains a challenge in macro-level comparative analyses. Across countries, a range of measures that affect the daily movement of citizens were implemented broadly at the same time. Our analyses suggest that stay at home policies played a crucial role. However, more fine-tuned analyses would be needed to understand the extent to which other measures (e.g. closing bars, limiting public transport, closing schools) and variation in their enforcement were associated with variation in crime trends across places within a city."

The coefficients of the other measure – stringency of stay at home restrictions – are much stronger in absolute value (I also understand that there are differences in scaling metrics). Since the measures are so highly correlated with each other, shouldn't the models effectively show the same thing? I am concerned here about issues of severe multicollinearity.

Response: We see now that this is not so clear in our original text. The policy variables were not included in the same analysis. We have now made this clear in the methods section of the main text (pg 11):

"Due to the small number of effect sizes included in each model and possible issues with multicollinearity, we estimated the effects of each policy variable separately."

Second, I have some questions about definitions of crimes. While things like 'homicide' are relatively timeless and universal in what they mean, things like assault are not. Many places differentiate between assault (as a serious and credible threat to person) and battery (actual physical harm). Burglary can be also combined with robbery in some places (this is dependent on local laws and police practices). Is this captured in the current analysis?

Response: *While we have not addressed this in the formal analysis, we carefully assessed the definitions prior to analysis to ensure we have the most comparable categories as possible. Where crime categories are combined (and cannot be distinguished in the data), we have excluded these cases from analyses. We provide an example in the supplement, but to make this more clear to the reader, we have moved this paragraph to the main text (pg 4):*

"Not all crime categories were available for each city, and in some contexts certain crimes are not treated as separate categories. For example, in Seoul burglary is not considered separately from robbery, and motor vehicle theft is not distinguished from theft. In order to ensure that the crime categories are as comparable as possible, we have excluded these combined outcomes from the analyses (Supplementary Tables 3-9)."

In addition, we now discuss more fully limitations related to differences between cities, including definitions, reporting, and operational priorities (pgs 8-9).

Third, on lines 280-282, the authors talk about crime displacement. This is a very important section of this paper. Can you please explain why displacement was not found? I guess that it makes sense from the perspective that if all crime is decreasing there is not displacement, more on this would be very useful, particularly among extensive speculation about feared increases in domestic and household-based violence.

Response: *We have now clarified that we refer to displacement between crime categories that we included in this paper. Neverthless, we agree that the issue of displacement is rightly important, and so we have elaborated on the question as to whether displacement to other types of crime might have occurred, including cybercrime and domestic violence.*

"An important area for future comparative research is to investigate the potential displacement of public space crimes to non-contact offences such as fraud and cybercrime, which we were unable to measure here. Studies conducted within the context of individual countries provide some evidence of displacement from public space crimes to domestic violence (32,35). There is some initial evidence of significant increase of cybercrime during the strictest period of

lockdown in the United Kingdom which is interpreted as a displacement of crime opportunities from the offline to the online environment (36). Restrictions on public space may have also lead to displacement of crime to private space. A recent meta-analysis by Piquero and colleagues suggests that there is strong evidence showing an increase of domestic violence during the pandemic using studies with multiple sources other than police reports (e.g. emergency hotline registries, health records, other administrative documents) (32). This suggests that future research should consider the impact of restriction stringency across cities and countries on the extent of shifts in crime from the public to the domestic sphere."

On lines 290-300, the authors discuss that the co-presence of offenders could have impacted the results. To be clear, co-offending was not captured in the analyses, correct? If this is the case, the authors should consider revising these comments as this analysis is not properly suited to investigate this issue.

<u>Response</u>: *We believe this may be a misunderstanding based on the wording we used – on page 7 we discuss crimes that involve the co-presence of offenders and suitable victims/targets, but by this we refer to the convergence of offenders and suitable targets, and not co-offending. In order to clarify this, we have adjusted our wording here and now state (pg 7): "We observe the largest effects for crimes that involve the convergence of motivated offenders and suitable victims/targets in public space."*

Please include a detailed discussion of limitations (e.g., police data; non-random sample; not necessarily representative of everywhere; the dark figure of crime; etc.).

<u>Response</u>: *We now include a discussion of limitations, including the use of police data, the sample composition, and reporting issues (pg 8):*

"While the results presented here extend knowledge on the impact of COVID-19 restrictions on crime across international contexts, the study is not without limitations. We acknowledge that the sample of cities included in the analyses is non-random and dominated by cities situated within Europe and the Americas. Further, relying on officially recorded crime data is associated with issues of underreporting, variations in crime definitions and operational priorities. Police records have well known problems of reporting/recording which depends on the type of crime, willingness of victims to report, how criminal justice and health agencies work and their institutional practices, which might be heterogeneous and particularly more problematic in low- and middle-income societies (31). These measurement problems might be more accentuated during the pandemic given that it might affect victims willingness to report crimes (32). Also, police responses to crime might also change because of staff absences due to COVID-19, increasing fear of contracting the virus, or even due to diversion of police resources to

alternative tasks such as enforcing the lockdown (25,30,33). However, studies that use alternative sources have partially validated our results. A recent study in Wales used Emergency Department visits for violence-related injuries to show that lockdown measures had an impact on the decrease of violence outside the home but no significant differences were observed in violent events at home (34).”

Thank you for a detailed supplemental document. I very much have enjoyed this study and it makes a significant contribution.

Reviewer #2:
Remarks to the Author:
This paper analyzes changes in crime rates in 25 cities in response to stay at home orders associated with the COVID-19 pandemic. The paper uses interrupted time series analysis to show that there were significant crime rate reductions (associated with more stringent public health measures), however variation existed across cities.

There are a number of papers that analyze similar trends in individual cities, however this paper is an important contribution as it provides the first meta analysis. The authors employ rigorous statistical methodology to analyze the drop in crime across cities in the spring of 2020 and contextualize their findings with social and criminological theory in the discussion. Overall I think this is a nice paper worthy of publication.

Below I have some specific comments that I think could improve the manuscript.

1. The authors state the dependent variable is crime “incidents.” This is incorrect, the dependent variable is reported crime incidents. This is an important distinction, as it’s possible that the pandemic led to under- or over-reporting effects not accounted for in the model.

Response: This is now clear in the text (pg. 4): “police-recorded daily reported crime incidents…”

2. Is stringency index the right independent variable to use? There is some evidence that human behavior (e.g. mobility) changes preceded public health measures:

Gupta, Sumedha, et al. Tracking public and private responses to the COVID-19 epidemic: evidence from state and local government actions. No. w27027. National Bureau of Economic Research, 2020.

Maybe using a geolocation based mobility index would provide a better predictor of the crime change response variable.

<u>Response:</u> *We acknowledge that there are multiple ways to examine the impact of the stay at home restrictions. We focus on the stay at home index because it is one of the widest available and transparent standardized indices of policy responses to Covid-19. However, given that the policy responses do not capture actual behavioral changes, we have included an additional analysis of google mobility data in the supplementary materials. The results in Supplementary Table 21 show that the size of the decline is associated with the degree of behavioral change measured by the mobility data. This is generally in line with the results using the stringency index measures. We have now made these additional analyses more clear in the main text (pg 6):*

"Further, while the stringency indices and sub-indices provide systematic and comparable measures of COVID-19 containment policies across countries, they do not provide a measure of actual behavioral changes. We therefore conducted additional analyses to assess the relationship between changes in mobility indices as measured by the Google COVID Community Mobility Reports (23,24), and effect sizes for each crime type. Bivariate correlations between mobility measures and stringency measures suggest that more stringent stay at home restrictions are associated with greater declines in visits to commercial locations and parks, as well as increases in users remaining in their residences (Supplementary Table 12). The results using mobility indices are generally in line with the results using the stringency index measures, whereby cities that saw greater declines in the use of public space saw larger declines in crime, with the exception of homicide (Supplementary Table 21)."

3. The supplemental material seems a bit long and un-organized. I would prefer the methodological material up front rather than variable definitions for each city. Or, maybe more details on the ITS model could be provided in the main text?

<u>Response:</u> *We have restructured the supplementary materials and methodological information in the main text. There are four major changes:*

1. *We have now moved information on the data and methods for both the main text and additional analyses to the Methods section in line with Nature HB formatting requirements (following the discussion section).*
2. *We have structured the results section in the main text by the two major analyses, indicated by subheadings ('the impact of stay at home restrictions on crime' for the ITS analyses and 'the severity of restrictions and the size of decline' for the meta-regressions). Within each of these sections, we have moved relevant information on the data and analytical approach.*
3. *We have moved more information on the data and variables from the supplement to the main text (Methods section).*
4. *We have added a section in the results on 'additional analyses' that summarizes the sensitivity analyses using other COVID-19 policy variables and Google mobility indices.*

I think Figure 1 can be improved with better labeling. It was a little unclear what day 0 is, but I think it is the day stay at home measures were implemented. If this is the case, I think it would be better to have a vertical line with a text label indicating stay at home measures were implemented. I would also include 50-100 days prior (like is done in the supplemental material), because the drop is fairly dramatic when visualized in this way. Also, the y-axis label is confusing since it says number of offenses but they all start at 100. Are the time series normalized or just shifted? This should be stated in the figure.

<u>Response</u>: *We have revised the labelling for the moving average figures, which now say "Indexed 7-day moving average of offences | Index=100 at date SaH implemented." Hopefully this is clearer to the reader that the moving average is indexed at 100 at the date the stay at home restrictions were implemented. We have also expanded the x-axis timeline to include 30 days prior to the implementation of stay at home restrictions and added a vertical line at t=0, so the figures now show before and after the restrictions (Figure 1).*

5. Does the meta-analytic technique in Figure 2 control for multiple hypothesis testing? If so that should be explained in the caption. If not, then the statistical significance should be interpreted accordingly. Same for Figure 3.

<u>Response</u>: *The reviewer makes a good point, and we have now included a statement about interpreting the individual city results with caution (pg 5):*

"The high number of hypotheses tested increases the possibility that we may detect a significant result due to chance. We therefore urge caution in interpreting individual city results."

6. Why is a quadratic fit used in Figure 4A and linear in 4b? Was some type of model selection criteria used and if so what was the criteria? Uncertainty in the slope should also be shown in these figures.

<u>Response</u>: *Since moving the overall stringency index results to the supplement, Figure 4 now only presents the scatterplot for stay at home restrictions and average crime decline. Given that we examine a linear relationship in the meta-regressions, we include a line reflecting the linear fit between the two variables, along with confidence intervals.*

Reviewer #3:
Remarks to the Author:

Nonetheless, I have a few comments/questions for the authors:

1. Why is Poisson used? It appears that you don't find over-dispersion, but an over-dispersion variable is included. Some clarification is necessary here.

Response: *In section 2.4 of the supplement we now include more discussion about the model selection for ITS analyses. In essence, Poisson models were the most flexible for the large variety of outcomes and levels of crime. On page 44 we state:*

"While the data are all count, the number of daily crimes differ across crime types and cities (i.e. from 0 to >500 per day). We aimed for a flexible modelling approach with which we could estimate comparable estimates of effect across models, and that would be appropriate for modelling both low and high counts of daily crime. We therefore opted for a Poisson Generalized Linear Model [GLM] with a logit-link function, as this approach is the most common for handling time series count data, as well as the number of crime counts (40-41). […]

All models include an offset for population and are adjusted for autocorrelation based on the examination of the residuals. In addition, given that in some cases the data tended to be overdispersed, we included in all models an adjustment to estimate the appropriate standard errors as recommended in epidemiological studies (40)."

2. The authors make reference to a lot of zero counts (not surprising with daily data or with using homicide) – why not use a zero-inflated model?

Response: *We agree that zero-inflated models are a possible solution to examining more rare types of crime such as homicide. However, for the purpose of the current analysis, we were primarily interested in estimating the change in average number of daily crimes due to restrictions, for which a Poisson GLM regression is better suited (see also comment to point 1 above).*

3. Is the severity of stay at home restrictions variable entered in the analysis separately? It isn't clear in the description of the data. If it is, does it make sense for this variable to be ordinal? This should at least be tested and reported.

Response: *The stringency index and stay at home index variables were not included in the same equation/model. In response to Reviewer 1, we have also now edited the main text so the analyses focus on stay at home restrictions only, moving the overall stringency index analyses to the supplement. We now make this point clear in the supplement on page 15: "Due to the small number of effect sizes included in each model and possible issues with multicollinearity, we estimated the effects of each policy variable separately."*

4. An equation showing what was estimated would be useful to answer some of these questions. If I haven't missed it, I would strongly suggest that an equation for the model that is estimated is included.

<u>Response:</u> *In section 2.4 of the supplementary materials, we now include additional information on the models used for the interrupted time series analyses (pg 44):*

"[…] The baseline Poisson regression model with treatment effect can be expressed as (41):

$$log(E(y|\lambda)) = \alpha + x_T\beta_T + x_k\beta_k$$

Where y reflects the expected count of daily crime per category, dependent on the expected rate of the crime outcome (λ) based on a Poisson distribution. The expected outcome is a function of the intercept (α), the treatment variable (x_T) and a set of covariates (x_k). The variable x_T reflects the 'treatment' variable expressed as a step function, whereby 0 represents the period prior to (or following) the implementation of restrictions and 1 represents the period in which restrictions were in place. The models also include a vector of covariates (x_k), including daily temperature, time trend, seasonal dummy variables, and dummy variables for any holidays or outliers."

The conclusions are straightforward, robust, valid and reliable. The authors found that more severe restrictions on non-essential movement led to larger declines in crime (with some differences in model fit). These do not lead to changes in effect sizes (save for public transit restrictions and vehicle theft). The researchers also recognize the limitations of not being able to examine cyber-crime, which is a common limitation in this research area. Interpretations are consistent with results and with the current findings in the broader literature on COVID-19 and crime (in general).

Regarding the heterogeneity in declines in auto theft, it might be useful to interpret this in terms of significant declines in auto theft in the last 20 years. The introduction of electronic immobilizers has reduced auto-theft in some places over 85% (see: Hodgkinson et al. 2016) even despite small increases in the last few years (1 or 2%). This would suggest that your baseline is so low in many countries that analysis of changing trends for this crime type would be fairly volatile.

Response: *This is a good point, and we now acknowledge this decline as well as the possible interaction between the pandemic and previous declines (pg 9):*

"Finally, it is important to emphasise that the impact of COVID-19-related containment policies on crime trends must be considered within the broader context of global declines in some types of crime, including homicide (37-41) and vehicular theft (42) allied with increases in technology-facilitated offences and the potential accelerating effect of the pandemic on this trend."

The authors suggest that future research should examine changes in crime after the social restrictions are removed. Two papers have already done this in Australia and China:

Andresen, M.A., & Hodgkinson, T. (2020). Somehow I always end up alone: COVID-19, social isolation and crime in Queensland, Australia. Crime Science, 9, Article 25.

Borrion, H., Kurland, J., Tilley, N., & Chen, P. (2020). Measuring the resilience of criminogenic ecosystems to global disruption: A case-study of COVID-19 in China. PLoS ONE. https://doi.org/10.20944 /prepr ints2 02006 .0309.v1.

Response: *We have now included these important papers in our discussion on crime trends after restrictions (pg 7):*

"This aligns with previous research conducted in Australia (25) and China (26) that found immediate declines in public space crimes such as theft, burglary and traffic offences experienced during lock down periods quickly reversed as restrictions eased."

I have relatively few suggested improvements, besides those I've already mentioned.

1. I think it might be useful to clarify in the manuscript that assault does not include DV in any of the jurisdictions. This is an issue that can be found in some publicly available data.

Response: *We made every attempt where possible to exclude domestic violence incidents from daily assault cases, however in certain cities it is not possible to distinguish domestic and non-domestic assaults in police data (Amsterdam, Helsinki, Toronto, Tallinn, London). In order to assess whether these cases may be influencing the results, we re-estimated the meta-regressions (including and excluding the outlier Barcelona) without these cities. The results are not influenced by the exclusion of these cities, and are presented in the supplementary materials*

(Supplementary Table 19). We now also make note of this sensitivity analysis, as well as other sensitivity analyses, in the methods section in the main text (pg 6).

2. In the future research section (p. 7 – line 298). The authors refer to “summer months” here but that is only true for the northern hemisphere. Considering the inclusion of southern hemisphere cities, this would be winter and should be noted.

Response: *The language here has been adjusted: “…during June, July and August of 2020…”*

3. In the future research section, you might want to discuss the need to explore these changes at the neighbourhood level across cities. You elude to this with regards to changes from city centres (pg. 7 – line 298).

Response: *This is a good point, and we have now included some discussion of neighborhood-level variations along with calls for future research (pg 8):*

“We would expect, for example, that a distinction of assault cases by place would reveal that assault in the hotspots of weekend night-time activities declined more where the lockdown was more stringent, while violence in domestic contexts may not have declined or even have increased. Our results might be hiding a more complex picture characterized by neighbourhood heterogeneity both in terms of the independent and dependent variables. Research in Chicago shows that there is there is heterogeneity in the impact of containment policies across communities and only a small percentage of communities experienced significant reductions in crimes with variation depending on the type of crime (e.g. burglaries, assaults, narcotic-related offenses, robberies) and community crime characteristics (e.g. previous levels of offenses, perception of safety, presence of police station) socio economic characteristics (vacant housing, income diversity, poverty, age structure of neighbours) and self-perceived health of neighbours (28,29). What is more, research in India has shown that the higher stringency of lockdown restriction across city districts is associated with lower levels of economically motivated crimes and higher level of violence against women (30). Further research on variations within cities and at micro- places is needed in order to provide further insights into the moderating effect of local contexts on the effects of COVID-19 restrictions on crime.”

4. Pg 7 (line 308), the reference to “normal people” is probably not a fair label. Perhaps the authors want to use “non-offenders” or “those not involved in organized crime.”

Response: *The language here has been adjusted: “…those not involved in organized crime.”*

Decision Letter, first revision:

** Please ensure you delete the link to your author homepage in this e-mail if you wish to forward it to your co-authors. **

Our ref: NATHUMBEHAV-210113795A

22nd April 2021

Dear Dr. Nivette,

Thank you for your patience as we've prepared the guidelines for final submission of your Nature Human Behaviour manuscript, "A global analysis of the impact of COVID-19 stay at home restrictions on crime" (NATHUMBEHAV-210113795A). Please carefully follow the step-by-step instructions provided in the attached file, and add a response in each row of the table to indicate the changes that you have made. Please also check and comment on any additional marked-up edits we have proposed within the text. Ensuring that each point is addressed will help to ensure that your revised manuscript can be swiftly handed over to our production team.

We would like to start working on your revised paper, with all of the requested files and forms, as soon as possible (preferably within two weeks). Please get in contact with us if you anticipate delays.

When you upload your final materials, please include a point-by-point response to any remaining reviewer comments.

If you have not done so already, please alert us to any related manuscripts from your group that are under consideration or in press at other journals, or are being written up for submission to other journals (see: https://www.nature.com/nature-research/editorial-policies/plagiarism#policy-on-duplicate-publication for details).

Nature Human Behaviour offers a Transparent Peer Review option for new original research manuscripts submitted after December 1st, 2019. As part of this initiative, we encourage our authors to support increased transparency into the peer review process by agreeing to have the reviewer comments, author rebuttal letters, and editorial decision letters published as a Supplementary item. When you submit your final files please clearly state in your cover letter whether or not you would like to participate in this initiative. Please note that failure to state your preference will result in delays in accepting your manuscript for publication.

In recognition of the time and expertise our reviewers provide to Nature Human Behaviour's editorial process, we would like to formally acknowledge their contribution to the external peer review of your manuscript entitled "A global analysis of the impact of COVID-19 stay at home restrictions on crime". For those reviewers who give their assent, we will be publishing their names alongside the published article.

Cover suggestions

As you prepare your final files we encourage you to consider whether you have any images or illustrations that may be appropriate for use on the cover of Nature Human Behaviour.

Covers should be both aesthetically appealing and scientifically relevant, and should be supplied at the best quality available. Due to the prominence of these images, we do not generally select images featuring faces, children, text, graphs, schematic drawings, or collages on our covers.

We accept TIFF, JPEG, PNG or PSD file formats (a layered PSD file would be ideal), and the image should be at least 300ppi resolution (preferably 600-1200 ppi), in CMYK colour mode.

If your image is selected, we may also use it on the journal website as a banner image, and may need to make artistic alterations to fit our journal style.

Please submit your suggestions, clearly labeled, along with your final files. We'll be in touch if more information is needed.

ORCID

Non-corresponding authors do not have to link their ORCIDs but are encouraged to do so. Please note that it will not be possible to add/modify ORCIDs at proof. Thus, please let your co-authors know that if they wish to have their ORCID added to the paper they must follow the procedure described in the following link prior to acceptance: https://www.springernature.com/gp/researchers/orcid/orcid-for-nature-research

Nature Human Behaviour has now transitioned to a unified Rights Collection system which will allow our Author Services team to quickly and easily collect the rights and permissions required to publish your work. Approximately 10 days after your paper is formally accepted, you will receive an email in providing you with a link to complete the grant of rights. If your paper is eligible for Open Access, our Author Services team will also be in touch regarding any additional information that may be required to arrange payment for your article. Please note that you will not receive your proofs until the publishing agreement has been received through our system.

Please note that <i>Nature Human Behaviour</i> is a Transformative Journal (TJ). Authors may publish their research with us through the traditional subscription access route or make their paper immediately open access through payment of an article-processing charge (APC). Authors will not be required to make a final decision about access to their article until it has been accepted. Find out more about Transformative Journals

<B>Authors may need to take specific actions to achieve compliance with funder and institutional open access mandates. For submissions from January 2021, if your research is supported by a funder that requires immediate open access (e.g. according to Plan S principles) then you should select the gold OA route, and we will direct you to the compliant

route where possible. For authors selecting the subscription publication route our standard licensing terms will need to be accepted, including our self-archiving policies. Those standard licensing terms will supersede any other terms that the author or any third party may assert apply to any version of the manuscript.

For information regarding our different publishing models please see our Transformative Journals page. If you have any questions about costs, Open Access requirements, or our legal forms, please contact ASJournals@springernature.com.

Please use the following link for uploading these materials:
[REDACTED]

If you have any further questions, please feel free to contact me.

Best regards,
Chloe Knight
Editorial Assistant
Nature Human Behaviour

On behalf of

Aisha Bradshaw
Editor
Nature Human Behaviour

Reviewer #1:
Remarks to the Author:
Like the preceding version of this study, this study is absolutely outstanding (again). I applaud the authors for successfully navigating not only my comments but also the other reviewers' comments.

I am of the impression that this revision is adequate. The supplement, manuscript, and the clarity are all greatly improved. Instead of offering further minor changes that will inevitably delay publication, I am of the opinion that this study should be published in its current form in an expeditious manner due to the importance of the findings. The only thing that I would like to see is to resolve the lack of commas in the sentence on lines 344-347.

The findings of this study are of overall great importance and I can't wait to see the media attention this study receives. I commend the authors for performing some simply outstanding research!

Reviewer #2:

Remarks to the Author:
The authors have addressed all of my comments. Congrats on a very nice paper.

Reviewer #3:
Remarks to the Author:
I'm happy with the revisions that have been made and believe the paper should be accepted for publication.

Author Rebuttal, first revision:

Dear Prof. Bradshaw and reviewers,

We are happy to see that the reviewers were satisfied with our revisions. We have addressed the only comment from Reviewer #1 about including commas on lines 344-347 (pg 8). The remaining changes were made in light of the checklist containing guidelines for the final submission.

To summarize the major edits in light of the checklist, we have:

1. Provided confirmation of the author list from all co-authors. Please note that small amendments were made to the authors' names and affiliations based on their preference/request. These changes are marked in red.
2. Adjusted the numbering of the supplementary figures, and subsequent references to supplementary tables and figures in the main text.
3. Added appropriate labelling, number of observations, and reference to full results for relevant figures.
4. Added 95% confidence intervals where relevant.
5. Made data and statistical code used in the analyses available in a repository for future use.
6. Included a table with a description of the data source for each city.
7. Provided relevant author contribution, acknowledgements, data availability, funding, and COI statements.
8. Provided a brief summary of the main findings of the paper.

Final Decision Letter:

Dear Amy,

Thank you for submitting your updated manuscript files. I am happy to inform you that your Article "A global analysis of the impact of COVID-19 stay at home restrictions on crime", has now been accepted for publication in Nature Human Behaviour.

Before your manuscript is typeset, we will edit the text to ensure it is intelligible to our wide readership and conforms to house style. We look particularly carefully at the titles of all papers to ensure that they are relatively brief and understandable.

Once your manuscript is typeset and you have completed the appropriate grant of rights, you will receive a link to your electronic proof via email with a request to make any corrections within 48 hours. If, when you receive your proof, you cannot meet this deadline, please inform us at rjsproduction@springernature.com immediately. Once your paper has been scheduled for online publication, the Nature press office will be in touch to confirm the details.

Acceptance of your manuscript is conditional on all authors' agreement with our publication policies (see http://www.nature.com/nathumbehav/info/gta). In particular your manuscript must not be published elsewhere and there must be no announcement of the work to any media outlet until the publication date (the day on which it is uploaded onto our web site).

Please note that <i>Nature Human Behaviour</i> is a Transformative Journal (TJ). Authors may publish their research with us through the traditional subscription access route or make their paper immediately open access through payment of an article-processing charge (APC). Authors will not be required to make a final decision about access to their article until it has been accepted. Find out more about Transformative Journals

<B>Authors may need to take specific actions to achieve compliance with funder and institutional open access mandates. For submissions from January 2021, if your research is supported by a funder that requires immediate open access (e.g. according to Plan S principles) then you should select the gold OA route, and we will direct you to the compliant route where possible. For authors selecting the subscription publication route our standard licensing terms will need to be accepted, including our self-archiving policies. Those standard licensing terms will supersede any other terms that the author or any third party may assert apply to any version of the manuscript.

If you have posted a preprint on any preprint server, please ensure that the preprint details are updated with a publication reference, including the DOI and a URL to the published version of the article on the journal website.

An online order form for reprints of your paper is available at https://www.nature.com/reprints/author-reprints.html. All co-authors, authors' institutions and authors' funding agencies can order reprints using the form appropriate to their geographical region.

We welcome the submission of potential cover material (including a short caption of around 40 words) related to your manuscript; suggestions should be sent to Nature Human Behaviour as electronic files (the image should be 300 dpi at 210 x 297 mm in either TIFF or JPEG format). Please note that such

pictures should be selected more for their aesthetic appeal than for their scientific content, and that colour images work better than black and white or grayscale images. Please do not try to design a cover with the Nature Human Behaviour logo etc., and please do not submit composites of images related to your work. I am sure you will understand that we cannot make any promise as to whether any of your suggestions might be selected for the cover of the journal.

You can now use a single sign-on for all your accounts, view the status of all your manuscript submissions and reviews, access usage statistics for your published articles and download a record of your refereeing activity for the Nature journals.

To assist our authors in disseminating their research to the broader community, our SharedIt initiative provides you with a unique shareable link that will allow anyone (with or without a subscription) to read the published article. Recipients of the link with a subscription will also be able to download and print the PDF.

As soon as your article is published, you will receive an automated email with your shareable link.

In approximately 10 business days you will receive an email with a link to choose the appropriate publishing options for your paper and our Author Services team will be in touch regarding any additional information that may be required.

You will not receive your proofs until the publishing agreement has been received through our system.

If you have any questions about our publishing options, costs, Open Access requirements, or our legal forms, please contact ASJournals@springernature.com

We look forward to publishing your paper.

With best regards,
Aisha

Aisha Bradshaw
Editor
Nature Human Behaviour

P.S. Click on the following link if you would like to recommend Nature Human Behaviour to your librarian http://www.nature.com/subscriptions/recommend.html#forms

** Visit the Springer Nature Editorial and Publishing website at www.springernature.com/editorial-and-publishing-jobs for more information about our career opportunities. If you have any questions please click here.**
